# SNP-RFLPing 2: an updated and integrated PCR-RFLP tool for SNP genotyping

**DOI:** 10.1186/1471-2105-11-173

**Published:** 2010-04-08

**Authors:** Hsueh-Wei Chang, Yu-Huei Cheng, Li-Yeh Chuang, Cheng-Hong Yang

**Affiliations:** 1 Department of Biomedical Science and Environmental Biology, Kaohsiung Medical University, Kaohsiung, Taiwan; 2 Graduate Institute of Natural Products, College of Pharmacy, Kaohsiung Medical University, Kaohsiung, Taiwan; 3 Center of Excellence for Environmental Medicine, Kaohsiung Medical University, Kaohsiung, Taiwan; 4 Cancer Center, Kaohsiung Medical University Hospital, Kaohsiung Medical University, Kaohsiung, Taiwan; 5 Department of Electronic Engineering, National Kaohsiung University of Applied Sciences, Kaohsiung, Taiwan; 6 Department of Chemical Engineering, I-Shou University, Kaohsiung, Taiwan; 7 Department of Network Systems, Toko University, Chiayi, Taiwan

## Abstract

**Background:**

PCR-restriction fragment length polymorphism (RFLP) assay is a cost-effective method for SNP genotyping and mutation detection, but the manual mining for restriction enzyme sites is challenging and cumbersome. Three years after we constructed SNP-RFLPing, a freely accessible database and analysis tool for restriction enzyme mining of SNPs, significant improvements over the 2006 version have been made and incorporated into the latest version, SNP-RFLPing 2.

**Results:**

The primary aim of SNP-RFLPing 2 is to provide comprehensive PCR-RFLP information with multiple functionality about SNPs, such as SNP retrieval to multiple species, different polymorphism types (bi-allelic, tri-allelic, tetra-allelic or indels), gene-centric searching, HapMap tagSNPs, gene ontology-based searching, miRNAs, and SNP500Cancer. The RFLP restriction enzymes and the corresponding PCR primers for the natural and mutagenic types of each SNP are simultaneously analyzed. All the RFLP restriction enzyme prices are also provided to aid selection. Furthermore, the previously encountered updating problems for most SNP related databases are resolved by an on-line retrieval system.

**Conclusions:**

The user interfaces for functional SNP analyses have been substantially improved and integrated. SNP-RFLPing 2 offers a new and user-friendly interface for RFLP genotyping that can be used in association studies and is freely available at http://bio.kuas.edu.tw/snp-rflping2.

## Background

Single nucleotide polymorphisms (SNPs) are the most abundant variants in many genomes, and are very important in many fields of genomics. Many high-throughput SNP genotyping methods have been developed including SNP microarrays [1], MALDI-TOF (Matrix Assisted Laser Desorption/Ionization-Time of Flight) [2], TaqMan probes [3], PCR resequencing [4], and others [5-7]; however, most of them come at a price. This is that they require expensive machinery and are unsuitable for the kind of small-scale genotyping that is routinely undertaken in most regular laboratories. TaqMan real-time PCR, for instance, has been applied to SNP genotyping in many association studies [3,8,9] but some SNPs cannot be genotyped using TaqMan probes [10-12]. As an alternative, PCR-restriction fragment length polymorphism (RFLP) analysis is able to provide cost-effective SNP genotyping and mutation detection [12-14]. However, RFLP mining for SNPs in a genome is a complex task when manually performed.

Many SNP- or restriction enzyme-related software tools, such as SNPicker [15], NEBcutter [16], SRP Opt [17], PIRA-PCR Designer [18], SNP cutter [19], SNPselector [20], SNP2CAPS [21], and software for "Restriction of DNA sequences" [22] have been reviewed and compared to SNP-RFLPing (ver. 1) [23]. Other more recent SNP-related software tools have also been developed, such as SNP500Cancer [24], which provides gene-centric SNP retrieval with TaqMan probe information but only for the SNPs associated with human cancer-related genes. Another is Seq4SNPs [25], which provides retrieval of multiple accurately annotated DNA sequences that have already been formatted for SNP assay design. SNP-Flankplus [26] provides SNP ID-centric retrieval functions for SNP flanking sequences. SNPLogic [27] provides comprehensive SNP selection, annotation and a prioritization system for genotyping. SNP ID-info [28] as well as a visual SNP ID platform for multiple inputs can be used to improve systematic SNP association studies. However, none of these software platforms provide RFLP information that can be used as part of SNP genotyping.

In 2006, we developed SNP-RFLPing (ver. 1) [23], an efficient and informative PCR-RFLP mining tool for SNPs, which provided user-friendly multiple sequence input formats, gene-centric RFLP assays for SNPs, and detailed output of the SNP information. To the best of our knowledge, SNP-RFLPing (ver. 1) was the first software to link the gene name to its SNP-RFLP restriction enzyme information. In the past three years, the numbers of known SNPs [29] and their RFLP restriction enzymes [30] have accumulated rapidly for the genomes of many species. Many new resources, such as tagSNP [31], miRNA [32], and gene ontology (GO) [33] are related to the study of SNP but display a relatively small amount of the relevant information needed for SNP genotyping. This paper presents an update, SNP-RFLPing 2, of SNP-RFLPing (ver. 1), which acts as a software tool that introduces and integrates a PCR-RFLP database for SNP genotyping.

## Implementation

### Web interface

**Figure 1:**
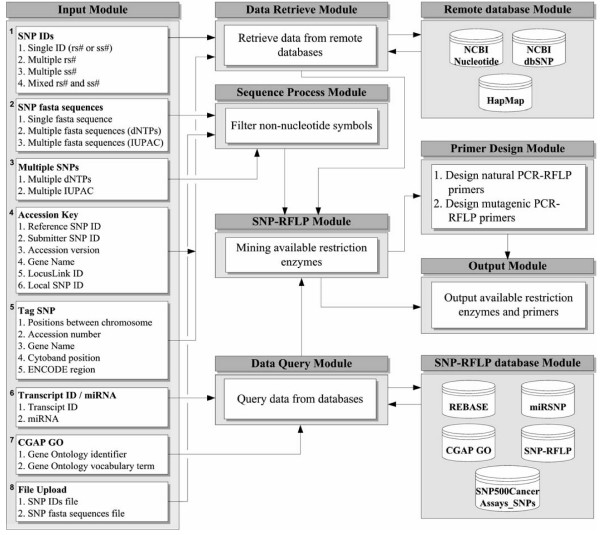
**System structure and flowchart for SNP-RFLPing 2**.

The flow chart for the eight input functions, namely: (1) SNP IDs, (2) SNP fasta sequences, (3) multiple SNPs, (4) accession key, (5) tag SNP, (6) transcript ID/miRNA, (7) CGAP GO, and (8) file upload, is shown in Figure 1. Their corresponding processes are performed as indicated by arrow lines. For inputs of SNP IDs, accession key and tag SNP, the "Data Retrieve Module" is designed to retrieve data from remote databases, such as NCBI nucleotide, NCBI dbSNP, and HapMap, into the "Remote Database Module". For inputs of SNP fasta sequences, multiple SNPs, and file upload, the "Sequence Process Module" is designed to filter non-nucleotide symbols, including blanks, commas, and brackets. For inputs of transcript ID/miRNA and CGAP GO (gene ontology in the Cancer Genome Anatomy Project), the "Data Query Module" is designed to query available information from the "SNP-RFLP database Module". After the "Remote Database Module", "Sequence Process Module", or "Data Query Module" processes are performed, the remote data (NCBI nucleotide, NCBI dbSNP, and HapMap), the filtered sequence data (without non-nucleotide symbols), and query data from SNP-RFLP database (REBASE, CGAP GO, miRSNP, SNP-RFLP, and SNP500Cancer Assays_SNPs) are fed into the "SNP-RFLP Module", which then mines the data for the available restriction enzyme sites as appropriate. Subsequently, PCR-RFLP primers can be designed using the "Primer Design Module". Finally, the results for the available restriction enzymes and PCR-RFLP primers are output by the "Output Module". Detailed information on the databases used by SNP-RFLPing is outlined below.

### Database description

All the PCR-RFLP functions in SNP-RFLPing 2 provide both the RFLP restriction enzymes and primer set for PCR-RFLP. The primer design function is partly based on the Prim-SNPing [34], that is the regular and mutagenic (degenerate) primer designs for PCR-RFLP are provided. Moreover, the price of the possible RFLP enzymes is a newly added feature and RFLP enzymes are now classified into IUPAC and non-IUPAC (recognition sequences containing only the combination of nucleotides A, T, C, and G) types. Some SNP IDs that have pre-designed TaqMan probes from SNP500Cancer [24] and from dbSNP in NCBI (ABI) are integrated into SNP-RFLPing 2. SNP-RFLPing (ver. 1) only provides RFLP restriction enzyme information.

The flanking sequences for SNP ID rs# and ss# input for all available species are designed to be extendable. They are retrieved on-line from SNP-Flankplus [26] with modifications of the updated retrieval source codes to adapt the current format to dbSNP [29] build 129 from NCBI. The International HapMap Project provides the tagSNPs in the human genome for several HapMap populations such as YRI (Yoruba in Ibadan, Nigeria), JPT (Japanese in Tokyo, Japan), CHB (Han Chinese in Beijing, China), and CEU (CEPH; Utah residents with ancestry from northern and western Europe) [31]. The current on-line linked tagSNP database is HapMap Data Rel 23a/phaseII Mar08, on the NCBI B36 assembly, dbSNP b126 [31]. The miRNA SNPs are downloaded from the Polymorphism in the microRNA Target Site (PolymiRTS) database [32], which includes naturally occurring DNA variations in putative microRNA target sites.

### Availability and update frequency

The SNP-RFLPing 2 web site and its user manual are freely accessible at http://bio.kuas.edu.tw/snp-rflping2 (or Additional file 1) and http://bio.kuas.edu.tw/snp-rflping2/userManual.jsp, respectively. The RFLP restriction enzymes and the corresponding primers for all SNPs are simultaneously analyzed on-line. For REBASE version 906 [30], SNP for miRNA (downloaded from PolymiRTS Database) [32], and GO Browser (CGAP GO), they are updated annually and are built into a local database (SNP-RFLP database module). All other databases, such as dbSNP, HapMap, and GenBank, are constantly updated and retrieved on-line by an automated procedure. To improve the speed of access, our application encodes the user request information and sends them to the appropriate remote application server. The process is almost the same as when these remote applications are performed on their web sites. During data processing, the system retrieves the query or analysis result page from the remote application servers by http transmission. This does slightly increase time lag, which is usually less than one second, because the application needs to parse the result page and obtain the useful information before transforming it into the appropriate format for further processing. All the aforementioned steps are cached and performed on the server side. The retrieval formats for these on-line databases will be checked monthly in order to maintain the correct on-line extraction formats.

## Results and discussion

### Summary of the database updates

In parallel to the rapid expansion of the use of SNPs in the last few years, SNP-RFLPing 2 demonstrates significant advances over SNP-RFLPing (ver. 1). These include: 1) rewritten source codes to improve the functionality, efficiency and stability of SNP-RFLP analysis; 2) SNPs for sixteen different species can be retrieved on-line; 3) all kinds of SNPs are acceptable including di-, tri-, tetra-allelic and indel formats; 4) the functional class (function codes for reference SNP clusters or refSNPs in gene features used for options to limit retrieval) for dbSNP in NCBI has been added (details on functional class are discussed later); 5) a multiple SNPs-containing sequence for PCR-RFLP mining is acceptable; 6) HapMap tagSNPs for multiple inputs can be retrieved on-line; 7) gene ontology (GO)-based RFLP enzyme mining is provided; 8) miRNA SNPs for the human and mouse genomes are included; 9) regular and degenerate primer designs for PCR-RFLP are provided; 10) REBASE databases are updated and the prices of RFLP restriction enzymes are available; 11) RFLP enzymes are classified into IUPAC and non-IUPAC types; and 12) TaqMan probes for SNP genotyping from SNP500Cancer in CGAP and from dbSNP in NCBI (ABI) are supplied if available.

### I. Original functions in SNP-RFLPing (ver. 1) and their improvements

#### SNP ID input, SNP in fasta sequence input, and file upload

**Figure 2:**
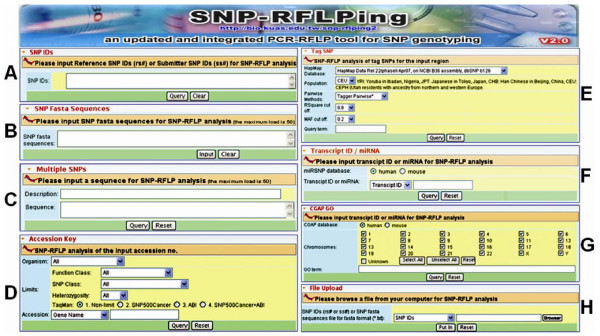
**Overview of the SNP-RFLPing 2 web interface**. (A) SNP ID input. (B) SNP in fasta sequence input. (C) Multiple SNPs within one sequence input. (D) GenBank accession input. (E) TagSNP from HapMap input. (F) Transcript ID/miRNA input. (G) Gene Ontology-based annotation for SNPs input. (H) File upload input.

SNP ID (rs# and ss#) and SNP in fasta sequence formats are acceptable to query the SNP-RFLP information (Figure 2A and Figure 2B, respectively). Two types of files are acceptable for file upload, namely SNP IDs (rs#, ss#, or mixture of rs#/ss#) and SNP fasta sequences (multiple sequences with SNPs in [dNTP1/dNTP2] or IUPAC formats) (Figure 2H). These functions were also originally included in SNP-RFLPing (ver. 1).

In SNP-RFLPing 2, we have newly added a function for RFLP analysis and primer design involving tri-allelic, tetra-allelic, and indel (insertion and deletion) SNPs, such as, for example, rs2243244, rs13631133, and rs68134313 respectively. Furthermore, SNP ID information for all available species can be retrieved on-line using SNP-RFLPing 2 rather than from the local database that was built into SNP-RFLPing (ver. 1), that is sixteen genomes (current data)*vs*. three genomes (human, mouse, and rat) respectively.

#### GenBank Accession

The inputs for the GenBank accession no. [35], such as reference SNP ID (rs#), submitter SNP ID (ss#), HUGO gene name, and local link ID (gene ID), which can be used to retrieve the SNP sequence information for RFLP analysis, are the same as the original functions in SNP-RFLPing (ver. 1).

In SNP-RFLPing 2 (Figure 2D) additional input formats for accession version and local SNP ID have been added. The classification of dbSNP in NCBI for functional class (coding nonsynonymous, reference, intron, coding synonymous, locus region, mRNA UTR, and splice site), SNP class (heterozygous, indel, mixed, multinucleotide polymorphism, named locus, no variation, and snp), and heterozygosity are selectable in GenBank accession input. Furthermore, the entire information contained in GenBank can be retrieved on-line for all available species and this is integrated in SNP-RFLPing 2.

### II. Added improvements to SNP-RFLPing 2

#### Multiple SNPs within one sequence

Up to 50 SNPs represented in the [dNTP1/dNTP2] or IUPAC formats within an input sequence are acceptable for analysis in SNP-RFLPing 2 (Figure 2C). The flanking sequences for two nearby SNPs should not overlap within a range of 6 nucleotides. The pre-aligned reference sequence, such as that generated by the multiple sequence alignment function in Seq-SNPing [36], may be input into the SNP-RFLPing 2 for RFLP mining of multiple SNPs as well.

#### TagSNP from HapMap

To reduce the necessary amount of SNPs for genotyping, it was believed that a subset of the SNPs in a region (tagSNPs) ought be chosen to represent most of the remaining SNP variants [37]. As shown in Figure 2E, the HapMap database versions, population, pairwise methods (tagger pairwise or tagger multimarkers), R square cutoff, and MAF (minor allele frequency) cutoff can all be user-adjusted in SNP-RFLPing 2. The position within the chromosome, accession number, gene name, cytoband position, and ENCODE (ENCyclopedia Of DNA Elements) [38] region can also be queried. The tagSNPs information from HapMap is retrieved online and the mining function of RFLP restriction enzymes for tagSNPs is implemented in SNP-RFLPing 2.

#### Transcript ID/miRNA

MicroRNAs (miRNAs) are a group of small RNAs that are able to bind to the RNA transcripts of protein-coding genes and this allows them to repress translation or decrease mRNA stability [39,40]. Dysfunction of miRNAs influences cell biology and cancer progression [41]. Polymorphisms in miRNA pathways may affect gene expression, which may lead to a change in complex phenotypes, and such polymorphisms have the potential to be disease markers for personalized medicine [42]. Transcript IDs and miRNA numbers from the human and mouse datasets are acceptable for PCR-RFLP analysis (Figure 2F). The RFLP enzyme mining and primer design for transcript IDs and miRNA are newly developed and have been integrated in the SNP-RFLPing 2.

#### Gene Ontology-based annotation for SNPs

The GeneOntology Browser (GO Browser; http://cgap.nci.nih.gov/Genes/GOBrowser), which provides annotations for human and mouse genes based on molecular function, biological process, and cellular component, has been integrated into SNP-RFLPing 2. GO IDs and vocabulary terms may be input to find specific genes with an interesting function as well as their corresponding SNPs (Figure 2G).

### III. Common output examples for SNP-RFLPing 2

**Figure 3:**
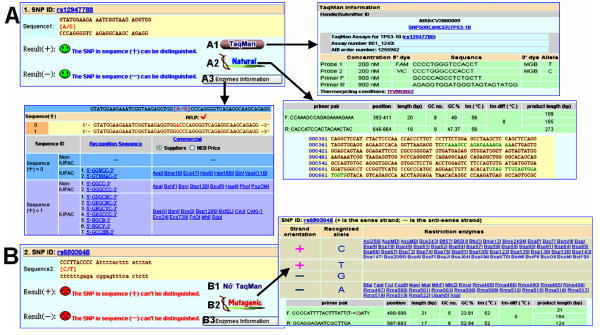
**Representative outputs of SNP-RFLPing 2**. (A) Natural primers for rs12947788. (B) Mutagenic primers for rs6503048. SNP genotyping information for (A1/B1) TaqMan probe, (A2/B2) natural primers, (A3/B3) restriction enzymes.

Two types of SNP genotyping information, such as primers (natural and mutagenic)/restriction enzymes using PCR-RFLP and TaqMan probes using real-time PCR (Figure 3), are provided for all the inputs in SNP-RFLPing 2 (Figure 2). Except for the restriction enzymes, the other functions are novel improvements found only in SNP-RFLPing 2. For TaqMan probes, these are shown as available and unavailable in the examples given for rs12947788 and rs650304, which are shown in Figures 3A1 and 3B1 respectively. The TaqMan probes are on-line retrieved from SNP500Cancer [24] and dbSNP in NCBI. For PCR-RFLP primers, the natural and mutagenic primers that are available for rs12947788 and rs6503048 are also shown (Figures 3A2 and 3B2 respectively). For the mutagenic primer, the mutagenic (artificial) nucleotide is marked with red color in the forward (F) primer sequence. Currently, TaqMan probes may not be always available as public resources and sometimes there are no suitable TaqMan probes for some SNPs. In this case, PCR-RFLP using natural and mutagenic primers coupled with their corresponding restriction enzymes can solve this problem. Moreover, the PCR-RFLP method is more cost-effective than TaqMan probes using real-time PCR.

The primer sequences, position, length, GC no., GC%, Tm value, and Tm-difference, and product length after PCR-RFLP genotyping are provided. The restriction enzyme list for both the sense and antisense strands are shown in Figure 3B2 but omitted in Figure 3A2. The flanking sequences, suppliers, and NEB price for restriction enzymes for the target SNPs are provided as well (shown in Figures 3A2 and 3A3 but omitted in Figures 3B2 and 3B3).

## Conclusions

SNP-RFLPing 2 has significant advantages over SNP-RFLPing 1, because the new features complement many of the new comprehensive fields that are associated with modern SNP-related research and because the on-line retrieval systems avoid the need for updates from most databases. In this paper, we describe an updated web-based interface and a java-based program, SNP-RFLPing 2, which is able to provide comprehensive PCR-RFLP information, including RFLP enzymes and their appropriate primer set so that SNP genotyping can be carried out. SNP-RFLPing 2 can also be applied to many PCR-RFLP-based fields, such as the characterization of microorganisms [43,44], food authentication [45,46], and avian gender determination [47]. On-line example inputs have been used to demonstrate each of the main functions of SNP-RFLPing 2 that are described in the user manual, which is downloadable from the homepage URL.

## Availability and requirements

Project home page: http://bio.kuas.edu.tw/snp-rflping2/

Operating system(s): Operating systems with web browser.

Programming language: Java

Other requirements: Java 1.5.0 (or later)

License: none for academic users. For any restrictions regarding the use by non-academics please contact the corresponding author.

## List of abbreviations

SNP: single nucleotide polymorphism; RFLP: restriction fragment length polymorphism; NCBI: National Center for Biotechnology Information; HUGO: Human Genome Organization; REBASE: The Restriction Enzyme Database; PolymiRTS: Polymorphism in the microRNA Target Site; GO: gene ontology; CGAP: The Cancer Genome Anatomy Project.

## Authors' contributions

Conceived and designed the software: LYC and HWC. System and software development: YHC and CHY. Wrote the paper: HWC and CHY. All authors read and approved the final manuscript.

## Supplementary Material

Additional file 1 **The web application for SNP-RFLPing 2 is compressed into a file for downloading**. The web application has been compressed into a file named "snp-rflping2.rar" in order to ensure that the software will continue to be available. A web server Tomcat 6.0 (http://tomcat.apache.org/download-60.cgi) must be downloaded and set up before running the program. Click here for file

## References

[B1] Sober S Org E Kepp K Juhanson P Eyheramendy S Gieger C Lichtner P Klopp N Veldre G Viigimaa M Targeting 160 candidate genes for blood pressure regulation with a genome-wide genotyping array PLoS One 2009 4 6 e6034 10.1371/journal.pone.0006034 19562039 PMC2699027

[B2] Griffin TJ Smith LM Single-nucleotide polymorphism analysis by MALDI-TOF mass spectrometry Trends in biotechnology 2000 18 2 77 84 10.1016/S0167-7799(99)01401-8 10652512

[B3] Borgiani P Ciccacci C Forte V Sirianni E Novelli L Bramanti P Novelli G CYP4F2 genetic variant (rs2108622) significantly contributes to warfarin dosing variability in the Italian population Pharmacogenomics 2009 10 2 261 266 10.2217/14622416.10.2.261 19207028

[B4] Zhang J Wheeler DA Yakub I Wei S Sood R Rowe W Liu PP Gibbs RA Buetow KH SNPdetector: a software tool for sensitive and accurate SNP detection PLoS Comput Biol 2005 1 5 e53 10.1371/journal.pcbi.0010053 16261194 PMC1274293

[B5] Kwok PY SNP genotyping with fluorescence polarization detection Hum Mutat 2002 19 4 315 323 10.1002/humu.10058 11933186

[B6] Olivier M The Invader assay for SNP genotyping Mutat Res 2005 573 1-2 103 110 15829241 10.1016/j.mrfmmm.2004.08.016 PMC2771639

[B7] Ahmadian A Ehn M Hober S Pyrosequencing: history, biochemistry and future Clin Chim Acta 2006 363 1-2 83 94 10.1016/j.cccn.2005.04.038 16165119

[B8] Benusiglio PR Pharoah PD Smith PL Lesueur F Conroy D Luben RN Dew G Jordan C Dunning A Easton DF HapMap-based study of the 17q21 ERBB2 amplicon in susceptibility to breast cancer British journal of cancer 2006 95 12 1689 1695 10.1038/sj.bjc.6603473 17117180 PMC2360759

[B9] Carr EJ Clatworthy MR Lowe CE Todd JA Wong A Vyse TJ Kamesh L Watts RA Lyons PA Smith KG Contrasting genetic association of IL2RA with SLE and ANCA-associated vasculitis BMC Med Genet 2009 10 22 10.1186/1471-2350-10-22 19265545 PMC2662820

[B10] Kocsis AK Kiss ZF Tiszlavicz L Tiszlavicz Z Mandi Y Potential role of human beta-defensin 1 in Helicobacter pylori-induced gastritis Scand J Gastroenterol 2009 44 3 289 295 10.1080/00365520802530879 18991164

[B11] Yoshiya G Takahata T Hanada N Suzuki K Ishiguro A Saito M Sasaki M Fukuda S Influence of cancer-related gene polymorphisms on clinicopathological features in colorectal cancer J Gastroenterol Hepatol 2008 23 6 948 953 10.1111/j.1440-1746.2008.05307.x 18205772

[B12] Demirci FY Manzi S Ramsey-Goldman R Kenney M Shaw PS Dunlop-Thomas CM Kao AH Rhew EY Bontempo F Kammerer C Association study of Toll-like receptor 5 (TLR5) and Toll-like receptor 9 (TLR9) polymorphisms in systemic lupus erythematosus J Rheumatol 2007 34 8 1708 1711 17516623

[B13] Lin GT Tseng HF Yang CH Hou MF Chuang LY Tai HT Tai MH Cheng YH Wen CH Liu CS Combinational polymorphisms of seven CXCL12-related genes are protective against breast cancer in Taiwan OMICS 2009 13 2 165 172 10.1089/omi.2008.0050 19196101

[B14] Yen CY Liu SY Chen CH Tseng HF Chuang LY Yang CH Lin YC Wen CH Chiang WF Ho CH Combinational polymorphisms of four DNA repair genes XRCC1, XRCC2, XRCC3, and XRCC4 and their association with oral cancer in Taiwan J Oral Pathol Med 2008 37 5 271 277 18410587 10.1111/j.1600-0714.2007.00608.x

[B15] Niu T Hu Z SNPicker: a graphical tool for primer picking in designing mutagenic endonuclease restriction assays Bioinformatics 2004 20 17 3263 3265 10.1093/bioinformatics/bth360 15201186

[B16] Vincze T Posfai J Roberts RJ NEBcutter: A program to cleave DNA with restriction enzymes Nucleic Acids Res 2003 31 13 3688 3691 10.1093/nar/gkg526 12824395 PMC168933

[B17] Gardner SN Wagner MC Software for optimization of SNP and PCR-RFLP genotyping to discriminate many genomes with the fewest assays BMC Genomics 2005 6 1 73 10.1186/1471-2164-6-73 15904493 PMC1156889

[B18] Ke X Collins A Ye S PIRA PCR designer for restriction analysis of single nucleotide polymorphisms Bioinformatics 2001 17 9 838 839 10.1093/bioinformatics/17.9.838 11590100

[B19] Zhang R Zhu Z Zhu H Nguyen T Yao F Xia K Liang D Liu C SNP Cutter: a comprehensive tool for SNP PCR-RFLP assay design Nucleic Acids Res 2005 33 Web Server W489 492 10.1093/nar/gki358 15980518 PMC1160119

[B20] Xu H Gregory SG Hauser ER Stenger JE Pericak-Vance MA Vance JM Zuchner S Hauser MA SNPselector: a web tool for selecting SNPs for genetic association studies Bioinformatics 2005 21 22 4181 4186 10.1093/bioinformatics/bti682 16179360 PMC1361283

[B21] Thiel T Kota R Grosse I Stein N Graner A SNP2CAPS: a SNP and INDEL analysis tool for CAPS marker development Nucleic Acids Res 2004 32 1 e5 10.1093/nar/gnh006 14704362 PMC373308

[B22] Bikandi J San Millan R Rementeria A Garaizar J In silico analysis of complete bacterial genomes: PCR, AFLP-PCR and endonuclease restriction Bioinformatics 2004 20 5 798 799 10.1093/bioinformatics/btg491 14752001

[B23] Chang HW Yang CH Chang PL Cheng YH Chuang LY SNP-RFLPing: restriction enzyme mining for SNPs in genomes BMC Genomics 2006 7 30 10.1186/1471-2164-7-30 16503968 PMC1386656

[B24] Packer BR Yeager M Burdett L Welch R Beerman M Qi L Sicotte H Staats B Acharya M Crenshaw A SNP500Cancer: a public resource for sequence validation, assay development, and frequency analysis for genetic variation in candidate genes Nucleic Acids Res 2006 34 Database D617 621 10.1093/nar/gkj151 16381944 PMC1347513

[B25] Field HI Scollen SA Luccarini C Baynes C Morrison J Dunning AM Easton DF Pharoah PD Seq4SNPs: new software for retrieval of multiple, accurately annotated DNA sequences ready formatted for SNP assay design BMC Bioinformatics 2009 10 1 180 10.1186/1471-2105-10-180 19523221 PMC2711078

[B26] Yang CH Cheng YH Chuang LY Chang HW SNP-Flankplus: SNP ID-centric retrieval for SNP flanking sequences Bioinformation 2008 3 4 147 149 19238236 10.6026/97320630003147 PMC2637961

[B27] Pico AR Smirnov IV Chang JS Yeh RF Wiemels JL Wiencke JK Tihan T Conklin BR Wrensch M SNPLogic: an interactive single nucleotide polymorphism selection, annotation, and prioritization system Nucleic Acids Res 2009 37 Database D803 809 10.1093/nar/gkn756 18984625 PMC2686434

[B28] Yang CH Chuang LY Cheng YH Wen CH Chang PL Chang HW SNP ID-info: SNP ID searching and visualization platform OMICS 2008 12 3 217 226 10.1089/omi.2008.0026 18582176

[B29] Sherry ST Ward MH Kholodov M Baker J Phan L Smigielski EM Sirotkin K dbSNP: the NCBI database of genetic variation Nucleic Acids Res 2001 29 1 308 311 10.1093/nar/29.1.308 11125122 PMC29783

[B30] Roberts RJ Vincze T Posfai J Macelis D REBASE--enzymes and genes for DNA restriction and modification Nucleic Acids Res 2007 35 Database D269 270 10.1093/nar/gkl891 17202163 PMC1899104

[B31] Thorisson GA Smith AV Krishnan L Stein LD The International HapMap Project Web site Genome Res 2005 15 11 1592 1593 10.1101/gr.4413105 16251469 PMC1310647

[B32] Bao L Zhou M Wu L Lu L Goldowitz D Williams RW Cui Y PolymiRTS Database: linking polymorphisms in microRNA target sites with complex traits Nucleic Acids Res 2007 35 Database D51 54 10.1093/nar/gkl797 17099235 PMC1669716

[B33] Barrell D Dimmer E Huntley RP Binns D O'Donovan C Apweiler R The GOA database in 2009--an integrated Gene Ontology Annotation resource Nucleic Acids Res 2009 37 Database D396 403 10.1093/nar/gkn803 18957448 PMC2686469

[B34] Chang HW Chuang LY Cheng YH Hung YC Wen CH Gu DL Yang CH Prim-SNPing: a primer designer for cost-effective SNP genotyping Biotechniques 2009 46 6 421 431 10.2144/000113092 19480636

[B35] Benson DA Karsch-Mizrachi I Lipman DJ Ostell J Sayers EW GenBank Nucleic Acids Res 2009 37 Database D26 31 10.1093/nar/gkn723 18940867 PMC2686462

[B36] Chang HW Chuang LY Cheng YH Ho CH Wen CH Yang CH Seq-SNPing: multiple-alignment tool for SNP discovery, SNP ID identification, and RFLP genotyping OMICS 2009 13 3 253 260 10.1089/omi.2008.0058 19514837

[B37] A haplotype map of the human genome Nature 2005 437 7063 1299 1320 10.1038/nature04226 16255080 PMC1880871

[B38] Birney E Stamatoyannopoulos JA Dutta A Guigo R Gingeras TR Margulies EH Weng Z Snyder M Dermitzakis ET Thurman RE Identification and analysis of functional elements in 1% of the human genome by the ENCODE pilot project Nature 2007 447 7146 799 816 10.1038/nature05874 17571346 PMC2212820

[B39] Ambros V The functions of animal microRNAs Nature 2004 431 7006 350 355 10.1038/nature02871 15372042

[B40] Bartel DP MicroRNAs: genomics, biogenesis, mechanism, and function Cell 2004 116 2 281 297 10.1016/S0092-8674(04)00045-5 14744438

[B41] Migliore C Giordano S MiRNAs as new master players Cell Cycle 2009 8 14 2185 2186 19617713 10.4161/cc.8.14.9113

[B42] Mishra PJ Bertino JR MicroRNA polymorphisms: the future of pharmacogenomics, molecular epidemiology and individualized medicine Pharmacogenomics 2009 10 3 399 416 10.2217/14622416.10.3.399 19290790 PMC2705205

[B43] Ubeda JF Fernandez-Gonzalez M Briones AI Application of PCR-TTGE and PCR-RFLP for intraspecific and interspecific characterization of the genus Saccharomyces using actin gene (ACT1) primers Curr Microbiol 2009 58 1 58 63 10.1007/s00284-008-9283-9 18941833

[B44] Tasleem Raza S Husain N Kumar A Screening for hemophilia A carriers: utility of PCR-RFLP--based polymorphism analysis Clin Appl Thromb Hemost 2009 15 1 78 83 10.1177/1076029607305105 19150994

[B45] Rojas M Gonzalez I Fajardo V Martin I Hernandez PE Garcia T Martin R Polymerase chain reaction-restriction fragment length polymorphism authentication of raw meats from game birds J AOAC Int 2008 91 6 1416 1422 19202803

[B46] Espineira M Gonzalez-Lavin N Vieites JM Santaclara FJ Authentication of anglerfish species (Lophius spp) by means of polymerase chain reaction-restriction fragment length polymorphism (PCR-RFLP) and forensically informative nucleotide sequencing (FINS) methodologies J Agric Food Chem 2008 56 22 10594 10599 10.1021/jf801728q 18975961

[B47] Costantini V Guaricci AC Laricchiuta P Rausa F Lacalandra GM DNA sexing in Humboldt Penguins (Spheniscus humboldti) from feather samples Anim Reprod Sci 2008 106 1-2 162 167 10.1016/j.anireprosci.2007.12.013 18258392

