# Supplementary material for: SNP-RFLPing 2: an updated and integrated PCR-RFLP tool for SNP genotyping
Source: BMC Bioinformatics. 2010 Apr 8;11:173. doi: 10.1186/1471-2105-11-173 (PMC2858040; doi:10.1186/1471-2105-11-173)
Supplement: Additional file 1 — The web application for SNP-RFLPing 2 is compressed into a file for downloading. The web application has been compressed into a file named "snp-rflping2.rar" in order to ensure that the software will continue to be available. A web server Tomcat 6.0 (http://tomcat.apache.org/download-60.cgi) must be downloaded and set up before running the program. [file 1471-2105-11-173-S1.PDF]

SNP-RFLPing 2

an updated and integrated PCR-RFLP tool for SNP genotyping

User Manual

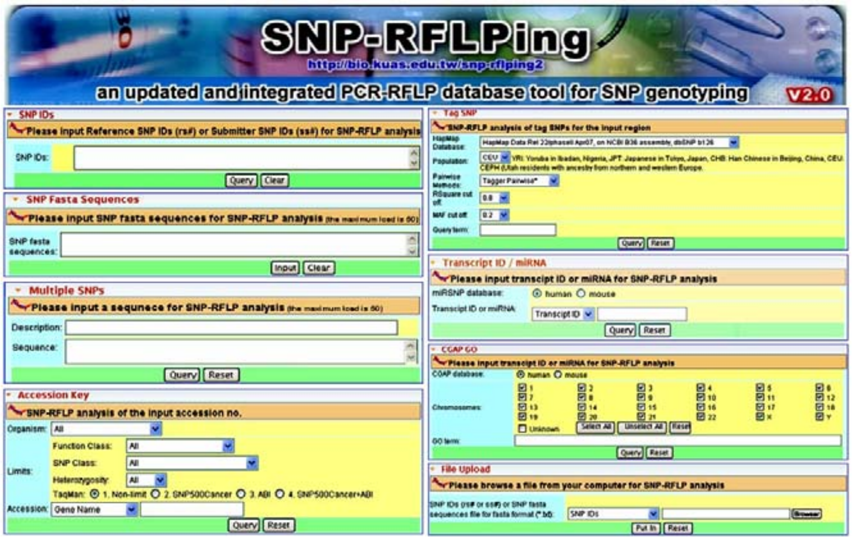

Contact:

changhw@kmu.edu.tw

yuhuei.cheng@gmail.com

chuang@isu.edu.tw

chyang@cc.kuas.edu.tw

Updated: 2009/11/03

Table of content

1. Introduction p.2
1.1 Purpose p.2
1.2 System workflow p.2
1.3 Function overview p.3
2. Function: SNP ID for input p.4
2.1 Overview p.4
2.2 Example: A complete operation using SNP ID to analyze the SNP-RFLPing p.4
2.2.1 Example (1): SNP IDs input p.4
2.2.2 Example (2): Query SNPs information p.10
2.2.3 Example (3): SNP-RFLP analysis p.11
2.2.4 Example (4): Natural primers information acquiring p.12
2.2.5 Example (5): Available restriction enzymes request p.13
2.2.6 Example (6): Further restriction enzyme information description p.14
2.2.7 Example (7): Mutagenic primer information acquiring p.17
2.2.8 Example (8): TaqMan information providing p.18
3. Function: SNP in fasta sequence for input p.20
3.1 Overview p.20
3.2 Example: Use SNP in fasta sequence format to analyze p.20
4. Function: Multiple SNPs within one sequence for input p.29
4.1 Overview p.29
4.2 Example: Multiple SNPs within one sequence input p.29
5. Function: GenBank accession no. for input p.33
5.1 Overview p.33
5.2 Example: Use HUGO gene name ‘TP53’ to analyze p.33
6. Function: TagSNP from HapMap p.35
6.1 Overview p.35
6.2 Example: Use HUGO gene name ‘BRCA2’ to analyze p.35
7. Function: Transcript ID/miRNA p.37
7.1 Overview p.37
7.2 Example: Use transcript ID and miRNA number to analyze p.37
8. Function: Gene Ontology-based annotation for SNPs p.40
8.1 Overview p.40
8.2 Example: Query Gene Ontology vocabulary term p.40
9. Function: File upload for input p.43
9.1 Overview p.43
9.2 Example: Upload interface for SNP IDs and SNP fasta sequences p.43
10. Appendix p.44
10.1 Definitions p.44
10.2 Related links p.44

1. Introduction

1.1 Background

PCR-restriction fragment length polymorphism (RFLP) assay is a cost-effective method for SNP genotyping and mutation detection, but the manual mining of restriction enzymes is challenging and cumbersome. Three years after we constructed SNP-RFLPing, a freely accessible analysis tool for restriction enzyme mining of SNPs, significant improvements over the 2006 version have been made in the latest version, SNP-RFLPing 2. The primary aim of SNP-RFLPing 2 is to provide a comprehensive PCR-RFLP information for SNPs in multiple fields, such as SNPs for multiple species, multiple kinds (di-, tri, tetra- and indel), gene-centric search, HapMap tagSNPs, gene ontology-based search, miRNAs, and SNP500Cancer. The RFLP restriction enzymes and their corresponding PCR primers of natural and mutagenic types for each SNP are simultaneously analyzed. All the prices of the RFLP restriction enzymes are provided for selection. Furthermore, the previously encountered updating problems for most SNP related databases are resolved by an online retrieval system. The user interfaces for the functional SNP analyses have been substantially improved and integrated. SNP-RFLPing 2 offers a new, user-friendly interface for RFLP genotyping in association studies and is freely available at http://bio.kuas.edu.tw/snp-rflping2/rflpUI.jsp.

1.2 System workflow

The workflow of SNP-RFLPing 2 was illustrated in Figure 1 (see next page). The system was divided into nine modules, including: 1) input module, 2) data retrieve module, 3) sequence process module, 4) SNP-RFLP module, 5) data query module, 6) remote database module, 7) primer design module, 8) output module, and 9) SNP-RFLP database module according to MVC (Model-View-Controller) architecture. Input and Output modules are served with the graphical user interface (GUI) to operate and analyze.

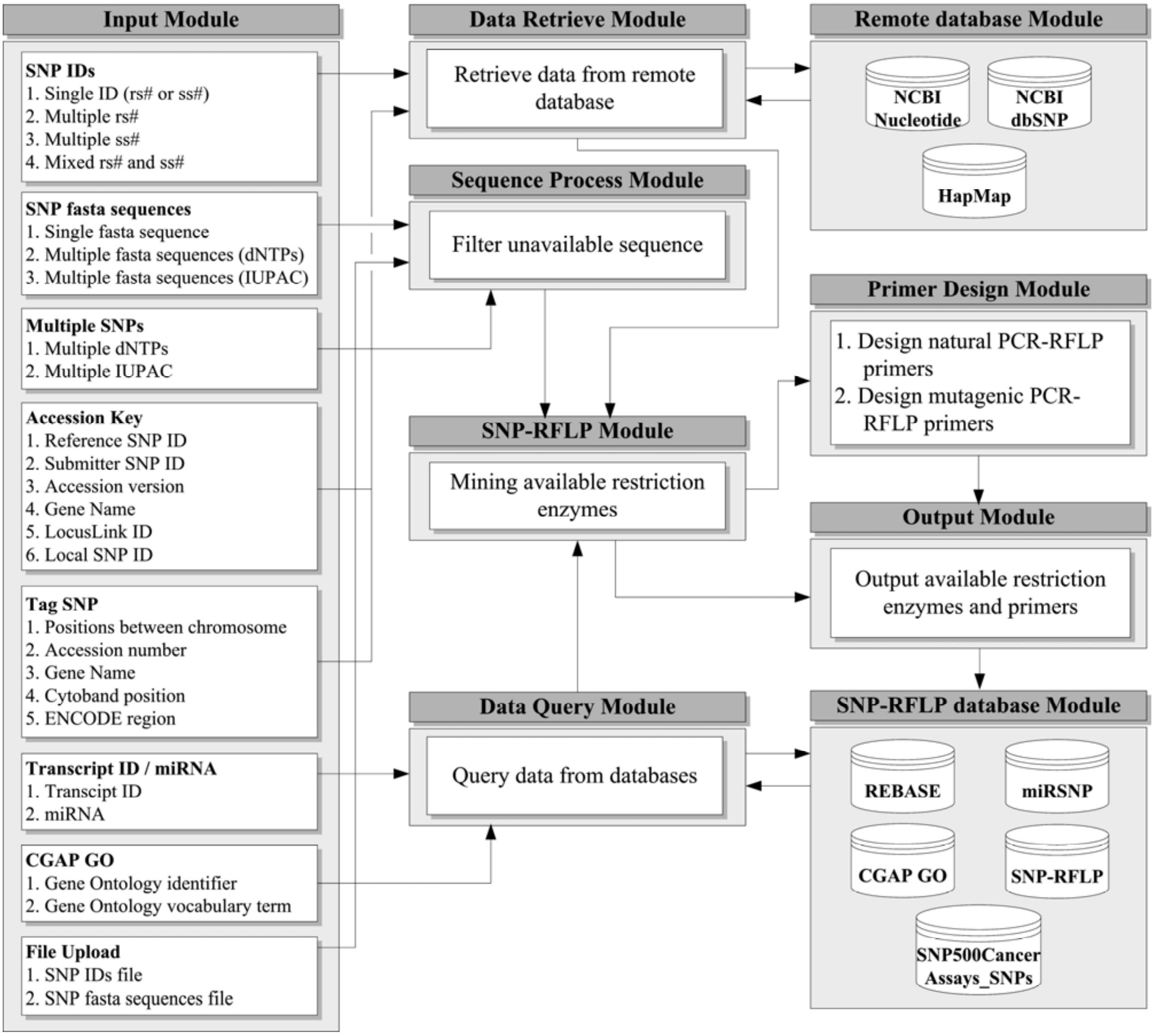

Fig. 1-1. System structure and flowchart for SNP-RFLPing 2.

1.3 Function overview

SNP-RFLPing 2 provides *eight major functions* to assist and analyze the RFLP restriction enzymes and its corresponding primers for SNPs in all SNP-related fields, including:

(1) SNP ID input

(2) SNP in fasta sequence input

(3) Multiple SNPs within one sequence

(4) GenBank accession

(5) TagSNP from HapMap

(6) Transcript ID/miRNA

(7) Gene Ontology-based annotation for SNPs

(8) File upload

2. Function: SNP ID input

2.1 Overview

SNP ID (rs# and ss#) is acceptable to query the SNP-RFLP information. Single ID, multiple rs#, multiple ss#, and mixed rs# and ss# are available for SNP ID input. For multiple SNP IDs, three symbols which are "comma", "space", and "line" can be used to separate SNPs. The maximal input SNP IDs is limited to fifty.

2.2 Example: A complete operation using SNP ID to analyze the SNP-RFLPing

2.2.1 Example (1): SNP IDs input

Fig. 2-1 and Fig. 2-2 show a single ID input for rs# and ss#, respectively.

Fig. 2-3, Fig. 2-4 and Fig. 2-5 show the multiple rs# input with the "comma" symbol, "space", and "line" (press the "Enter key" in keyboard) to separate, respectively.

Fig. 2-6, Fig. 2-7 and Fig. 2-8 show the multiple ss# input with comma symbol, space, and line to separate, respectively.

Fig. 2-9, Fig. 2-10 and Fig. 2-11 show the multiple rs# and ss# input with comma symbol, space, and line to separate, respectively.

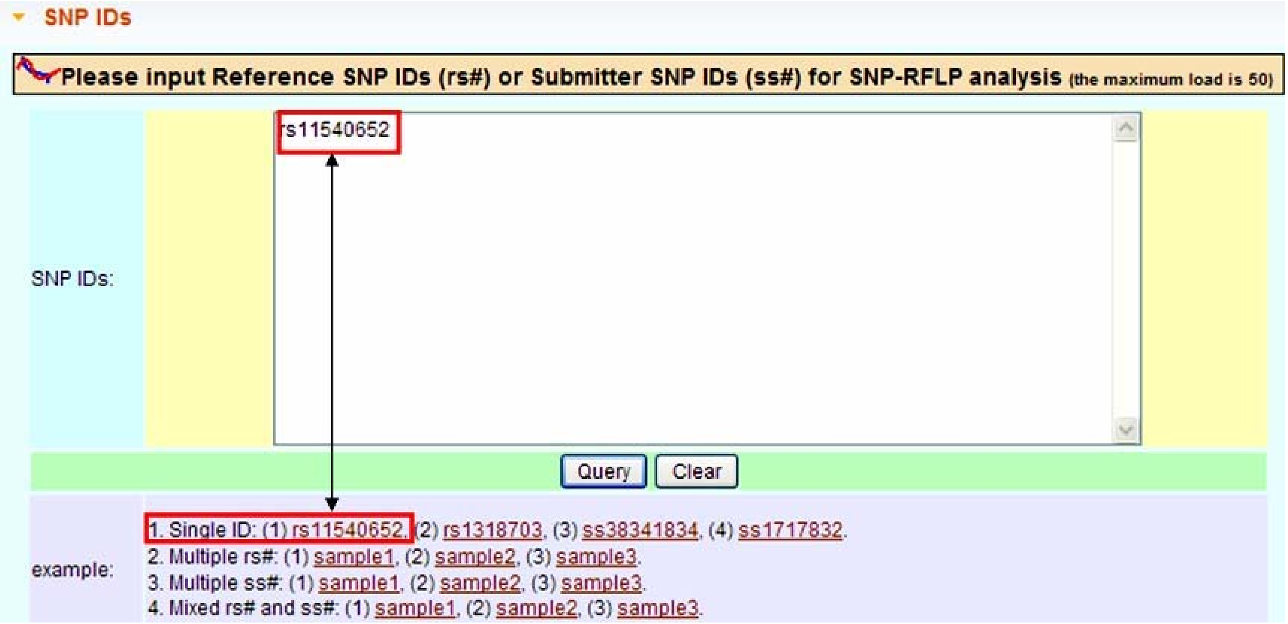

Fig. 2-1. Single ID input with rs#.

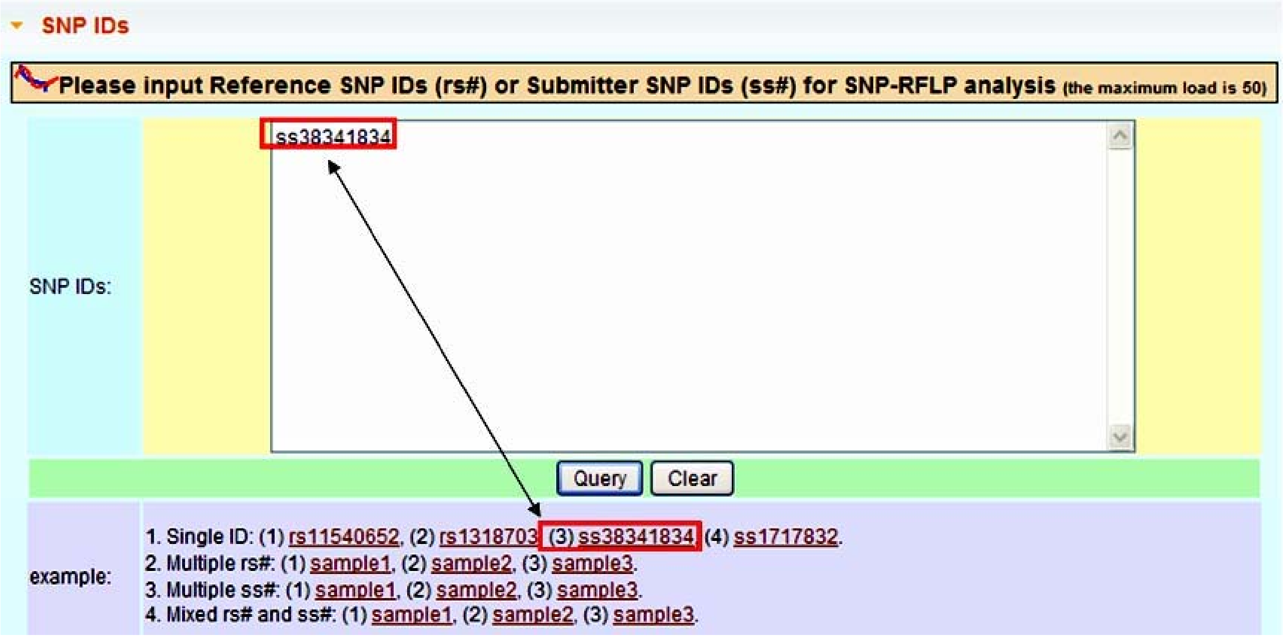

Fig. 2-2. Single ID input with ss#.

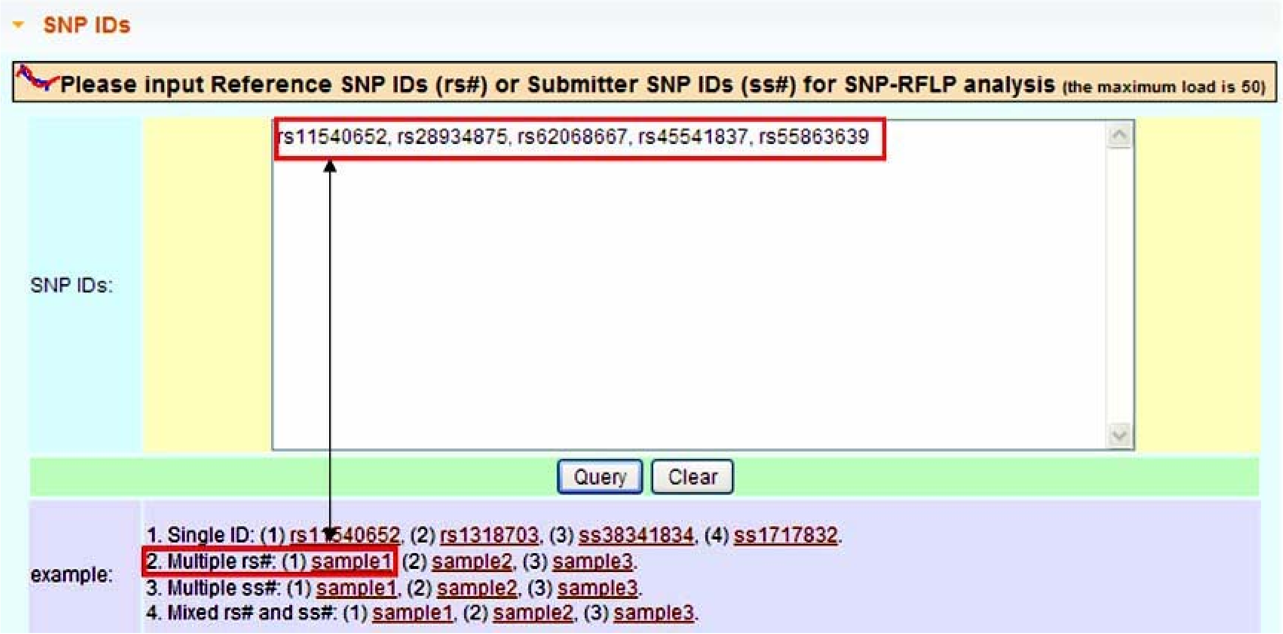

Fig. 2-3. Multiple rs# input with the comma symbols to separate.

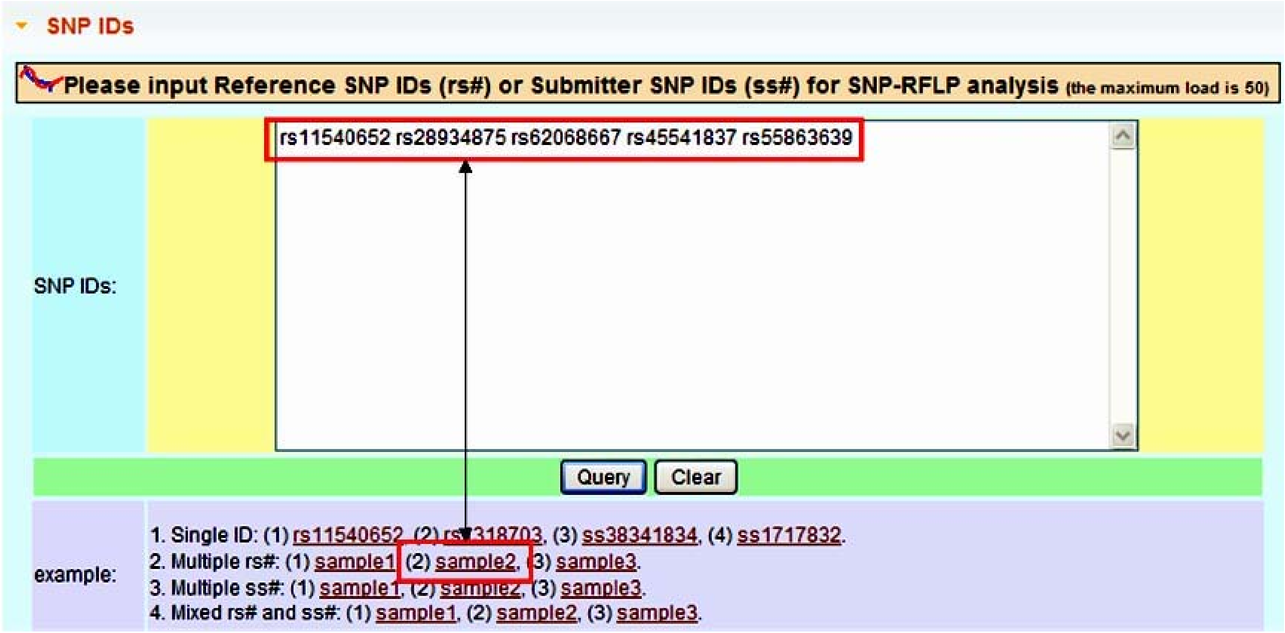

Fig. 2-4. Multiple rs# input with space to separate.

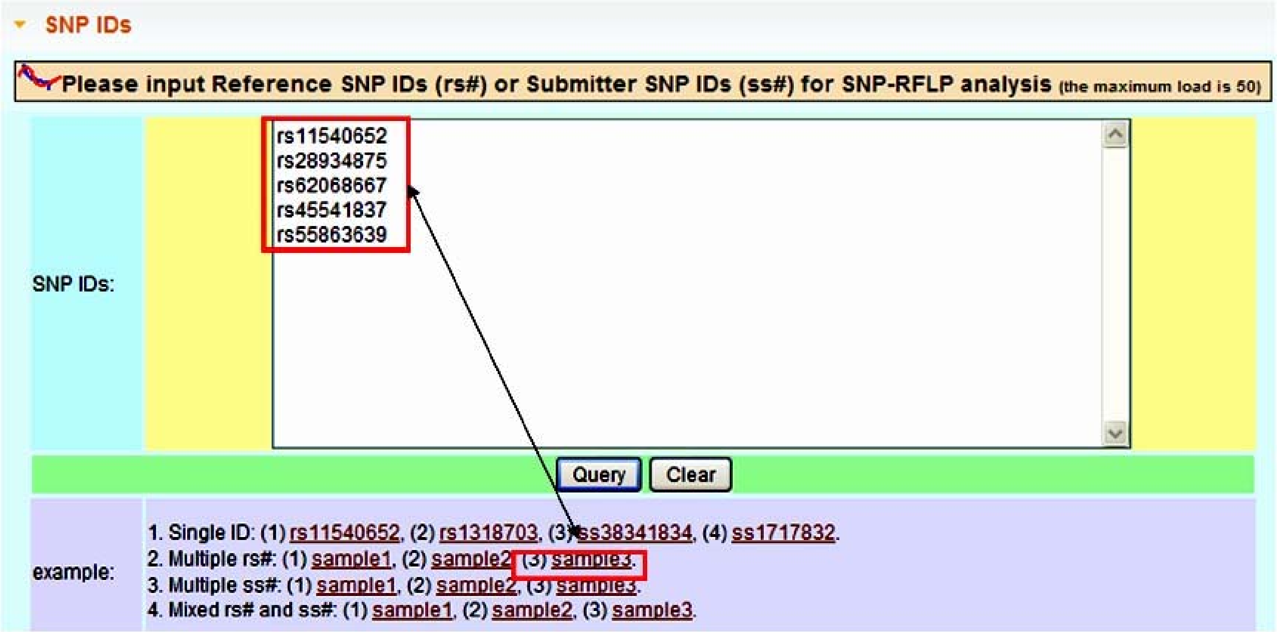

Fig. 2-5. Multiple rs# input with lines (press the enter key of keyboard) to separate.

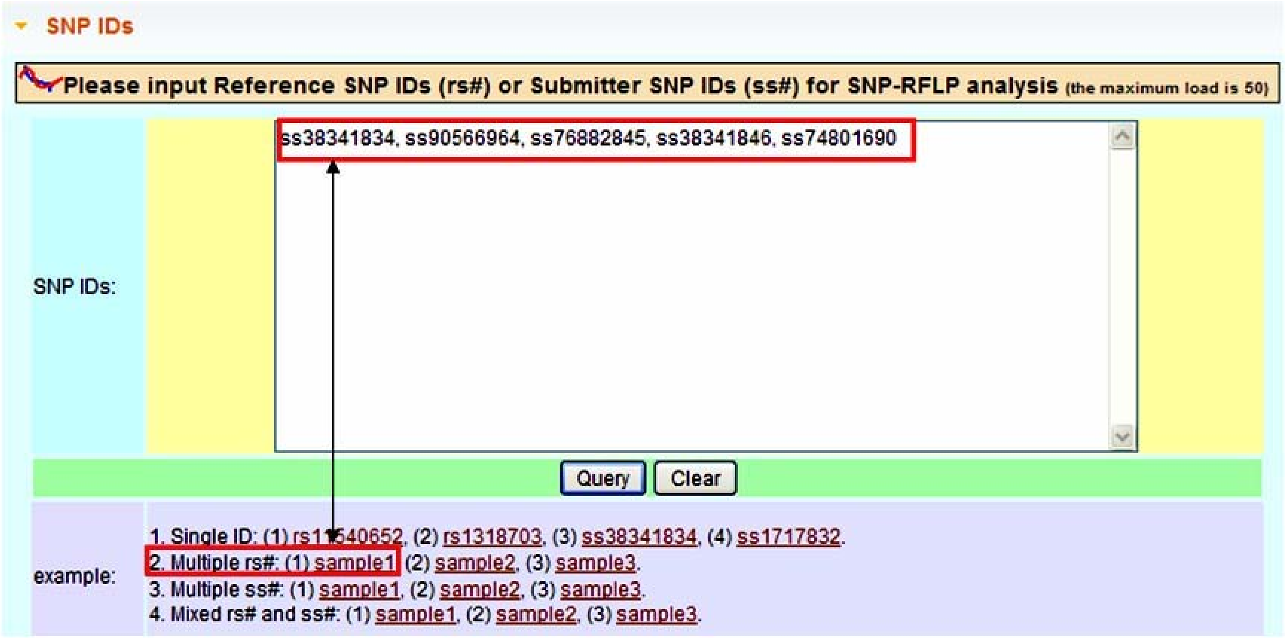

Fig. 2-6. Multiple ss# input with the comma symbols to separate.

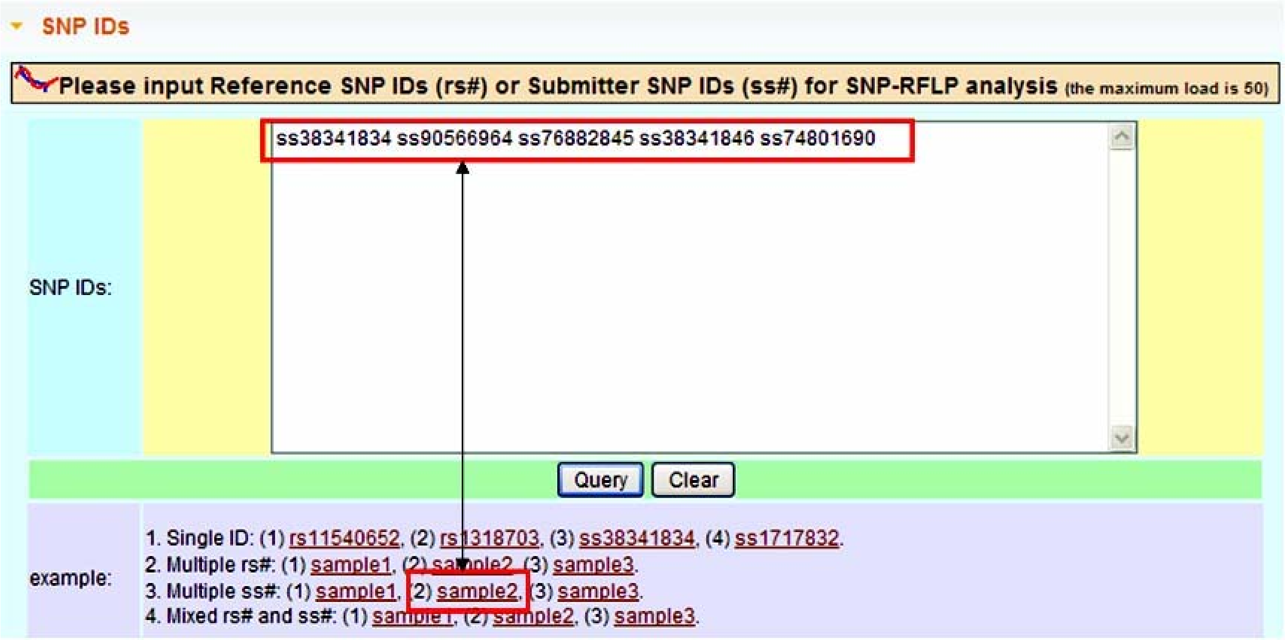

Fig. 2-7. Multiple ss# input with space to separate.

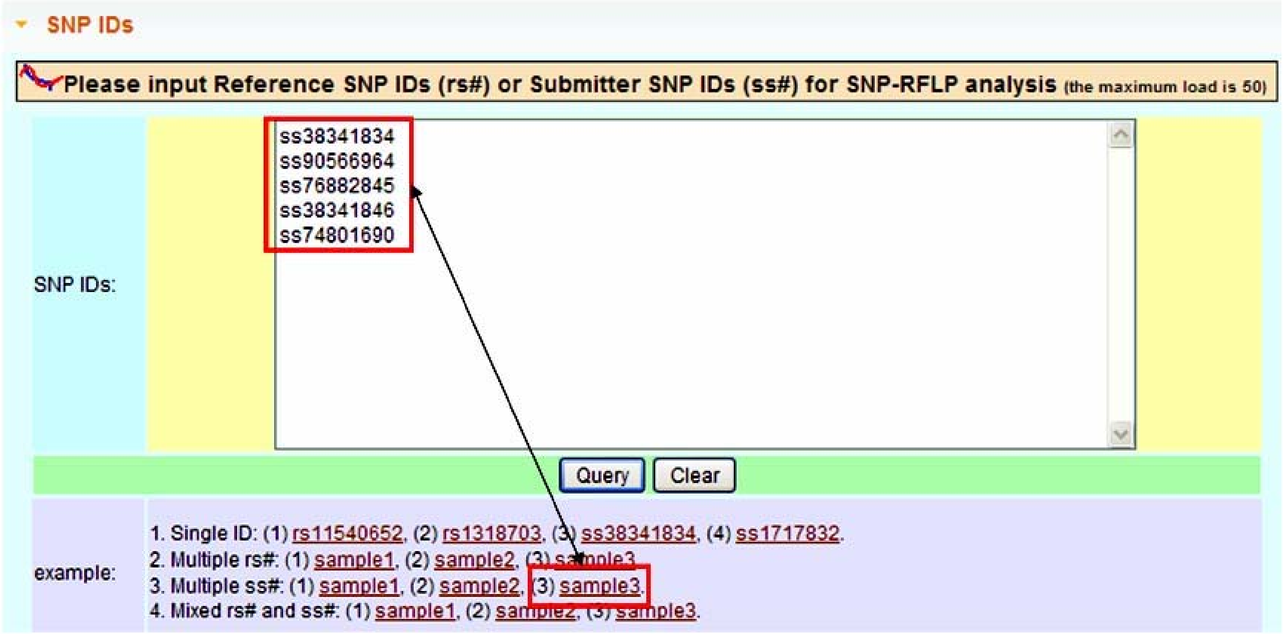

Fig. 2-8. Multiple ss# input with lines (press the enter key of keyboard) to separate.

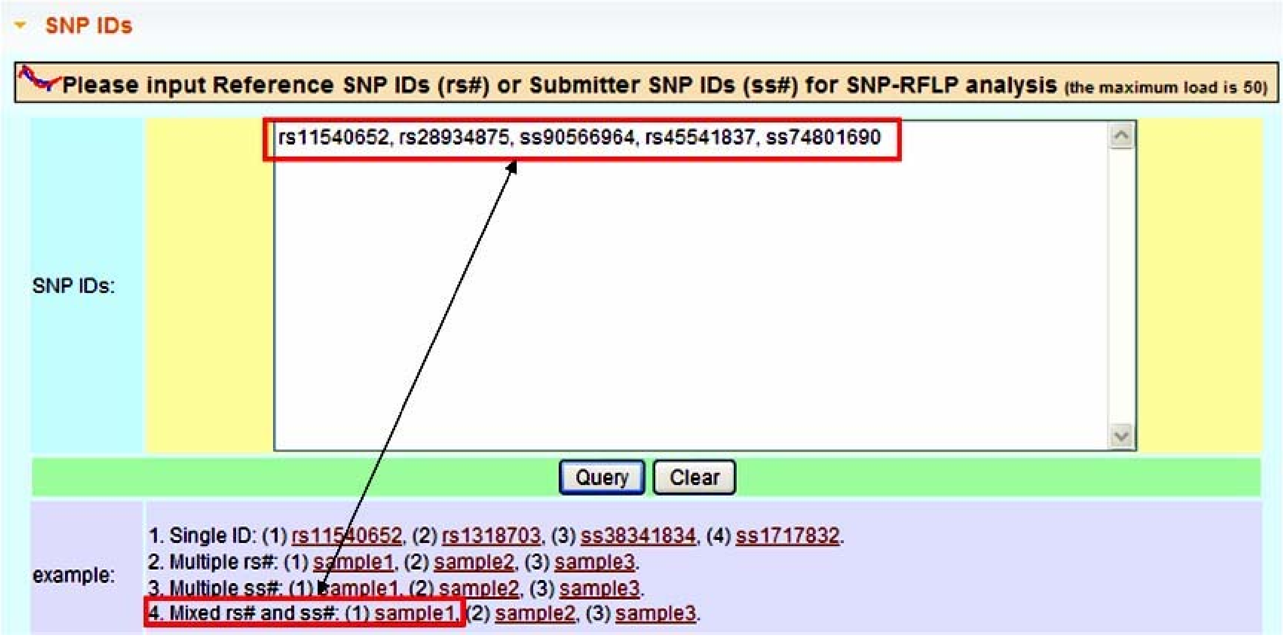

Fig. 2-9. Multiple rs# and ss# input with the comma symbols to separate.

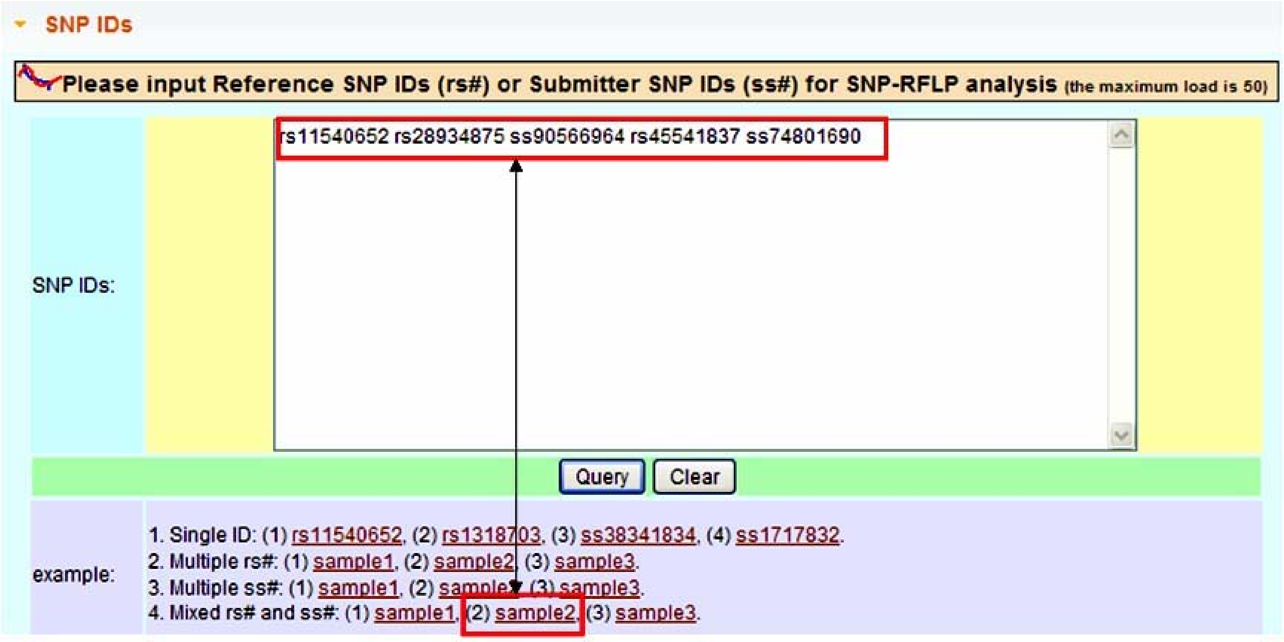

Fig. 2-10. Multiple rs# and ss# input with space to separate.

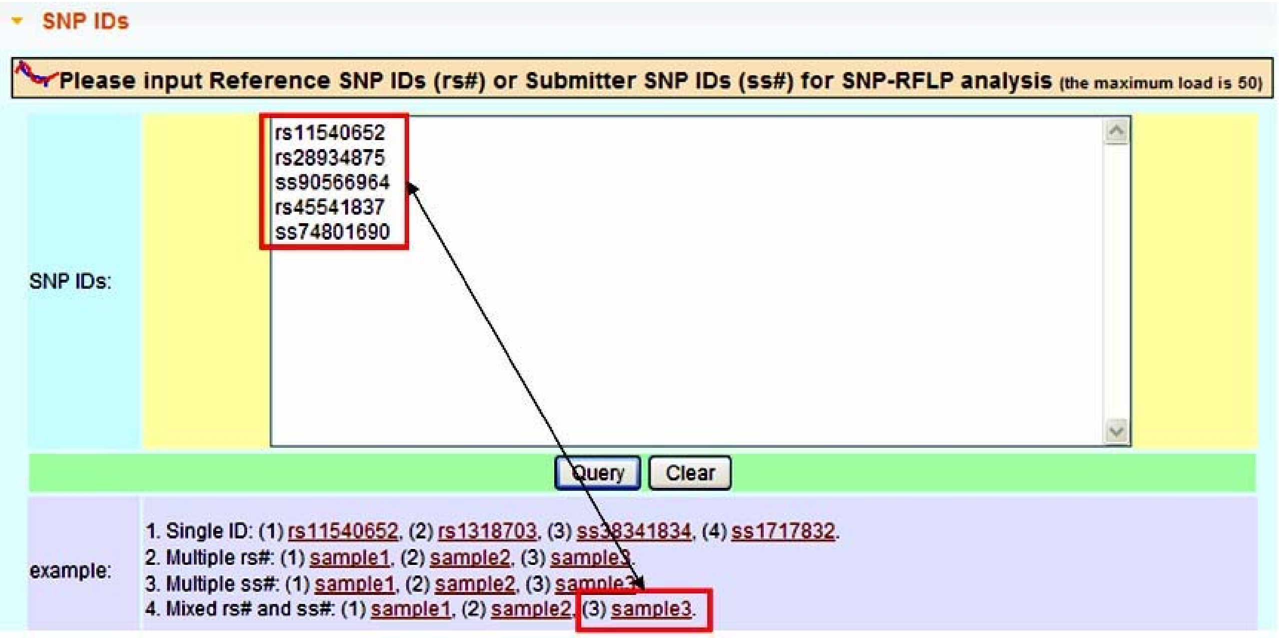

Fig. 2-11. Multiple rs# and ss# mixed input with lines (press the enter key of keyboard) to separate.

2.2.2 Example (2): Query SNPs information

After inputting SNP ID, users can click the “Query” button to query SNPs information. Users will see the waiting page (Fig. 2-12) for a moment and the result for SNP information will appear (Fig. 2-13). The result for SNP information includes: reference SNP ID (rs#), organism, short SNP flanking sequence (52 bp in total), chromosome information, clinical/LSDB associated, PubMed citing, SNP gene, sequence viewer, SNP 3D, OMIM, heterozygosity frequency, validated and genotype data, such as NCBI Entrez SNP. Furthermore, HGVS Names are provided for *Homo sapiens*.

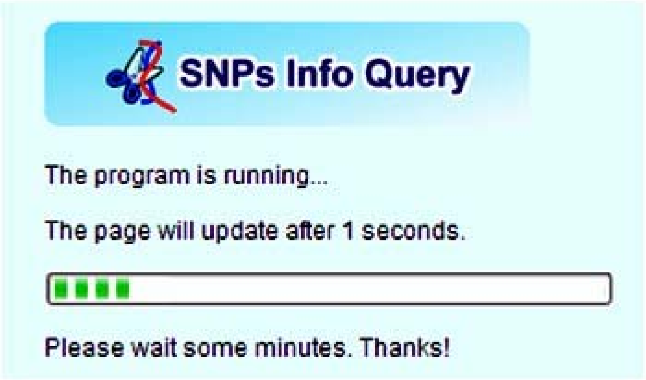

Fig. 2-12. Waiting for the SNP information query.

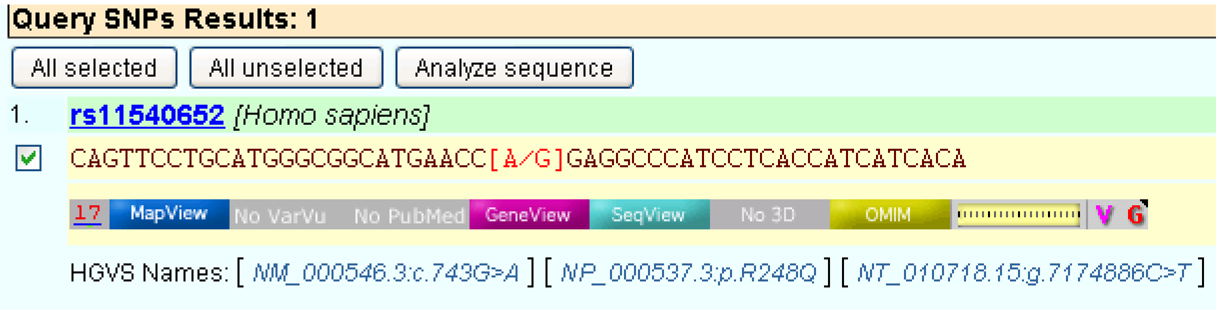

Fig. 2-13. The result for SNP information.

2.2.3 Example (3): SNP-RFLP analysis

Subsequently, user can check SNP for further analysis. In this example, a SNP rs11540652 is chosen to check (Fig. 2-13) and users can click the “Analyze sequence” button to execute SNP-RFLP function. When the “Analyze sequence” button is clicked, a waiting page will appear (Fig. 2-14).

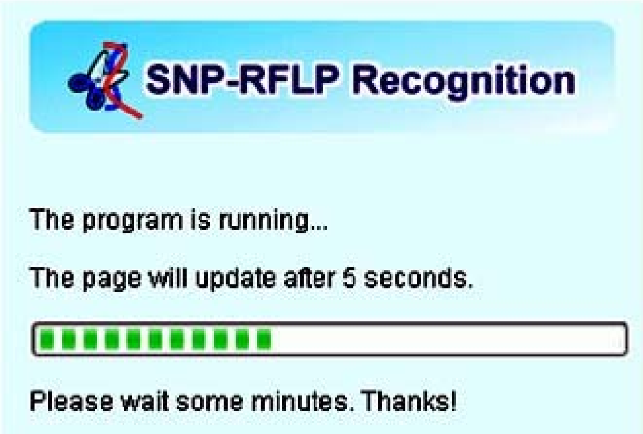

Fig. 2-14. Waiting for the SNP-RFLP recognition.

After the waiting page is disappeared, the result for SNP-RFLP recognition is appearing (Fig. 2-15). The result information of SNP-RFLP includes SNP ID (rs#), short SNP flanking sequence (52 bp in total), result for sense strand and anti-sense strand whether recognized by restriction enzymes or not, TaqMan, primer set, and enzymes information. The SNP ID is hyperlinked to NCBI Entrez SNP for further information. In this SNP (rs11540652), its TaqMan probe is not provided in both NCBI and SNP500Cancer and the natural primer set (*i.e.*, *the primers are the regular primers and the RFLP restriction enzymes are available*) is available.

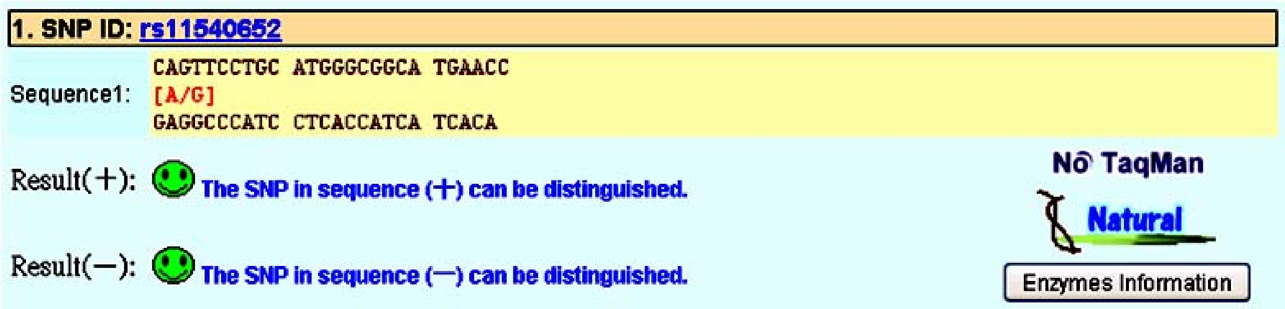

Fig. 2-15. The result for SNP-RFLP recognition. + is the sense strand and – is the antisense strand. Smile symbol means that the RFLP enzymes are available in the SNP-RFLPing 2 analysis.

2.2.4 Example (4): Natural primers information acquiring

Clicking the icon of "Natural", the natural primer set information is appearing (Fig. 2-16). The natural primer set information contains SNP ID, strand orientation, recognized allele, restriction enzymes, natural primer pair information, and natural primers visualization.

SNP ID: rs11540652

Strand orientation	Recognized allele	Restriction enzymes
+	A	BspNCI DrdII StyLTI
+	G	Asp748I Bba179I Bca77I Bco27I BetI BsaWI BsaZI BshMI BsiSI Bsp5I Bsp47I Bsp48I Bsp116I Bsp1591II Bst40I Bst1473I Bsu1192I BsuFI CboI CceI M.Csp68KIV FinII HapII Hin2I Hin5I HpaII HpyVIII HpyF43I MniII MnoI MspI Msp199I Pde137I Pme35I SecII SfaGUI Sth134I Sth302II Uba1128I Uba1141I Uba1267I Uba1338I Uba1355I Uba1439I UbaN10I
–	T	Alw26I BcoDI Bli49I Bli161I Bli576II Bli736I Bli5508I BsaI BscQII BsmAI Bso31I BsoMAI BspTNI BstMAI Cfr56I Eco31I Eco42I Eco51I Eco95I Eco97I Eco101I Eco120I Eco127I Eco129I Eco155I Eco156I Eco157I Eco162I Eco185I Eco191I Eco203I Eco204I Eco205I Eco217I Eco225I Eco233I Eco239I Eco240I Eco241I Eco246I Eco247I Eco263I EcoA4I Eco71KI EcoO44I PpaI Rle69I Sau12I SlbI Uba65I Uba84I Uba1316I Uba1343I VpaK57I VpaK57AI VpaKutHI
–	C	AcaIV Afl83II Asp742I AspTIII AvrBI Bal475I Bal3006I BanAI Bce71I Bco33I BecAII Bfi458I BhaII Bim19II BliI BluII Bme46I Bme74I Bme361I BmeU1594I Bpa36I BsaRI BscQI BseI Bse9I Bse126I BseQI BshI BshAI BshBI BshCI BshDI BshEI BshFI BsiAI BsiDI BsiHI BsnI Bsp23I Bsp44II Bsp137I Bsp211I Bsp226I Bsp881I Bsp1261I Bsp1593I Bsp2013I Bsp2362I Bsp2500I BspANI BspBDG2I BspBRI BspBSE18I BspBake1I BspCHE15I BspGHA1I BspH106II BspKI BspLRI BspRI BssCI BstCI BstJI Bsu1076I Bsu1114I BsuRI BteI ClmI CltI Csp2I M.Csp68KV M.CviAIV CviJI CviKI CviKI-1 CviLI CviMI CviNI CviOI DsaII EsaBC4I EsaWC1I FinSI FnuDI M.H2I HaeI HaeIII HhgI Hpy166III Hpy178VII HpyF10V HpyF26II HpyF29I HpyF33II HpyF42I HpyF46V HpyF49IV HpyF53I HpyF57I HpyF63I HpyF69II HpyF72I HpyF73III MchAII MfoAI MniI MnlI MnnII MthTI NgoAII NgoCII M.NgoGII NgoNII NgoPII NgoSII NgoTII NlaI NspLKI OchI PaiI PalI Pde133I PflKI PhoI PlaI Ple214I PpuI Pru2I Psb9879I Psp29I SaqI SbvI SfaI SplIII SuaI SulI Tsp132I Tsp266I Tsp273II Tsp281I Tsp560I TspZNI TteAI TtnI Uba9I Uba54I Uba61I Uba1097I Uba1140I Uba1146I Uba1147I Uba1150I Uba1152I Uba1153I Uba1155I Uba1169I Uba1174I Uba1175I Uba1176I Uba1178I Uba1179I Uba1207I Uba1208I Uba1209I Uba1210I Uba1214I Uba1223I Uba1228I Uba1230I Uba1231I Uba1235I Uba1288I Uba1292I Uba1293I Uba1319I Uba1322I Uba1336I Uba1377I Uba1388I Uba1392I Uba1395I Uba1408I Uba1418I Uba1422I Uba1429I Uba1449I Uba1450I UbaN2I UbaN8I Uth549I Uth555I Uth557I Van91II VhaI Vha1168I VniI

primer pair	position	length (bp)	GC no.	GC %	tm (°C)	tm-diff (°C)	product length (bp)
F: CAAGGCGCACTGGCCTC	388-404	17	12	70.59	58	4	113
							168
R: GGGGATGTGATGAGAGG	651-667	17	10	58.82	54		280

```
000001 GTTTAACTAT TGCACAGTTG AAAAAACTGA AGCTTACAGA GGCTAAGGGC CTCCCCTGCT
000061 TGGCTGGGCG CAGTGGCTCA TGCCTGTAAT CCCAGCACTT TGGGAGGCCA AGGCAGGCGG
000121 ATCACGAGGT TGGGAGATCG AGACCATCCT GGCTAACGGT GAAACCCCGT CTCTACTGAA
000181 AAATACAAAA AAAAATTAGC CGGGCGTGGT GCTGGGCACC TGTAGTCCCA GCTACTCGGG
000241 AGGCTGAGGA AGGAGAATGG CGTGAACCTG GGCGGTGGAG CTTGCAGTGA GCTGAGATCA
000301 CGCCACTGCA CTCCAGCCTG GGCGACAGAG CGAGATTCCA TCTCAAAAAA AAAAAAAAAA
000361 GGCCTCCCCT GCTTGCCACA GGTCTCCCCA AGGCGCACTG GCCTCATCTT GGGCCTGTGT
000421 TATCTCCTAG GTTGGCTC[illegible]G ACTGTACCAC CATCCACTAC AACTACATGT GTAACAGTTC
000481 CTGCATGGGC GGCATGAACC RGAGGCCCAT CCTCACCATC ATCACACTGG AAGACTCCAG
000541 GTCAGGAGCC ACTTGCCACC CTGCACACTG GCCTGCTGTG CCCCAGCCTC TGCTTGCCTC
000601 TGACCCCTGG GCCCACCTCT TACCGATTTC TTCCATACTA CTACCCATCC ACCTCTCATC
000661 ACATCCCCGG CGGGGAATCT CCTTACTGCT CCCACTCAGT TTTCTTTTCT CTGGCTTTGG
000721 GACCTCTTAA CCTGTGGCTT CTCCTCCACC TACCTGGAGC TGGAGCTTAG GCTCCAGAAA
000781 GGACAAGGGT GGTTGGGAGT AGATGGAGCC TGGTTTTTTA AATGGGACAG GTAGGACCTG
000841 ATTTCCTTAC TGCCTCTTGC TTCTCTTTTC CTATCCTGAG TAGTGGTAAT CTACTGGGAC
000901 GGAACAGCTT TGAGGTGCGT GTTTGTGCCT GTCCTGGGAG AGACCGGCGC ACAGAGGAAG
000961 AGAATCTCCG CAAGAAAGGG GAGCCTCACC ACGAGCTGCC C
```

Fig. 2-16. The restriction enzymes and natural primer set information for rs11540652. Both forward and reverse primers are underlined with red color and the SNP is indicated by an arrow line. + is the sense strand and – is the antisense strand.

2.2.5 Example (5): Available restriction enzymes request

In order to further get full enzymes information, users can click the “Enzymes Information” button. Fig. 2-17 shows only the available “sense” strand restriction enzymes information. When the checkbox for anti-sense strand is checked, the restriction enzymes information for both the available “sense” and “anti-sense” strands are shown in Fig. 2-18.

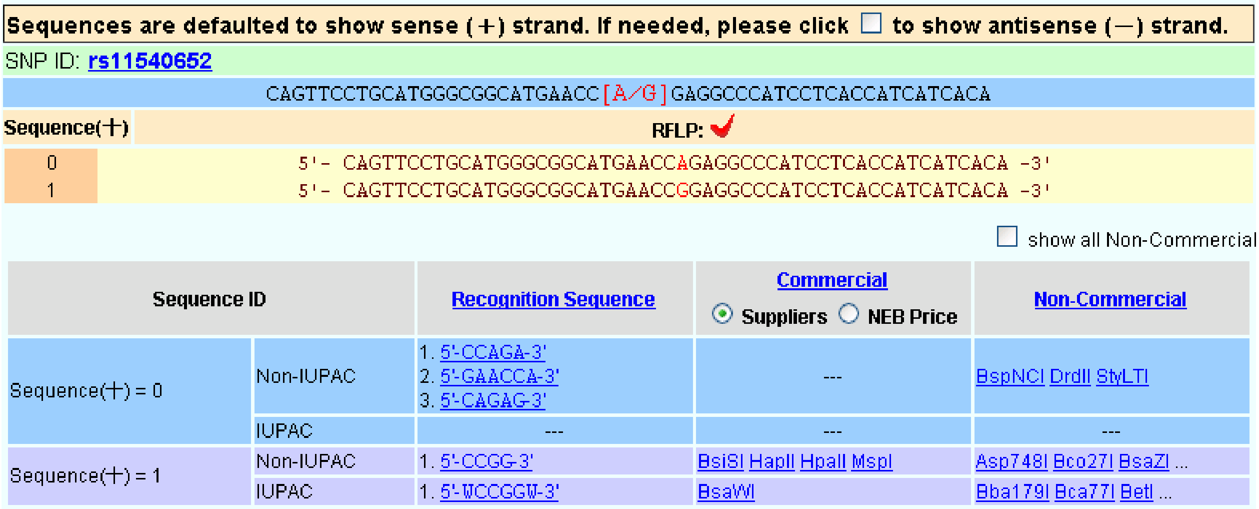

Fig. 2-17. The available sense strand restriction enzymes information for rs11540652. + is the sense strand and – is the antisense strand. 0 and 1 indicate the alternative nucleotide for the same SNP.

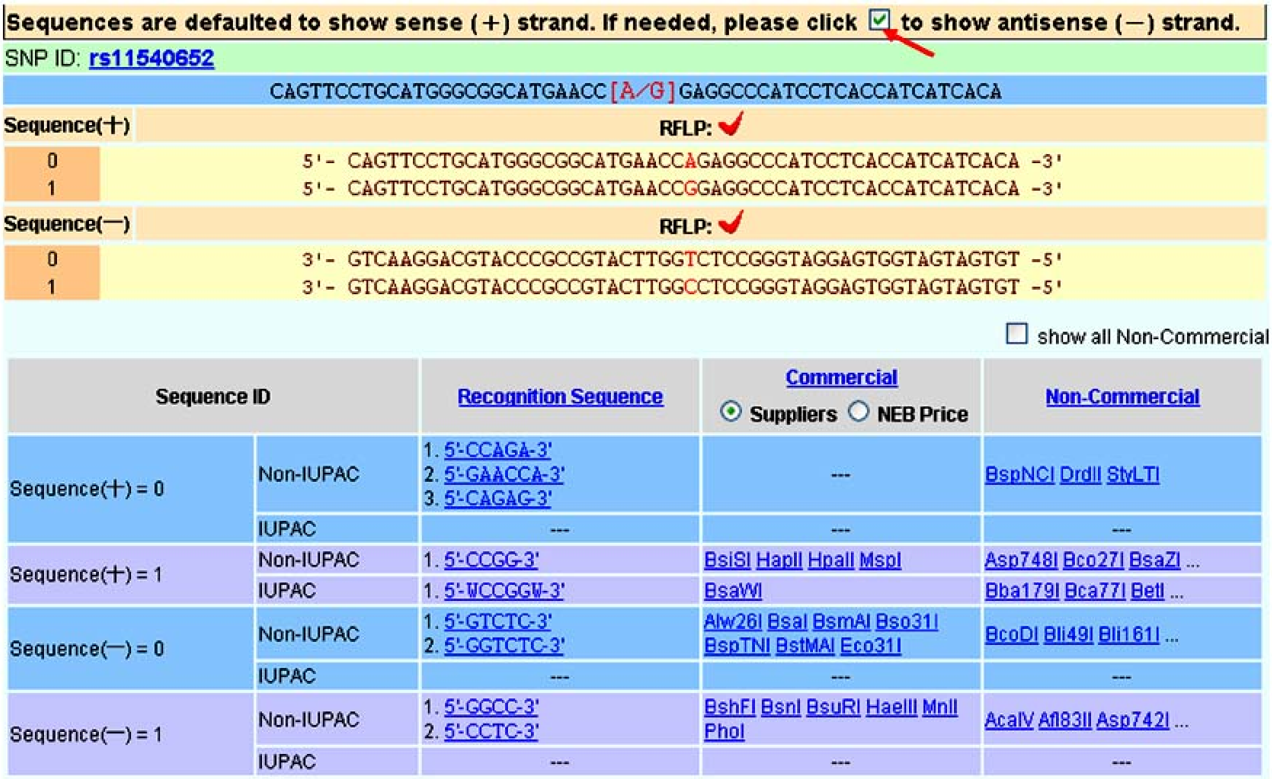

Fig. 2-18. The available sense and anti-sense strand restriction enzyme information for rs11540652 when the checkbox for anti-sense strand (indicated by a red arrow line) is checked. + is the sense strand and – is the antisense strand. 0 and 1 indicate the alternative nucleotide for the same SNP.

2.2.6 Example (6): Further restriction enzyme information description

The enzymes information contains: (1) “Sequence ID” for sense and anti-sense strands, (2) “Recognition Sequence”, and (3) “Commercial” and “Non-Commercial” enzymes.

For “Recognition Sequence”, this system provides the hyperlink for the same recognition sequence (Fig. 2-19). When the hyperlink for “Enzyme Name” is clicked, the search results for this enzyme name in NEW ENGLANE BioLabs (NEB) will be output (Fig. 2-20).

For “Commercial” enzymes, two options are available: one is ‘Suppliers’ and the other is ‘NEB Price’. When users select the ‘Suppliers’, the following enzyme hyperlinks will link to REBASE Suppliers (Fig. 2-21). When users select the ‘NEB Price’, the following enzymes hyperlink will link to price manager for Restriction Endonucleases which is our locally built-in database, all enzyme prices come from the price for NEB (Fig. 2-22 and Fig. 2-23). For “Non-Commercial” enzymes, enzymes hyperlink will be link to NCBI PubMed to search related documents (Fig. 2-24).

Prototype: Alw26I

Sequence ID	Recognition Enzyme In Sequence
0	5'- CAGTTCCTGCATGGGCGGCATGAACCAGAGGCCCATCCTCACCATCATCACA -3'
1	5'- CAGTTCCTGCATGGGCGGCATGAACCGGAGGCCCATCCTCACCATCATCACA -3'

ID	Enzyme Name	Microorganism	Source	Recognition Sequence	Methylation Site	Commercial Availability
1	Alw26I	Acinetobacter lwoffi RFL26	A. Janulaitis	GTCTC(1/5)	3(5),-4(6)	Fermentas International Inc.
2	BcoDI	Bacteroides coprocola DSM 17136	DSM 17136	GTCTC(1/5)		
3	BscQII	Bacillus species 4304	D. Clark	GTCTC		
4	BsmAI	Bacillus stearothermophilus A664	Z. Chen	GTCTC(1/5)	?(5)	New England Biolabs
5	BsoMAI	Bacillus stearothermophilus MA	S.K. Degtyarev	GTCTC(1/5)		
6	BstMAI	Bacillus stearothermophilus MA	S.K. Degtyarev	GTCTC(1/5)		

Fig. 2-19. The different enzyme names with the same recognition sequence ‘GTCTC’.

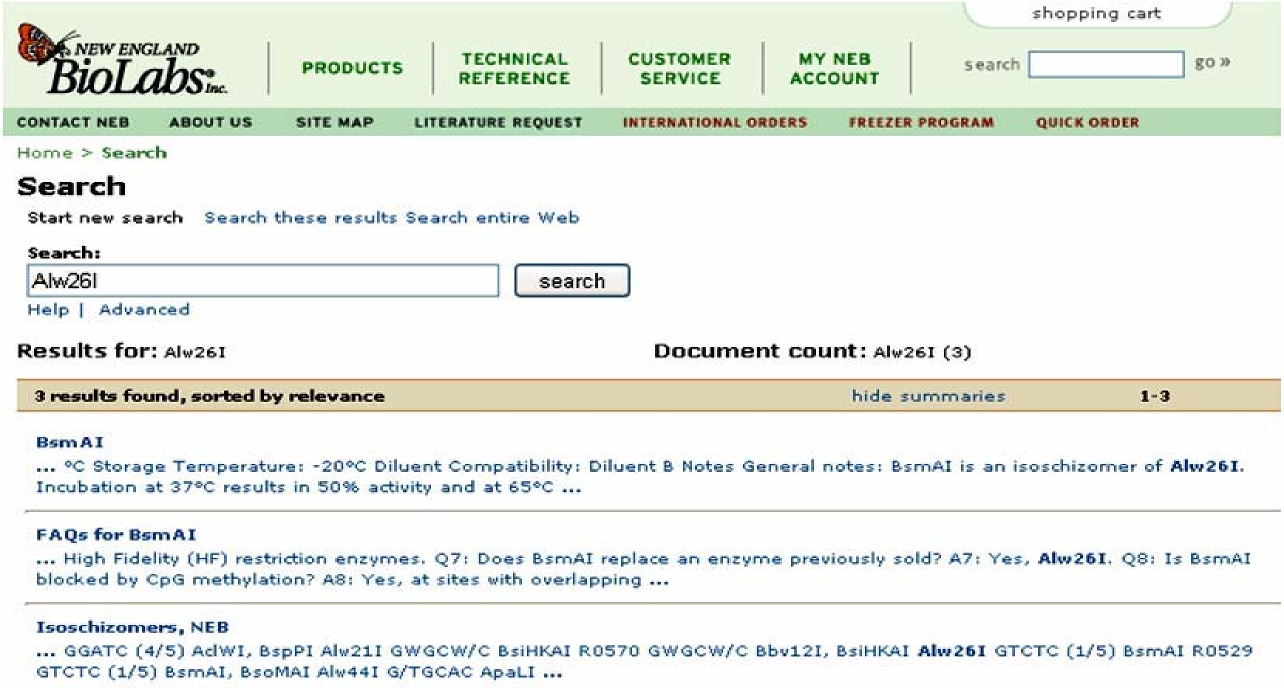

Fig. 2-20. The search results for Alw26I in NEW ENGLANE BioLabs.

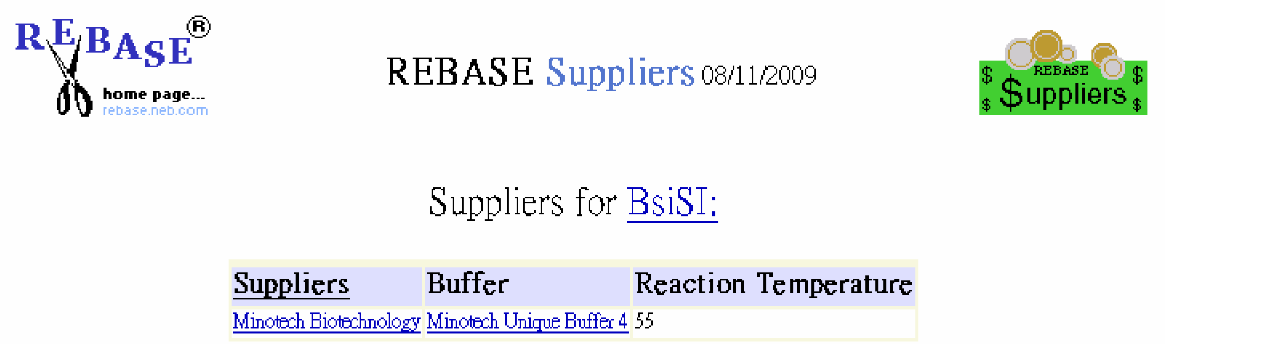

Fig. 2-21. “Commercial” enzymes option is ‘Suppliers’. Clicking the BsiSI enzyme will link to REBASE Suppliers and show the suppliers for BsiSI.

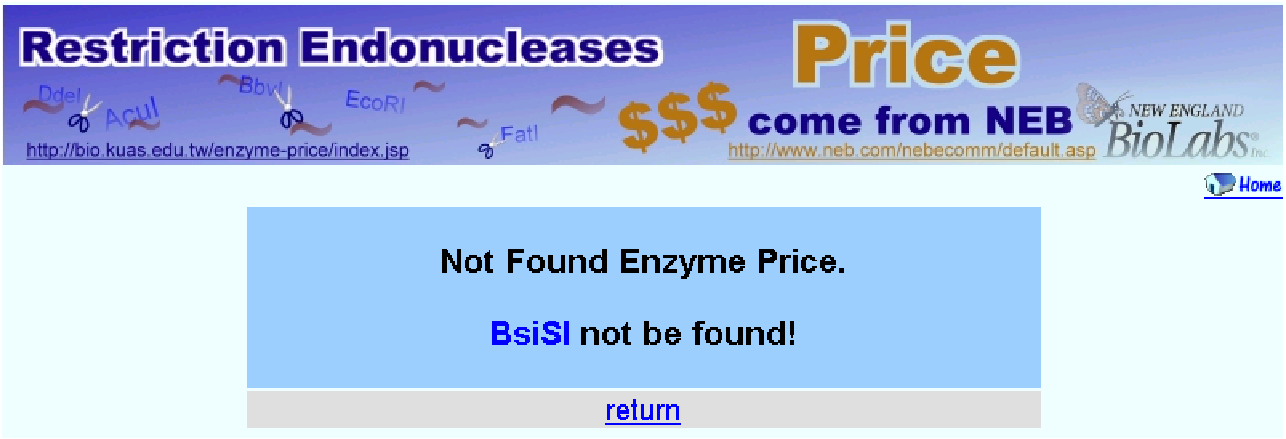

Fig. 2-22. For enzyme BsiSI, no any price is provided.

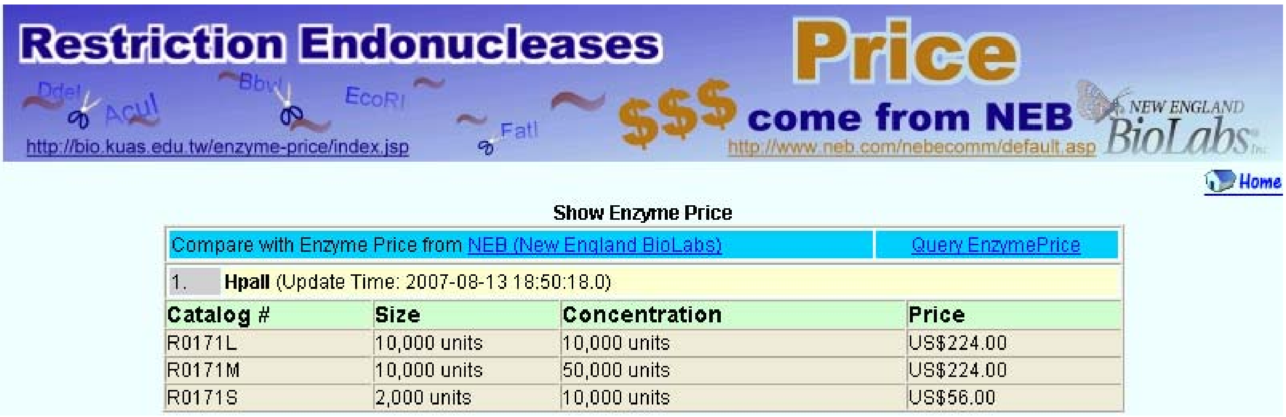

Catalog #	Size	Concentration	Price
R0171L	10,000 units	10,000 units	US$224.00
R0171M	10,000 units	50,000 units	US$224.00
R0171S	2,000 units	10,000 units	US$56.00

Fig. 2-23. For enzyme HpaII, the price is provided from NEB.

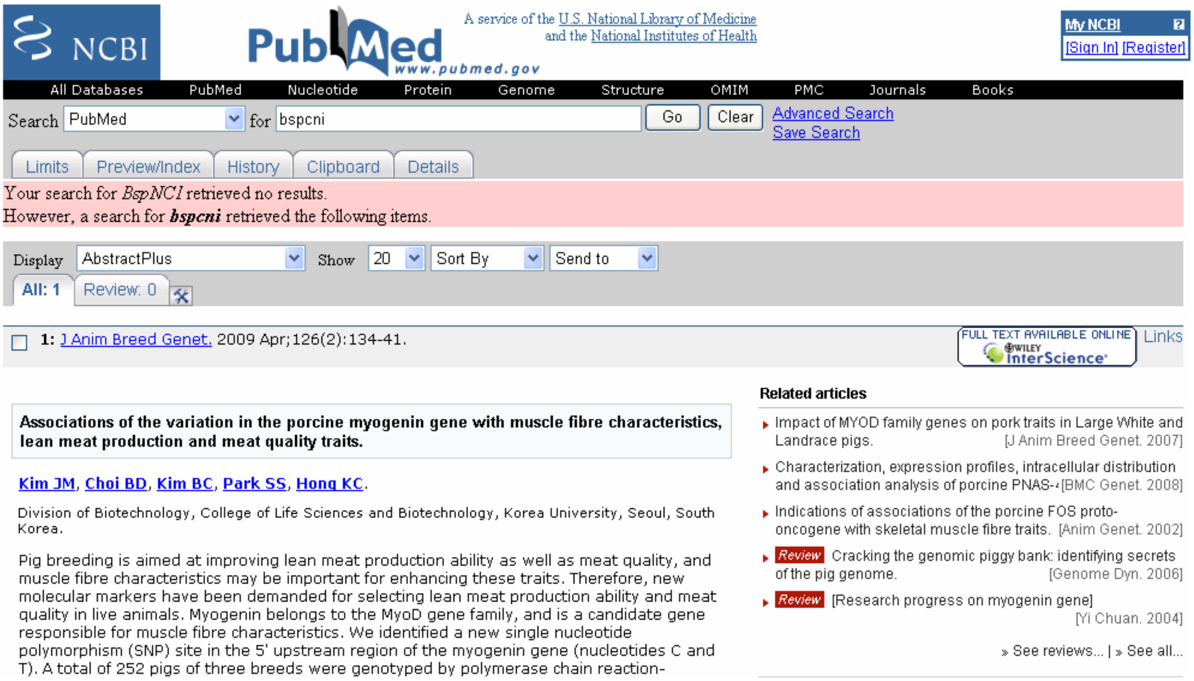

Fig. 2-24. Clicking the non-Commercial enzymes will be sent to NCBI PubMed to search related documents.

2.2.7 Example (7): Mutagenic primer information acquiring

Some SNPs are not recognized by any restriction enzymes. Alternatively, the mutagenic primer may be designed by the SNP-RFLPing 2 system. When a SNP does not have any restriction enzymes, the icon of "Mutagenic" will appear after SNP-RFLP analysis (Fig. 2-25). Clicking the icon of "Mutagenic", the mutagenic primer set information is appearing (Fig. 2-26). The mutagenic primer set information contains SNP ID, strand orientation, recognized allele, restriction enzymes, mutagenic primer pair information, and mutagenic primer visualization.

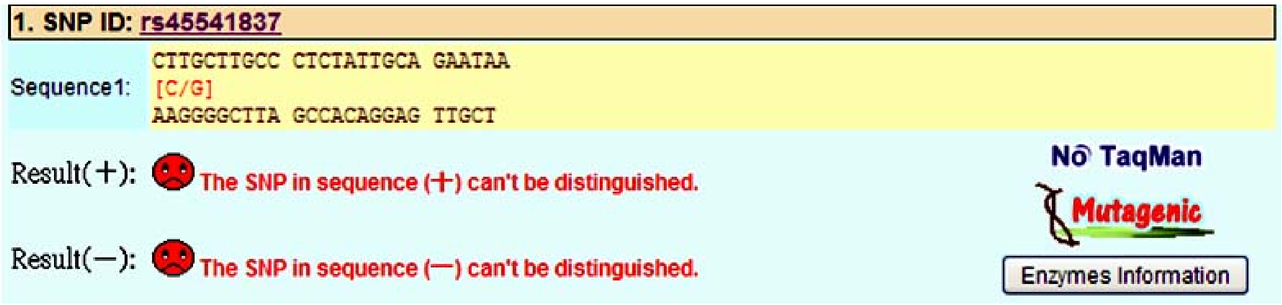

Fig. 2-25. No any available restriction enzymes for SNP rs45541837, the icon of "Mutagenic" appears in the result of SNP-RFLP analysis.

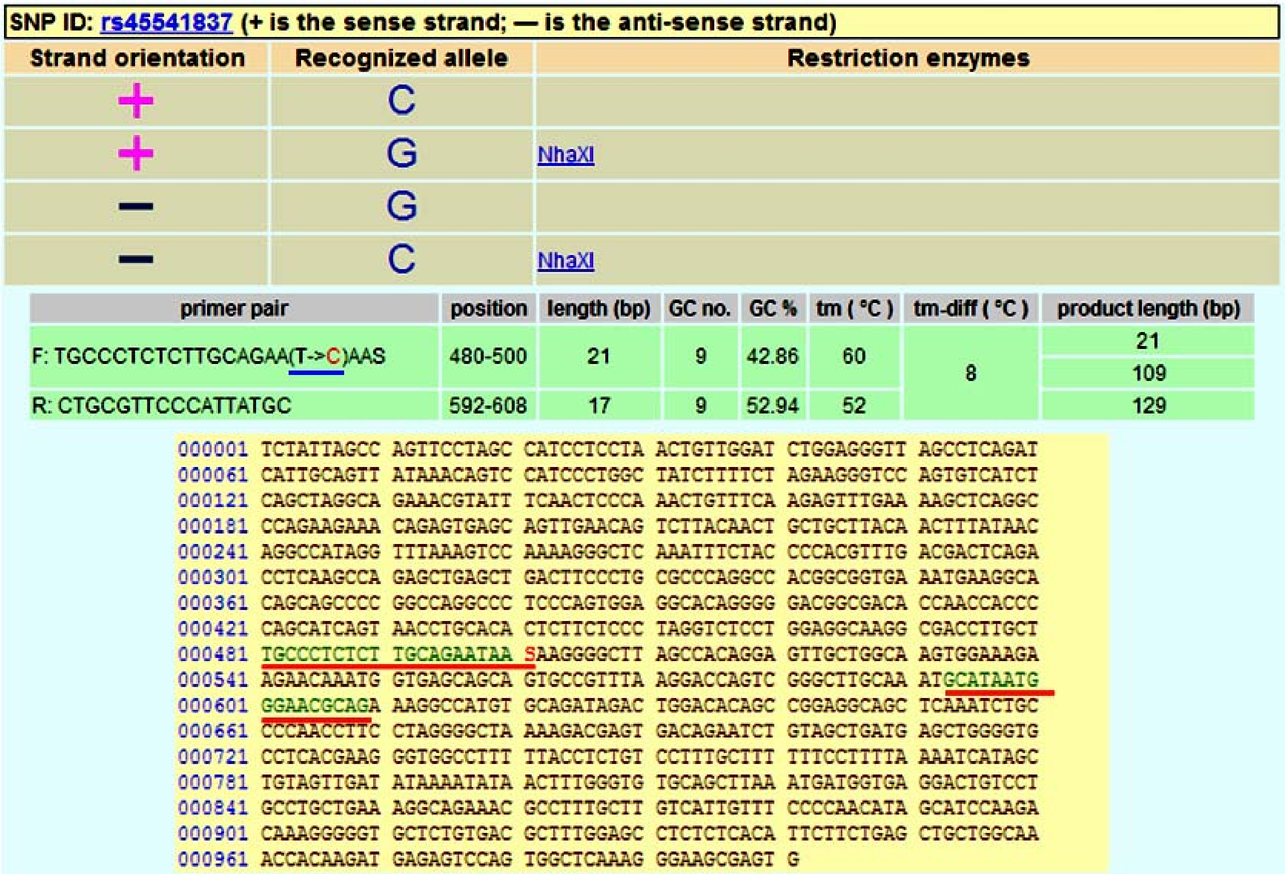

SNP ID: rs45541837 (+ is the sense strand; — is the anti-sense strand)

Strand orientation	Recognized allele	Restriction enzymes
+	C	
+	G	NhaXI
−	G	
−	C	NhaXI

primer pair	position	length (bp)	GC no.	GC %	tm (°C)	tm-diff (°C)	product length (bp)
F: TGCCCTCTCTTGCAGAA(T->C)AAS	480-500	21	9	42.86	60	8	21
							109
R: CTGCGTTCCCATTATGC	592-608	17	9	52.94	52		129

```
000001 TCTATTAGCC AGTTCCTAGC CATCCTCCTA ACTGTTGGAT CTGGAGGGTT AGCCTCAGAT
000061 CATTGCAGTT ATAAACAGTC CATCCCTGGC TATCTTTTCT AGAAGGGTCC AGTGTCATCT
000121 CAGCTAGGCA GAAACGTATT TCAACTCCCA AACTGTTTCA AGAGTTTGAA AAGCTCAGGC
000181 CCAGAAGAAA CAGAGTGAGC AGTTGAACAG TCTTACAACT GCTGCTTACA ACTTTATAAC
000241 AGGCCATAGG TTTAAAGTCC AAAAGGGCTC AAATTTCTAC CCCACGTTTG ACGACTCAGA
000301 CCTCAAGCCA GAGCTGAGCT GACTTCCCTG CGCCCAGGCC ACGGCGGTGA AATGAAGGCA
000361 CAGCAGCCCC GGCCAGGCCC TCCCAGTGGA GGCACAGGGG GACGGCGACA CCAACCACCC
000421 CAGCATCAGT AACCTGCACA CTCTTCTCCC TAGGTCTCCT GGAGGCAAGG CGACCTTGCT
000481 TGCCCTCTCT TGCAGAATAA SAAGGGGCTT AGCCACAGGA GTTGCTGGCA AGTGGAAAGA
000541 AGAACAAATG GTGAGCAGCA GTGCCGTTTA AGGACCAGTC GGGCTTGCAA ATGCATAATG
000601 GGAACGCAGA AAGGCCATGT GCAGATAGAC TGGACACAGC CGGAGGCAGC TCAAATCTGC
000661 CCCAACCTTC CTAGGGGCTA AAAGACGAGT GACAGAATCT GTAGCTGATG AGCTGGGGTG
000721 CCTCACGAAG GGTGGCCTTT TTACCTCTGT CCTTTGCTTT TTTCCTTTTA AAATCATAGC
000781 TGTAGTTGAT ATAAAATATA ACTTTGGGTG TGCAGCTTAA ATGATGGTGA GGACTGTCCT
000841 GCCTGCTGAA AGGCAGAAAC GCCTTTGCTT GTCATTGTTT CCCCAACATA GCATCCAAGA
000901 CAAAGGGGGT GCTCTGTGAC GCTTTGGAGC CTCTCTCACA TTCTTCTGAG CTGCTGGCAA
000961 ACCACAAGAT GAGAGTCCAG TGGCTCAAAG GGAAGCGAGT G
```

Fig. 2-26. The restriction enzymes and natural primer set information for rs45541837. The mutagenic position is underlined with green color in the F of primer pair field. Both forward and reverse primers are underlined with red color and the SNP is indicated by an arrow line. + is the sense strand and – is the antisense strand.

2.2.8 Example (8): TaqMan information providing

In above SNP (rs11540652), its TaqMan probe is not provided in both NCBI and SNP500Cancer (Fig. 2-15). However, some SNPs, such as rs1318703 which is shown on SNP ID example (2) (Fig. 2-27) and rs1202183 which is shown on SNP ID example (4) - ss1717832 (Fig. 2-28) provide TaqMan probe. When a SNP provide TaqMan probe, the icon of “TaqMan” will appear in the result of SNP-RFLP analysis.

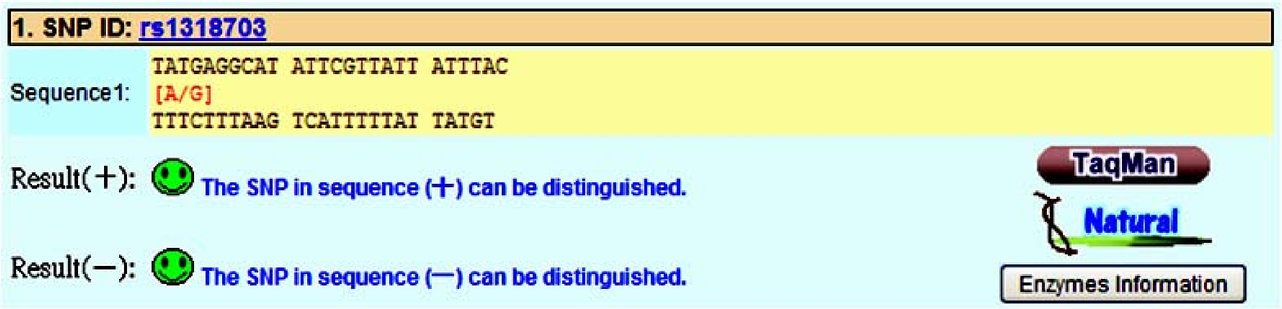

Fig. 2-27. The icon of TaqMan appears in rs1318703 which is in SNP ID example (2).

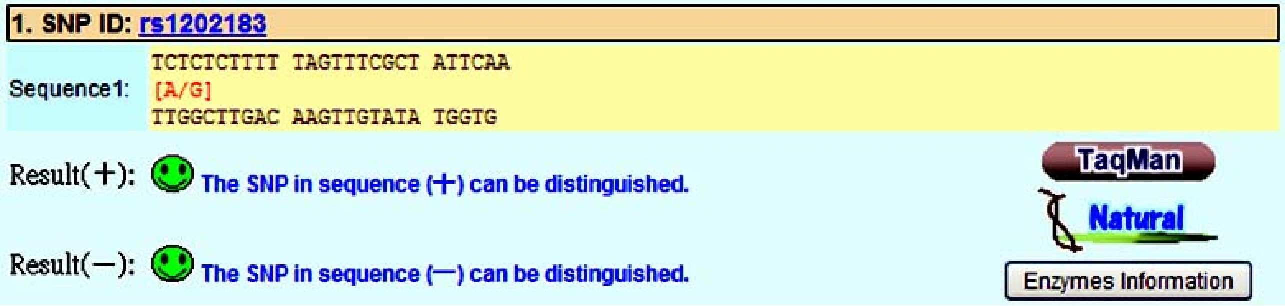

Fig. 2-28. The icon of TaqMan appears in rs1202183 which is in SNP ID example (4) - ss1717832.

User can click the icon of “TaqMan”, and then TaqMan information for this SNP will be represented. Fig. 2-29 and Fig. 2-30 show the available TaqMan information for rs1318703 and rs1202183, respectively.

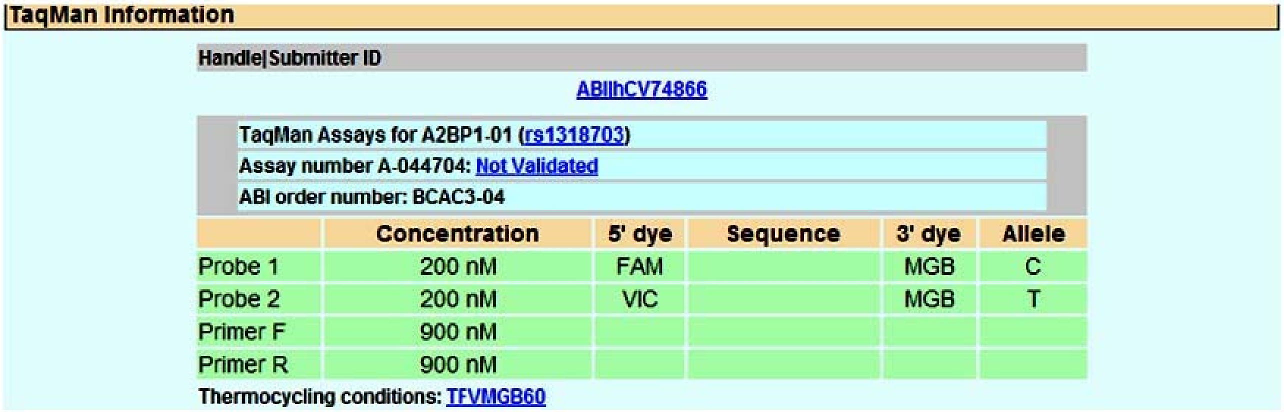
TaqMan Information

Handle|Submitter ID

ABI|hCV74866

TaqMan Assays for A2BP1-01 (rs1318703)
Assay number A-044704: Not Validated
ABI order number: BCAC3-04

	Concentration	5' dye	Sequence	3' dye	Allele
Probe 1	200 nM	FAM		MGB	C
Probe 2	200 nM	VIC		MGB	T
Primer F	900 nM				
Primer R	900 nM				

Thermocycling conditions: TFVMGB60

Fig. 2-29. The TaqMan information for rs1318703 is shown after clicking the icon of “TaqMan” in Fig. 2-27.

TaqMan Information

Handle|Submitter ID

SNP500CANCER|ABCB1-04

TaqMan Assays for ABCB1-04 (rs1202183)
Assay number C___7586795_20: Validated
ABI order number: 1

	Concentration	5' dye	Sequence	3' dye	Allele
Probe 1	200 nM	FAM		MGB	A
Probe 2	200 nM	VIC		MGB	G
Primer F	900 nM				
Primer R	900 nM				

Thermocycling conditions: TFVMGB60

Fig. 2-30. The TaqMan information for rs1202183 is shown after clicking the icon of “TaqMan” in Fig. 2-28.

3. Function: SNP in fasta sequence input

3.1 Overview

SNP in fasta sequence format is acceptable to query the SNP-RFLP information in SNP-RFLPing 2. Single fasta sequence, multiple fasta sequences with SNPs in [dNTP1/dNTP2] or IUPAC formats, and special sequences with SNP in the indel (insertion and deletion), tri-allelic and tetra-allelic formats are all acceptable.

Fig. 3-1 shows a regular single fasta sequence.

Fig. 3-2 shows a single fasta sequence with line (press the enter key of keyboard) which is ignored in the system.

Fig. 3-3 and Fig. 3-5 show regular multiple fasta sequences with SNPs in [dNTP1/dNTP2] and IUPAC format, respectively.

Fig. 3-4 shows multiple fasta sequences with SNPs in [dNTP1/dNTP2].

Fig. 3-6 shows the IUPAC format with lines (press the enter key of keyboard) which are ignored.

Fig. 3-7 to Fig. 3-9 are special sequence with SNP in the in-del, tri-allelic and tetra-allelic formats, respectively. In the SNP fasta sequence input, the uppercase or lowercase letters are acceptable.

3.2 Example: Use SNP in fasta sequence format to analyze

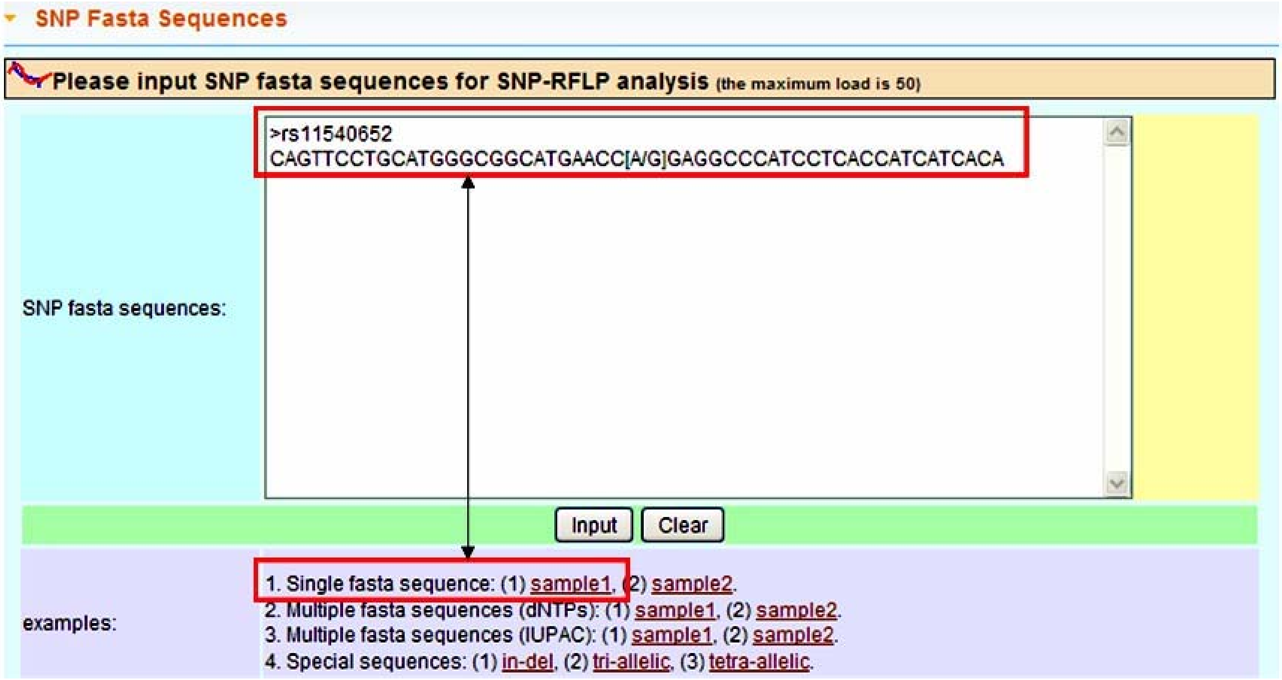

Fig. 3-1. SNP in a regular single fasta sequence.

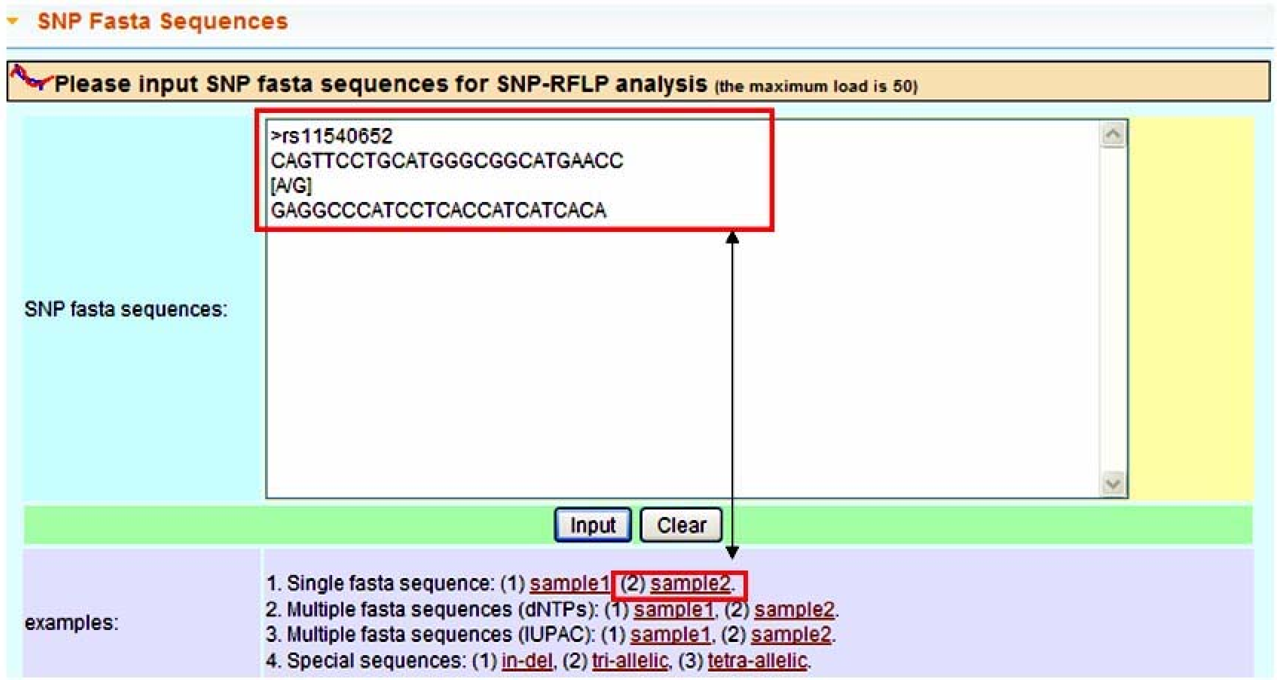

Fig. 3-2. SNP in a single fasta sequence with line (press the enter key of keyboard) which is ignored in the system. When the sequence is too long, the part of the sequence will list in the next line automatically. The system is able to regard them as the single sequence input.

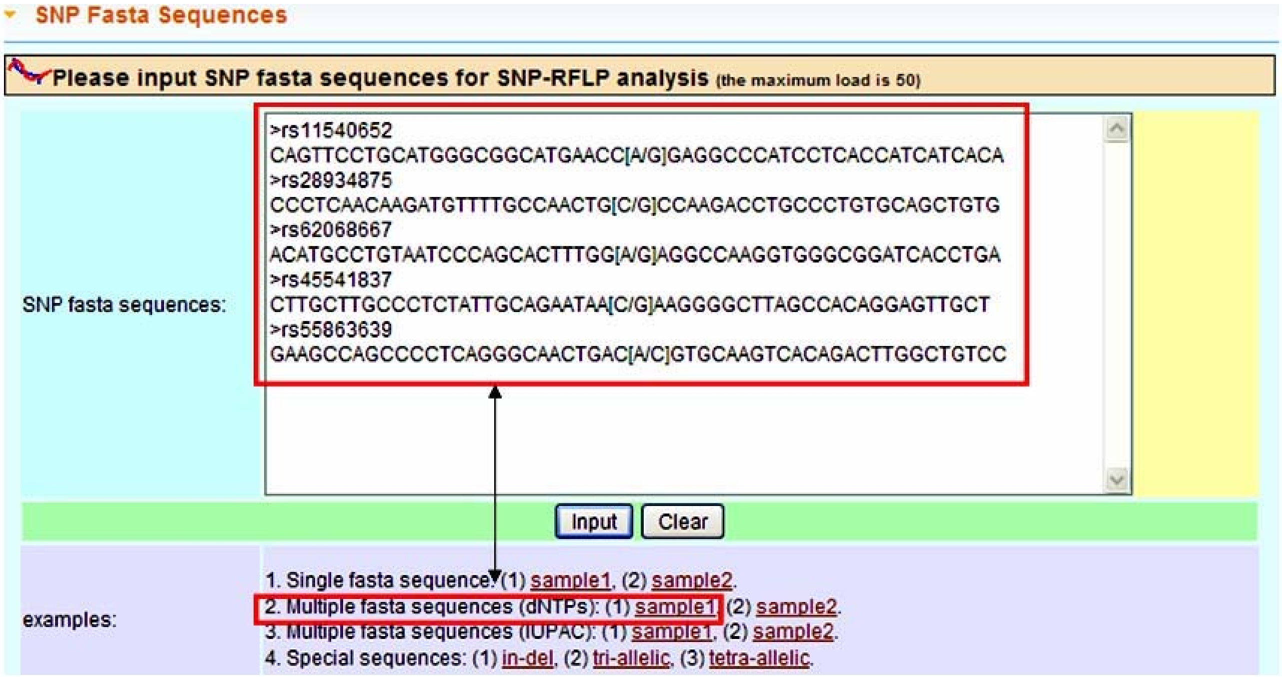

Fig. 3-3. Regular multiple fasta sequences with SNPs in [dNTP1/dNTP2] format.

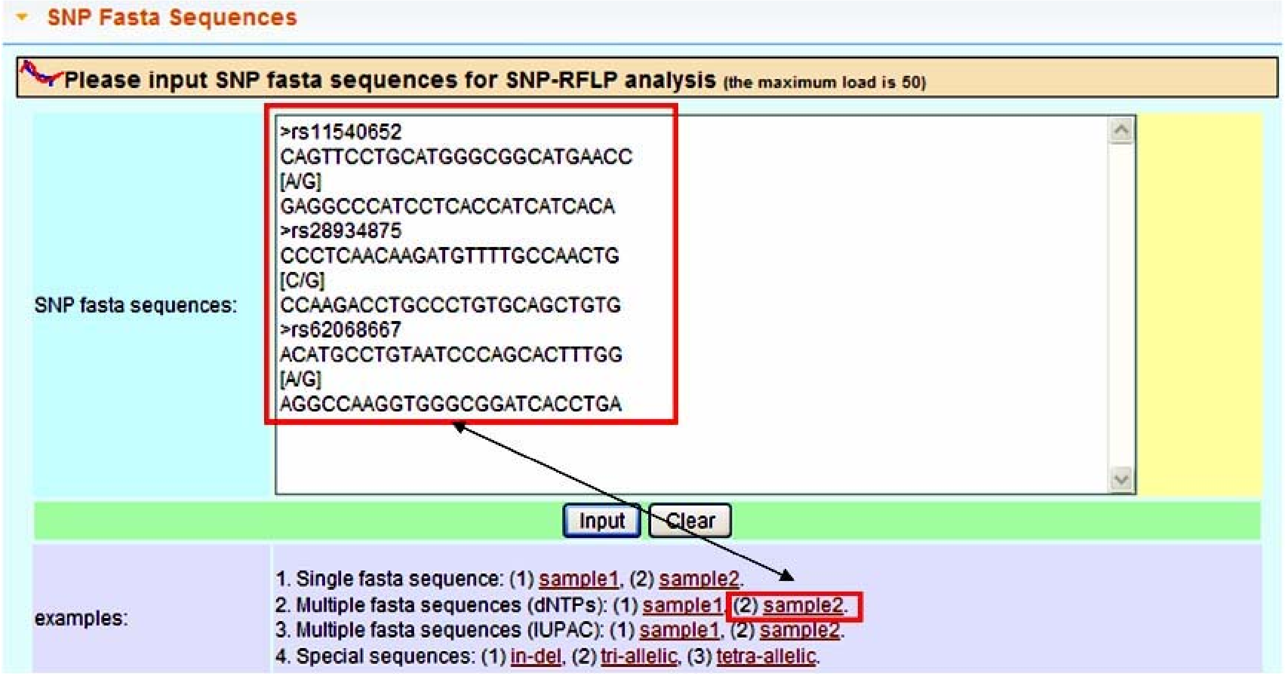

Fig. 3-4. Multiple fasta sequences with SNPs in [dNTP1/dNTP2] format with line (press the enter key of keyboard) which are ignored in the system. When the sequence is too long, the part of the sequence will list in the next line automatically. The system is able to regard them as the single sequence input.

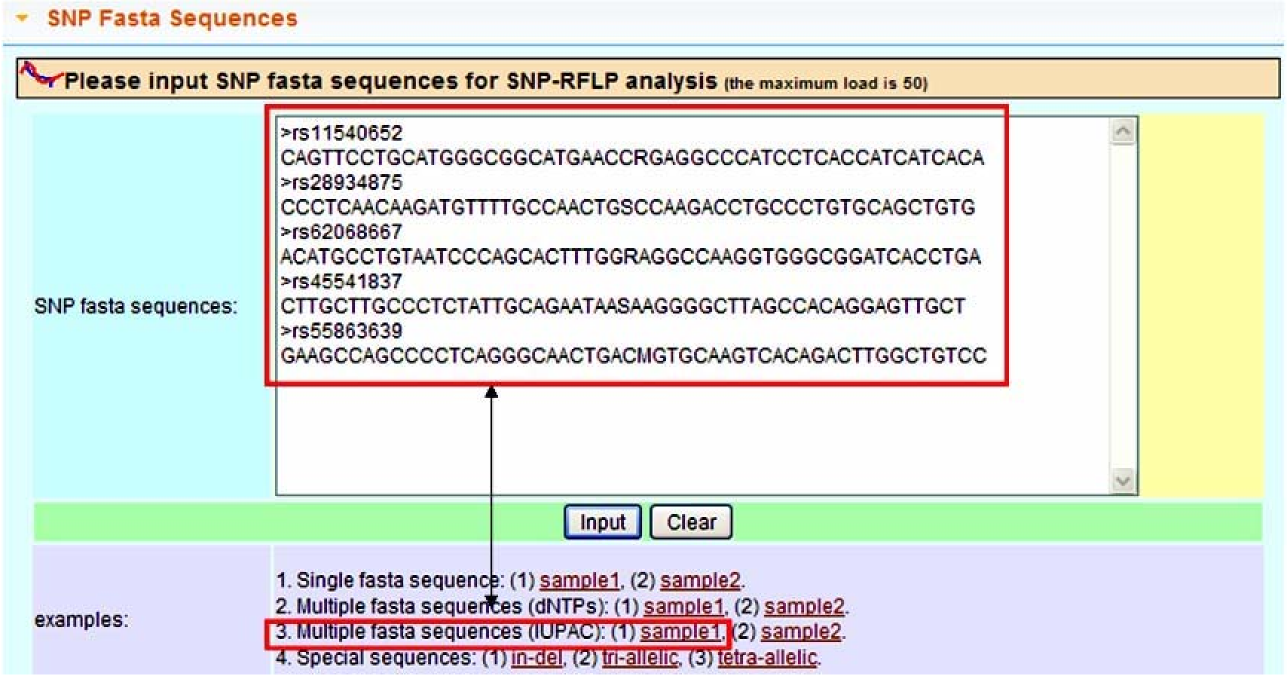

Fig. 3-5. Regular multiple fasta sequences with SNPs in IUPAC format.

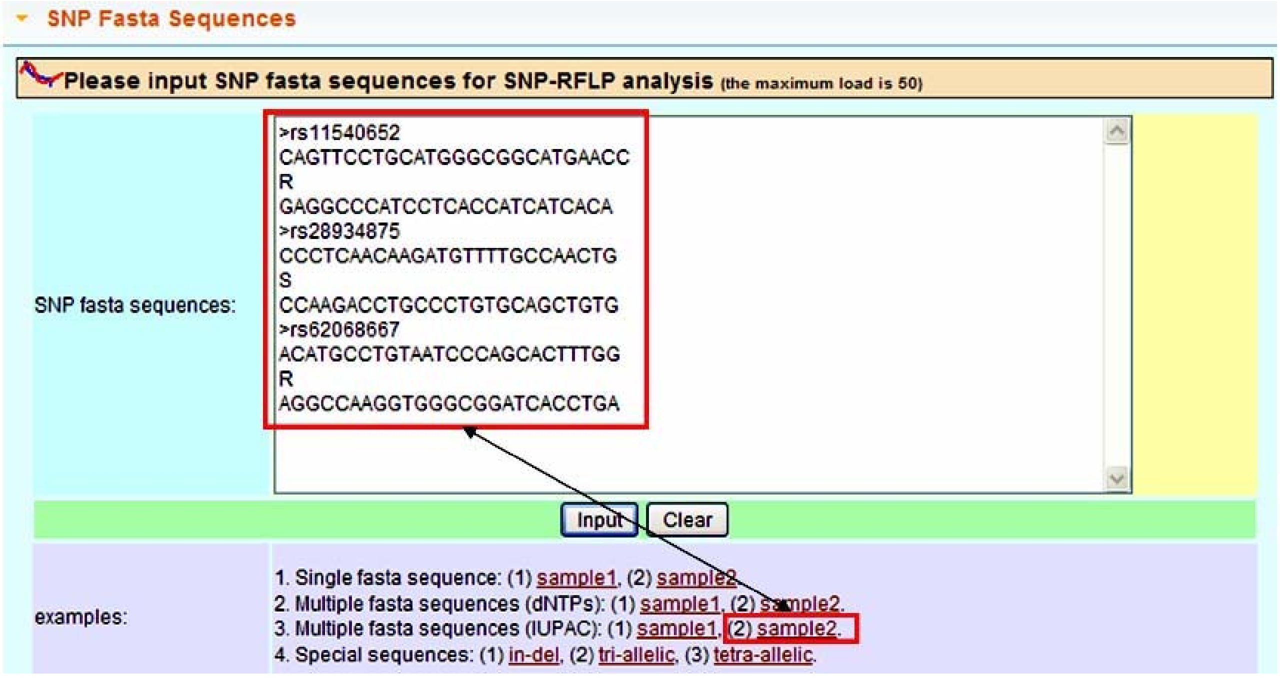

Fig. 3-6. Multiple fasta sequences with SNPs in IUPAC format with line (press the enter key of keyboard) which is ignored in the system. R = G or A; S = G or C. When the sequence is too long, the part of the sequence will list in the next line automatically. The system is able to regard them as the single sequence input.

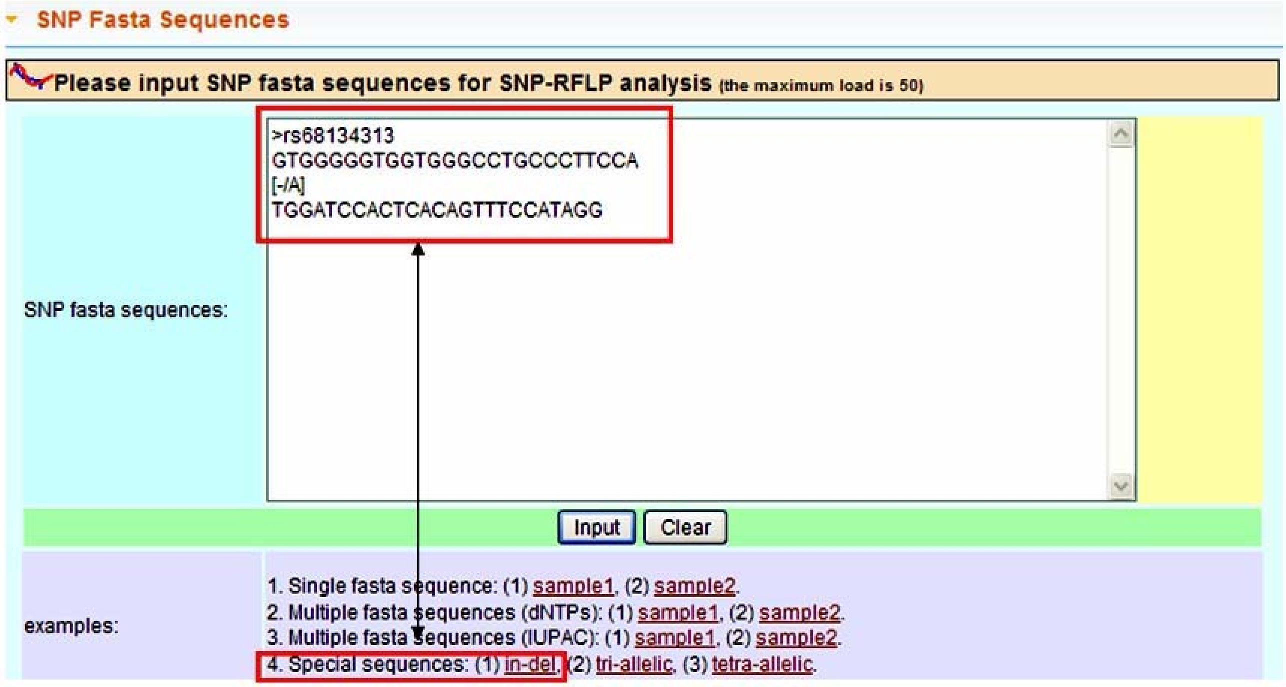

Fig. 3-7. Special sequence with SNP in the in-del format. When the sequence is too long, the part of the sequence will list in the next line automatically. The system is able to regard them as the single sequence input.

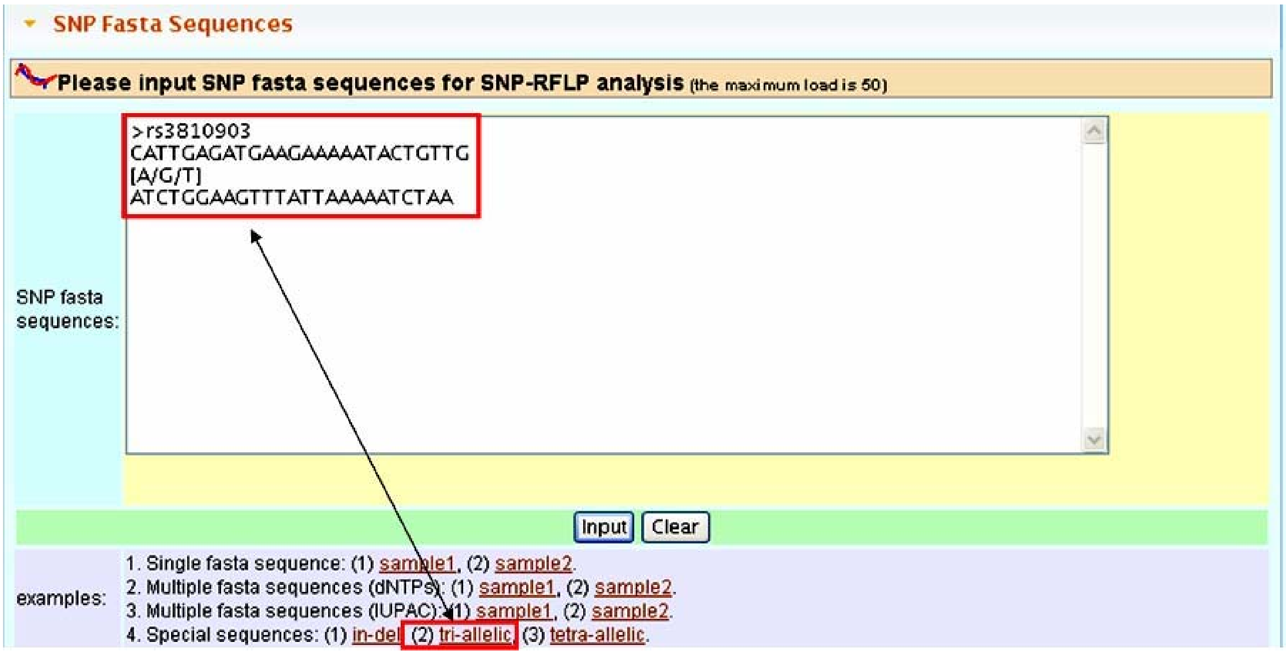

Fig. 3-8. Special sequence with SNP in the tri-allelic format. When the sequence is too long, the part of the sequence will list in the next line automatically. The system is able to regard them as the single sequence input.

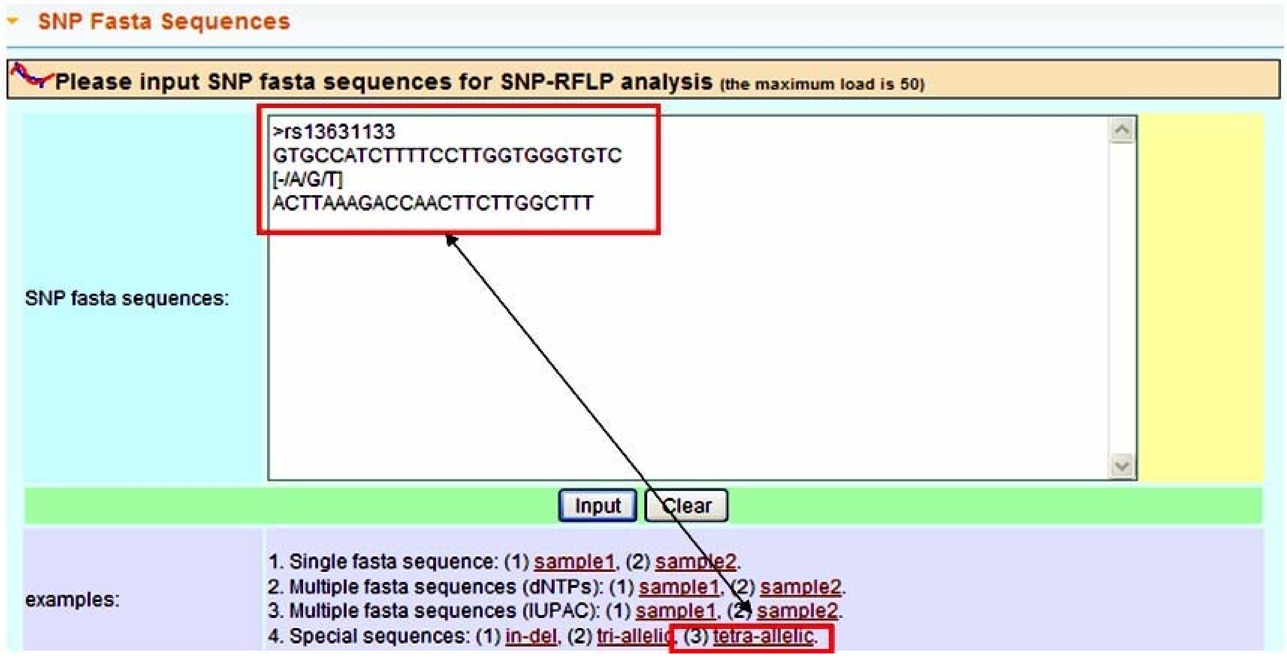

Fig. 3-9. Special sequence with SNP in the tetra-allelic format. When the sequence is too long, the part of the sequence will list in the next line automatically. The system is able to regard them as the single sequence input.

After inputting the SNP fasta sequence as mentioned above, users can click the “Input” button, and all information which is not belong to ‘A’, ‘T’, ‘C’, ‘T’, [dNTP1/dNTP2/…/ dNTPn], or IUPAC will be filtered out. The results for SNP fasta sequence input are shown as Fig. 3-10, including: (1) “All selected”, (2) “Clear”, (3) “Analyze sequence”, (4) the description for sequence, and (5) SNP sequence. Users can check the desired SNPs and click the “Analyze sequence” to next step. If the all SNPs need be analyzed, the “All selected” button provides convenience to check all SNPs. The “Clear” button provides cancel all SNP checked.

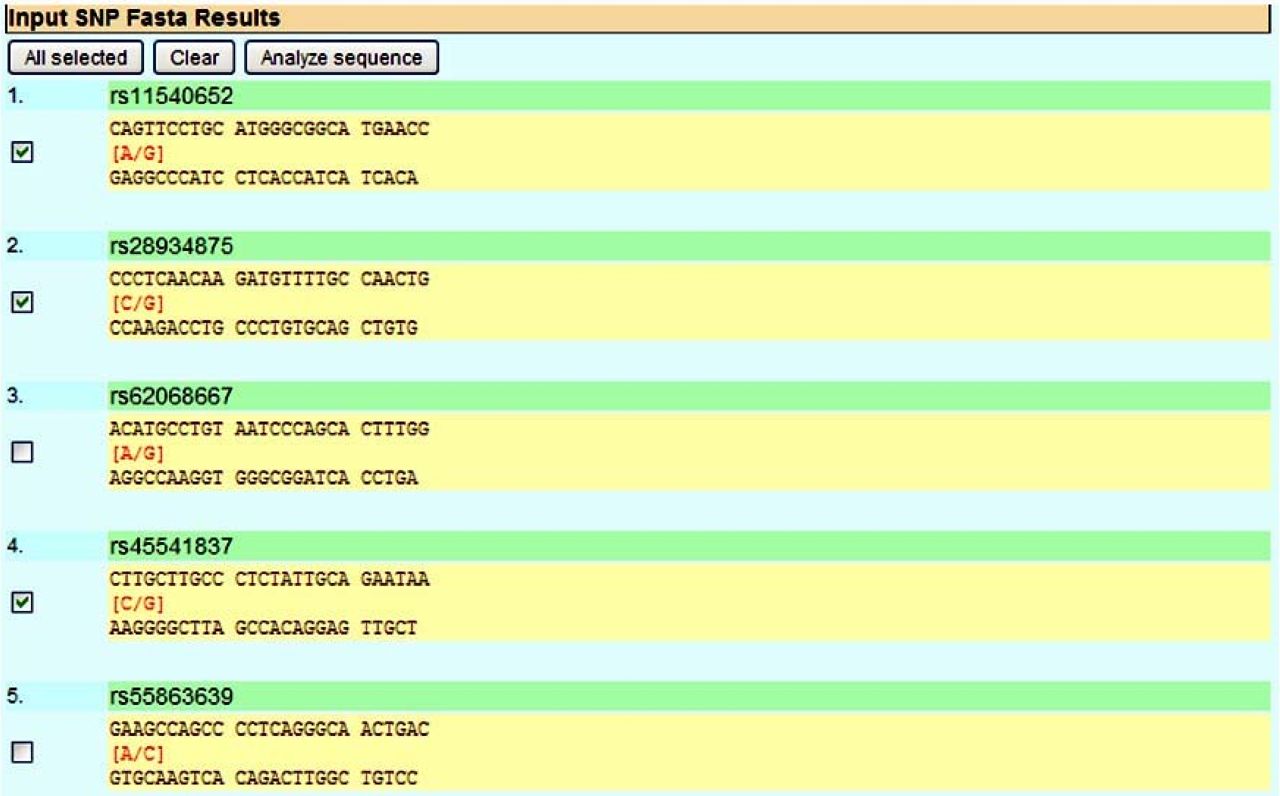

Fig. 3-10. The results for SNP fasta sequence input.

When clicking the “Analyze sequence”, users need to wait for the analysis of SNP-RFLP recognition (Fig. 3-11). Then, the results for SNP-RFLP recognition will be presented (Fig. 3-12). Finally, users can select the interested SNPs as described above.

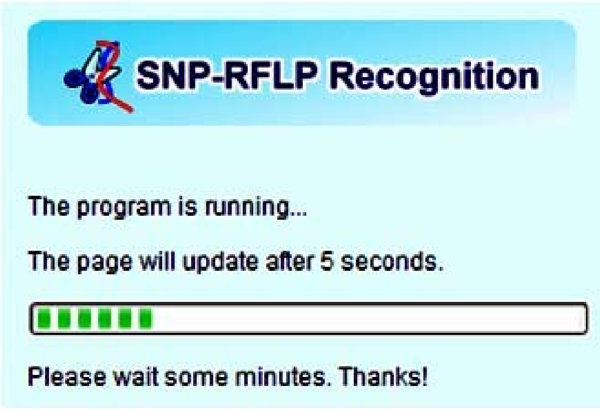

Fig. 3-11. Wait for SNP-RFLP recognition.

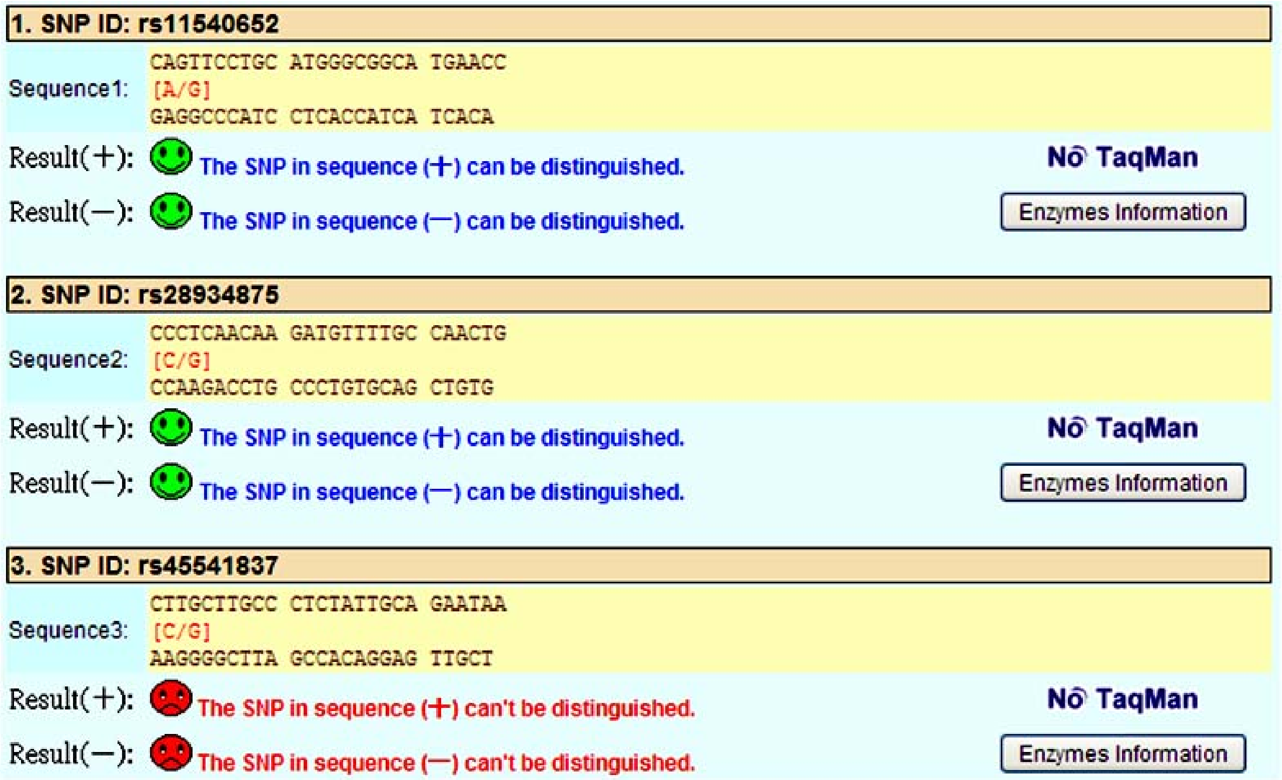

Fig. 3-12. The results for SNP-RFLP recognition.

When uses input a fasta sequence with SNP in the in-del (insertion and deletion), tri-allelic, and tetra-allelic formats, their results are described as follows.

For SNP in the in-del format, the results are similar to di-allelic SNP (Fig. 3-13 and Fig. 3-14).

For SNP in the tri-allelic format, the results for restriction enzymes information will present three sequence IDs with their corresponding available restriction enzymes for three different alleles (Fig. 3-15).

For SNP in the tetra-allelic format, the results for restriction enzymes information will present four sequence IDs with their corresponding available restriction enzymes for four different alleles (Fig. 3-16).

For input SNP in fasta sequence format, the results for SNP-RFLP recognition will show the natural or mutagenic design results, because the SNP flanking sequence is not provided by the user input sequence.

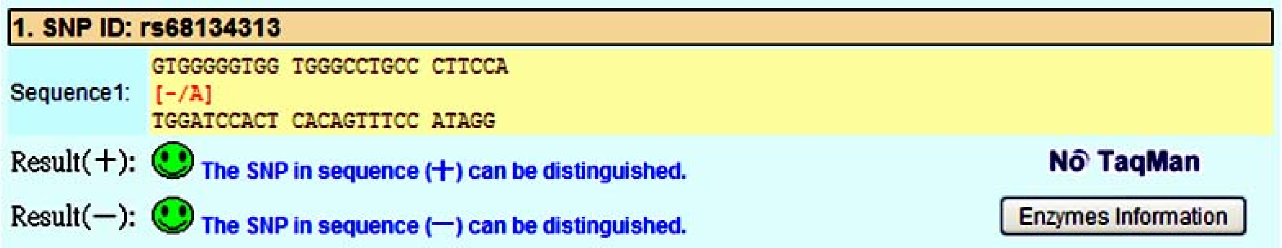

Fig. 3-13. The result for SNP-RFLP recognition with SNP in the in-del farmat.

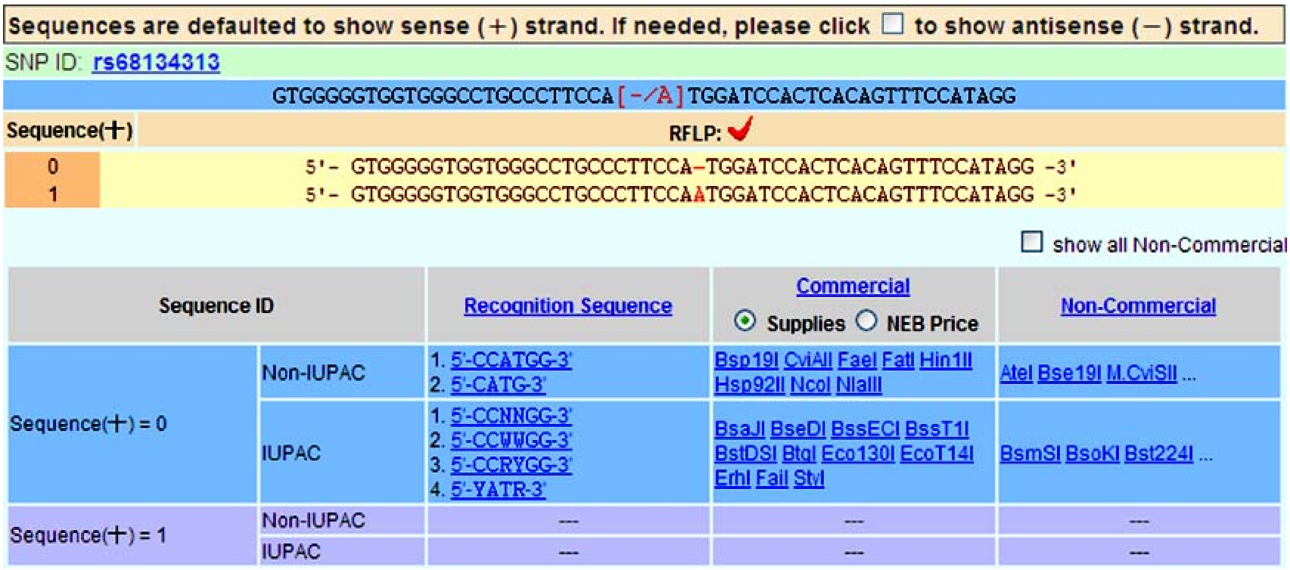

Sequences are defaulted to show sense (+) strand. If needed, please click ☐ to show antisense (−) strand.

SNP ID: rs68134313

GTGGGGGTGGTGGGCCTGCCCTTCCA[-/A]TGGATCCACTCACAGTTTCCATAGG

Sequence(+)	RFLP: ✔
0	5'- GTGGGGGTGGTGGGCCTGCCCTTCCA-TGGATCCACTCACAGTTTCCATAGG -3'
1	5'- GTGGGGGTGGTGGGCCTGCCCTTCCAATGGATCCACTCACAGTTTCCATAGG -3'

☐ show all Non-Commercial

Sequence ID		Recognition Sequence	Commercial (⦿ Supplies ○ NEB Price)	Non-Commercial
Sequence(+) = 0	Non-IUPAC	1. 5'-CCATGG-3'<br>2. 5'-CATG-3'	Bsp19I CviAII FaeI FatI Hin1II Hsp92II NcoI NlaIII	AteI Bse19I M.CviSII ...
	IUPAC	1. 5'-CCNNGG-3'<br>2. 5'-CCWWGG-3'<br>3. 5'-CCRYGG-3'<br>4. 5'-YATR-3'	BsaJI BseDI BssECI BssT1I BstDSI BtgI Eco130I EcoT14I ErhI FaiI StyI	BsmSI BsoKI Bst2241 ...
Sequence(+) = 1	Non-IUPAC	---	---	---
	IUPAC	---	---	---

Fig. 3-14. The available restriction enzyme information for the sense strand with SNP in the in-del format.

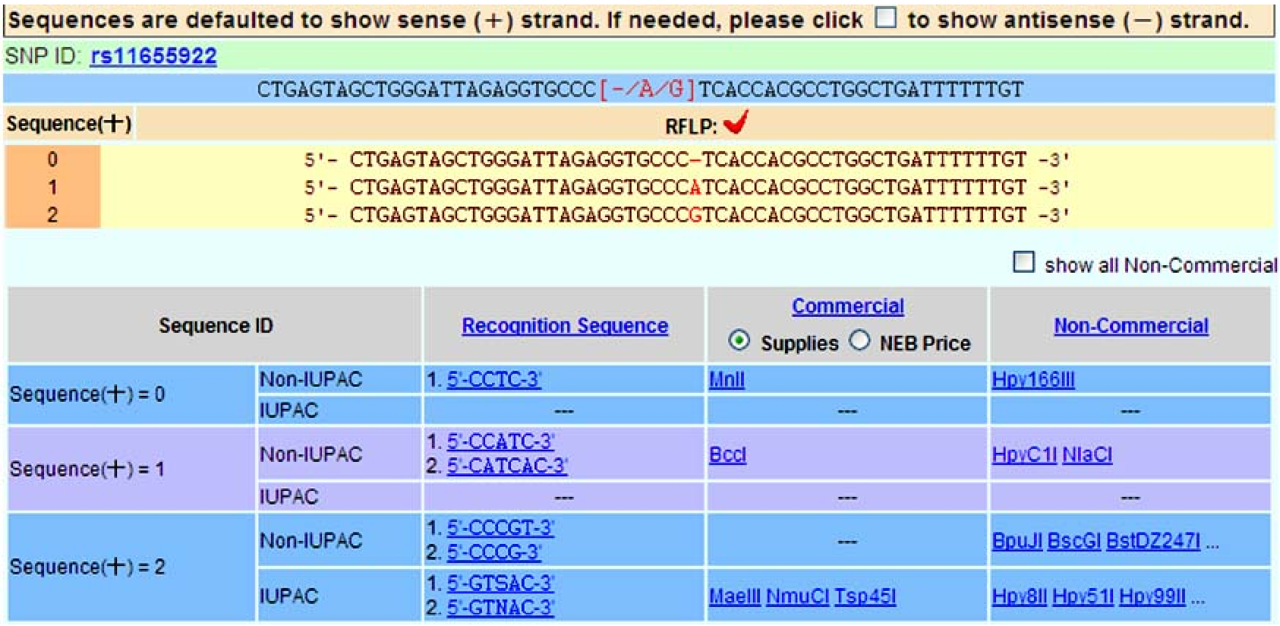

Sequences are defaulted to show sense (+) strand. If needed, please click ☐ to show antisense (−) strand.

SNP ID: rs11655922

CTGAGTAGCTGGGATTAGAGGTGCCC[-/A/G]TCACCACGCCTGGCTGATTTTTTGT

Sequence(+)	RFLP: ✔
0	5'- CTGAGTAGCTGGGATTAGAGGTGCCC-TCACCACGCCTGGCTGATTTTTTGT -3'
1	5'- CTGAGTAGCTGGGATTAGAGGTGCCCATCACCACGCCTGGCTGATTTTTTGT -3'
2	5'- CTGAGTAGCTGGGATTAGAGGTGCCCGTCACCACGCCTGGCTGATTTTTTGT -3'

☐ show all Non-Commercial

Sequence ID		Recognition Sequence	Commercial (⦿ Supplies ○ NEB Price)	Non-Commercial
Sequence(+) = 0	Non-IUPAC	1. 5'-CCTC-3'	MnlI	Hpy166III
	IUPAC	---	---	---
Sequence(+) = 1	Non-IUPAC	1. 5'-CCATC-3'<br>2. 5'-CATCAC-3'	BccI	HpyC1I NlaCI
	IUPAC	---	---	---
Sequence(+) = 2	Non-IUPAC	1. 5'-CCCGT-3'<br>2. 5'-CCCG-3'	---	BpuJI BscGI BstDZ247I ...
	IUPAC	1. 5'-GTSAC-3'<br>2. 5'-GTNAC-3'	MaeIII NmuCI Tsp45I	Hpy8II Hpy51I Hpy99II ...

Fig. 3-15. The available restriction enzyme information for the sense strand with SNP in the tri-allelic format.

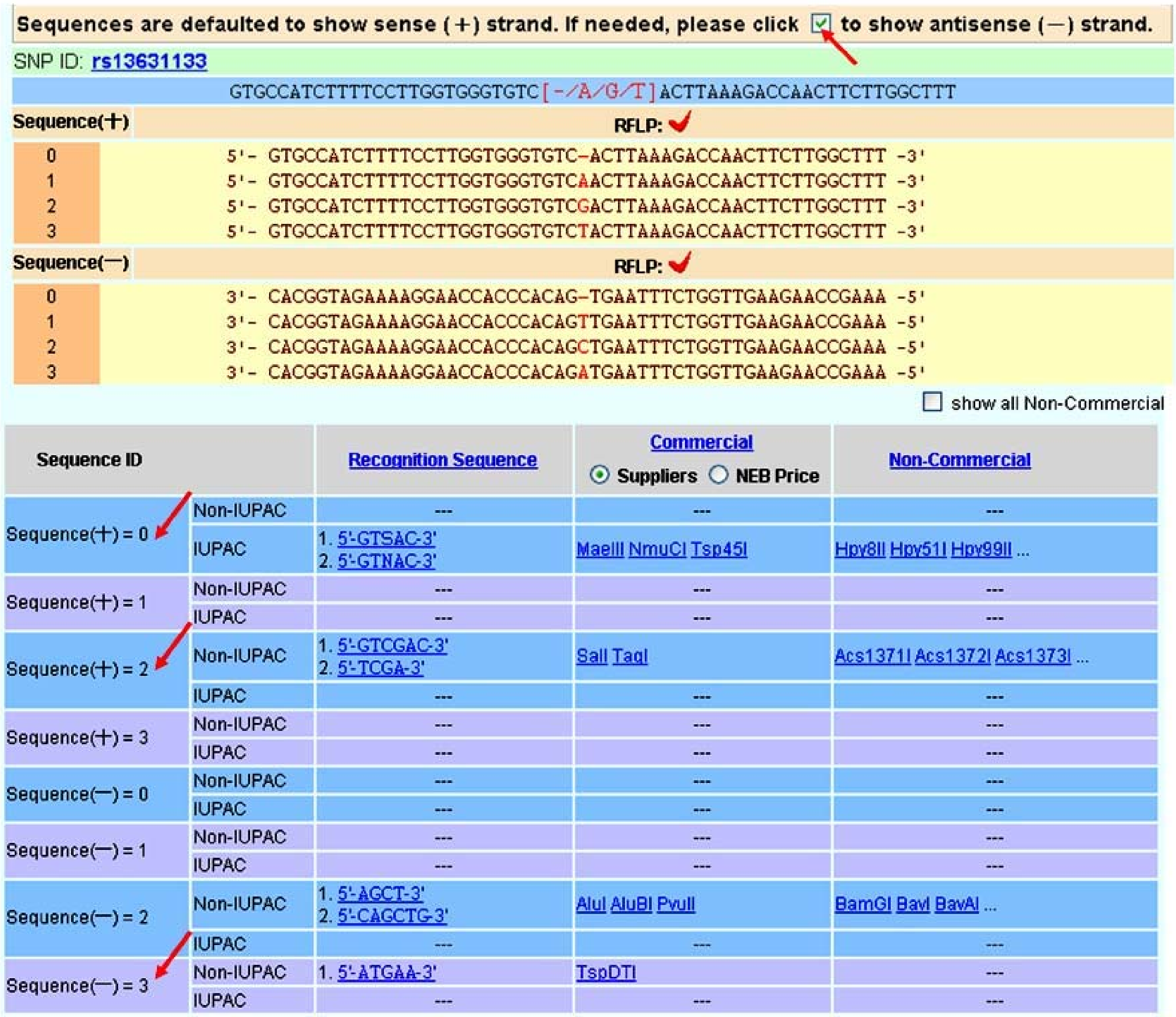

Fig. 3-16. The available restriction enzyme information for the sense strand with SNP in the tetra-allelic format. In this case, the enzymes for the antisense strand are chosen (*as indicated by red arrow in the top*). In the left side, three red arrow lines indicate that three nucleotides are distinguished between each other. The sequence 1 is unable to find the suitable enzyme but it is still distinct to others because it is unable to cut by the enzymes listed for sequences 0, 2, and 3.

4. Function: Multiple SNPs within one sequence

4.1 Overview

In the function for multiple SNPs within one sequence, up to 50 SNPs representing in the [dNTP1/dNTP2] or IUPAC formats within the input sequence are acceptable for analysis. The flanking sequences for two nearby SNPs should not be overlapped within 6 nucleotides. There are two input areas in the function: one is “Description” which is a title for describing input sequence and the other is “Sequence” which is input multiple SNPs sequence.

Fig. 4-1 and Fig. 4-2 are two SNPs within one sequence in the [dNTP1/dNTP2] and IUPAC formats, respectively.

Fig. 4-3 and Fig. 4-4 are three SNPs within one sequence in the [dNTP1/dNTP2] and IUPAC formats, respectively.

Fig. 4-5 and Fig. 4-6 are four SNPs within one sequence in the [dNTP1/dNTP2] and IUPAC formats, respectively.

4.2 Example: Multiple SNPs within one sequence input

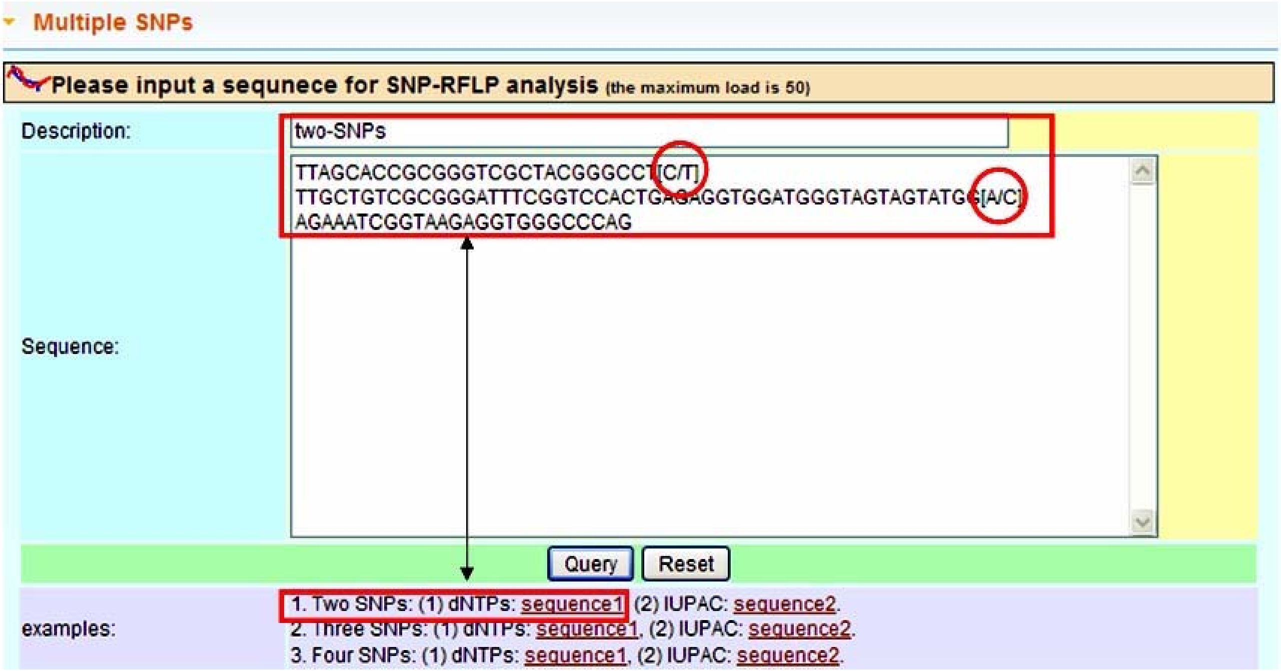

Fig. 4-1. Two SNPs within one sequence in the [dNTP1/dNTP2] format. When the sequence is too long, the part of the sequence will list in the next line automatically. The system is able to regard them as the single sequence input. If the SNP is marked in IUPAC code, the sequence is continued as shown in Fig. 4-2.

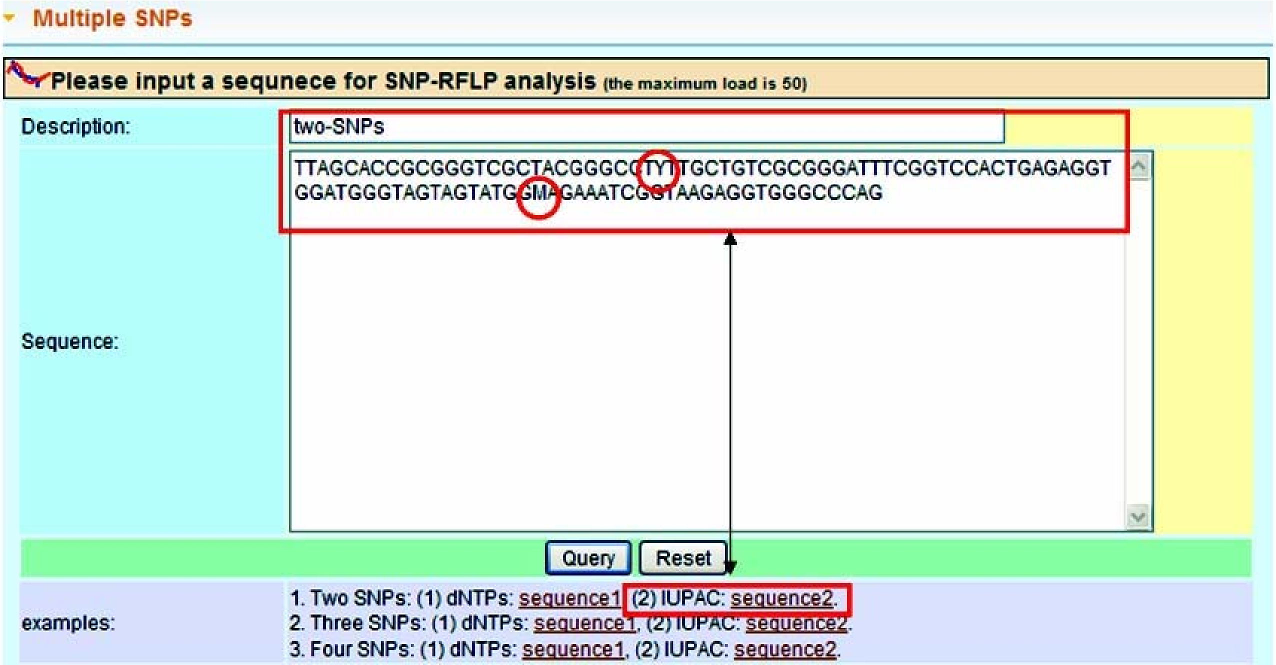

Fig. 4-2. Two SNPs within one sequence in the IUPAC format.

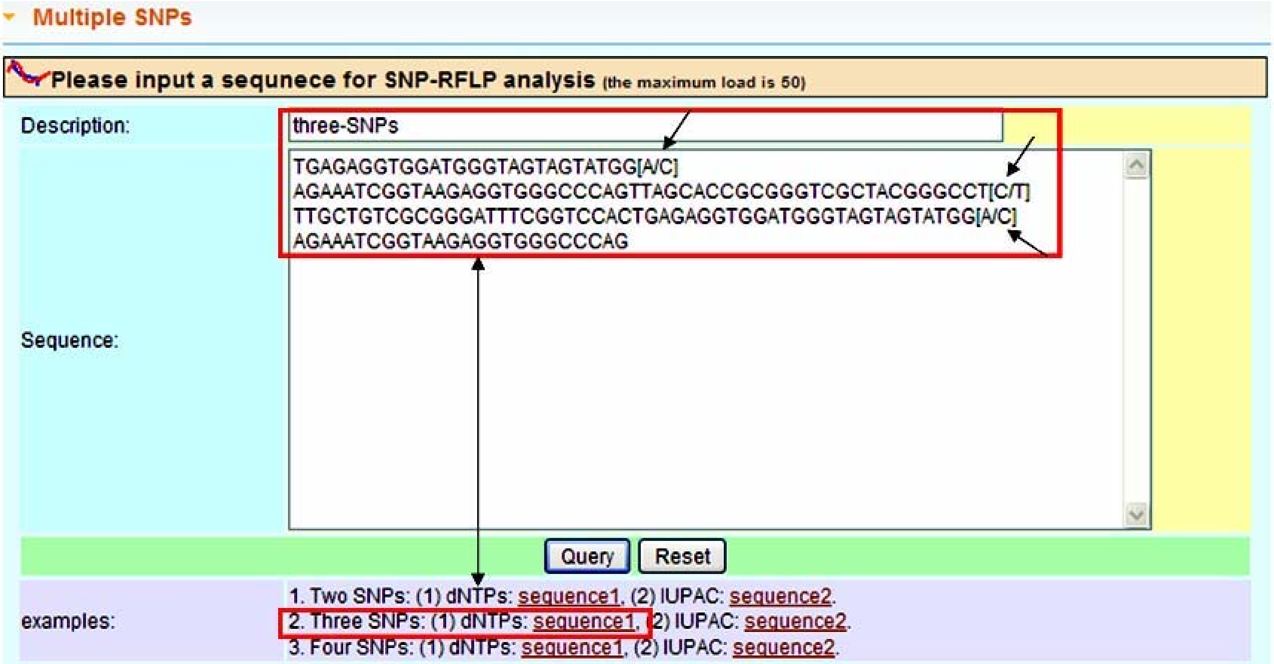

Fig. 4-3. Three SNPs within one sequence in the [dNTP1/dNTP2] format. If the SNP is marked in IUPAC code, the sequence is continued as shown in Fig. 4-4.

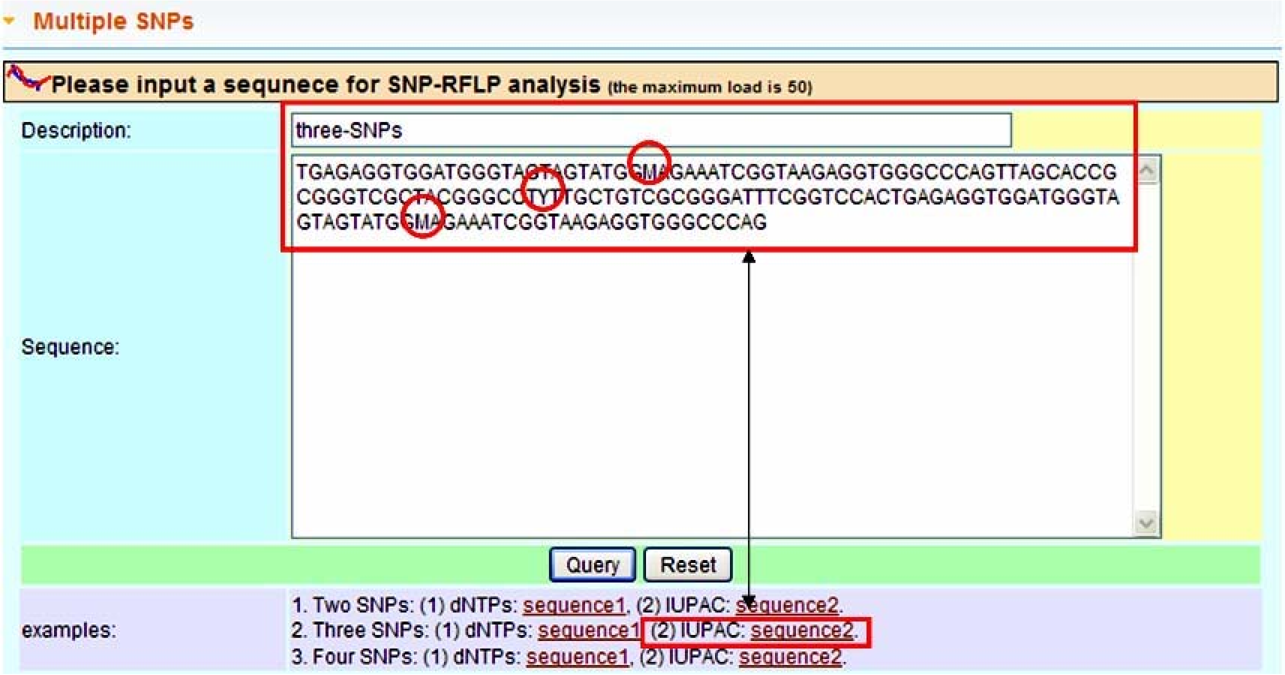

Fig. 4-4. Three SNPs within one sequence in the IUPAC format.

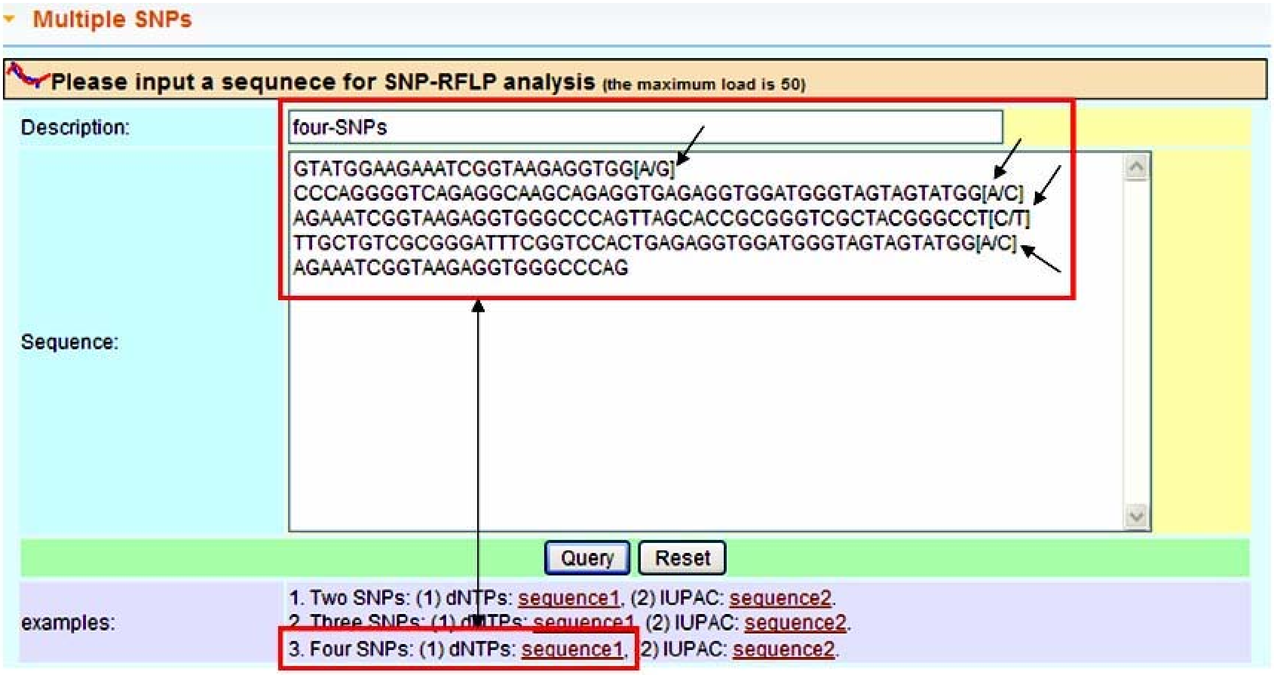

Fig. 4-5. Four SNPs within one sequence in the [dNTP1/dNTP2] format. When the sequence is too long, the part of the sequence will list in the next line automatically. The system is able to regard them as the single sequence input. If the SNP is marked in IUPAC code, the sequence is continued as shown in Fig. 4-6.

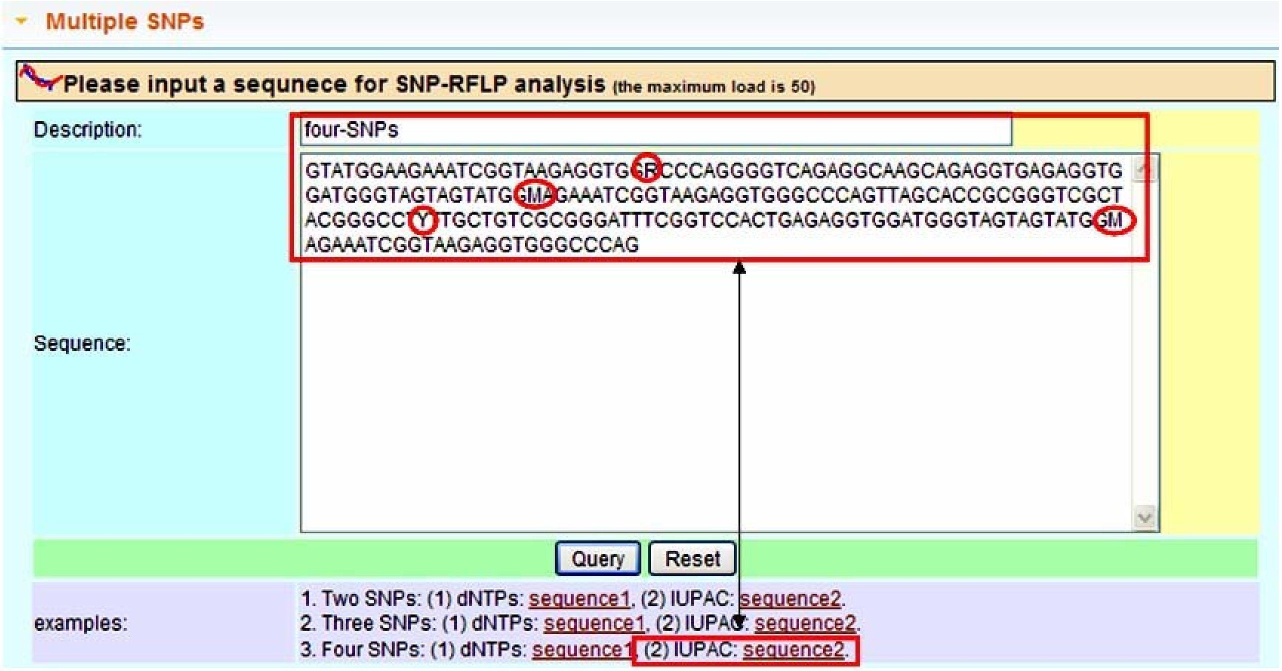

Fig. 4-6. Four SNPs within one sequence in the IUPAC format.

After inputting multiple SNPs within one sequence, users can click the "Query" button, this system will separate multiple SNPs into specific SNP fasta sequence (Fig. 4-7). User can select the interested SNPs to to perform SNP-RFLP analysis after clicking the button "Analyze sequence". The following steps are the same as the function of **(3) SNP in fasta sequence input (page 19)** and they are shown here.

In Fig. 4-7, the "All selected" button provides convenience to check all SNPs and the "Clear" button provides cancel all SNP checked.

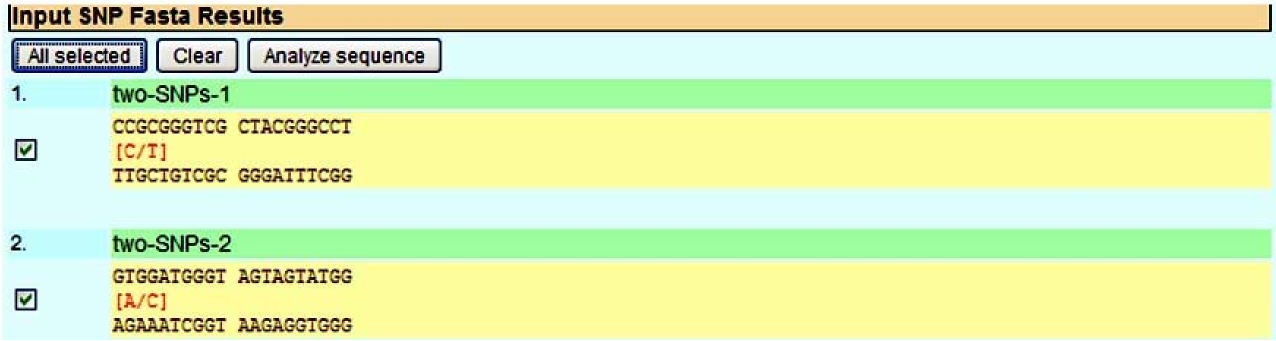

Fig. 4-7. Four SNPs within one sequence in the IUPAC format.

5. Function: GenBank accession no. for input

5.1 Overview

SNPs within the sequence information for the input accession no. of GenBank includes reference SNP ID (rs#), submitter SNP ID (ss#), accession version, HUGO gene name, local link ID (gene ID), and Local SNP ID. The classification of dbSNP in NCBI for function class (coding nonsynonymous, reference, intron, coding synonymous, locus region, mRNA UTR, and splice site), SNP class (heterozygous, indel, mixed, multinucleotide polymorphism, named locus, no variation, and snp), and heterozygosity are selectable. Furthermore, the TaqMan for non-limit, SNP500Cancer, ABI, and SNP500Cancer/ABI are available. All the information from GenBank is retrieved online for all available species (Fig. 5-1 and Fig. 5-2).

5.2 Example: Use HUGO gene name 'TP53' to analyze

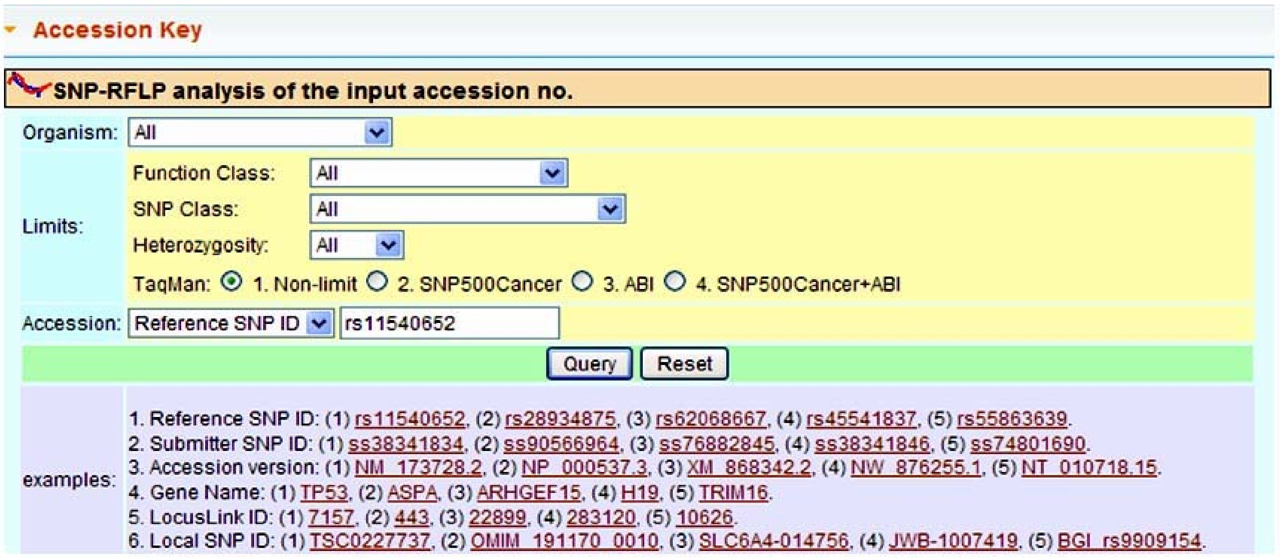

Fig. 5-1. This input options of GenBank Accession.

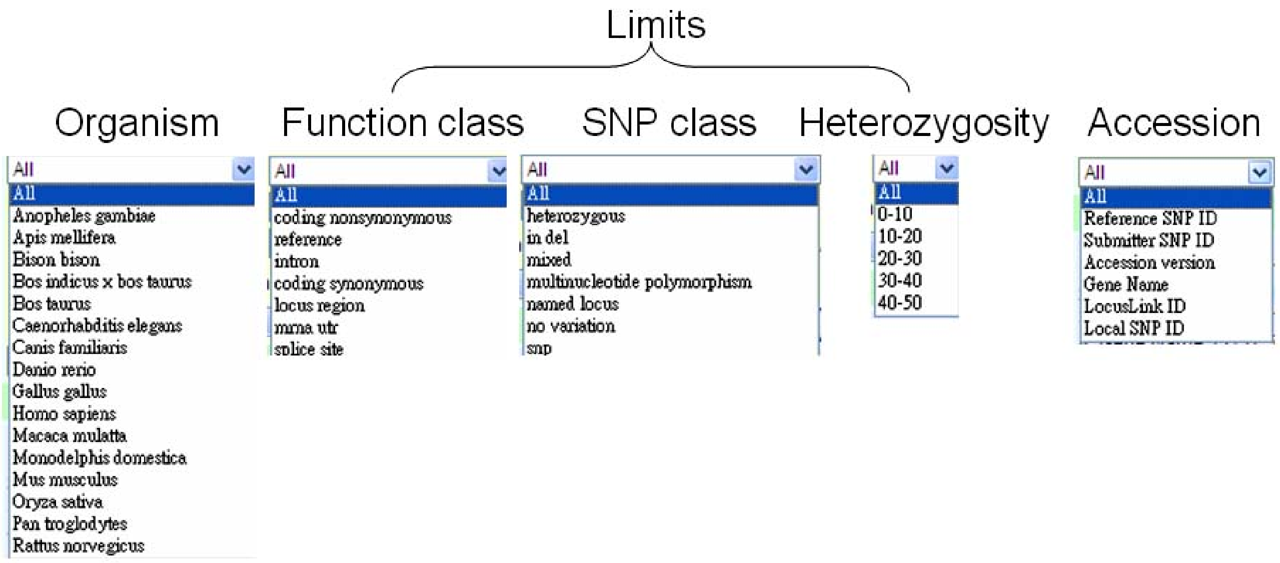

Fig. 5-2.The options for organism, limits and accession in this function of GenBank Accession.

After inputting accession no. (for example, TP53), users can click the button “Query” and it will be redirect to a waiting page (Fig. 2-12). Waiting for a moment, the results will be shown as Fig. 5-3 including: the key for input (here is Gene Name: TP53), the pages options, items for SNPs, function class, SNP class, Heterozygosity, TaqMan, and SNPs information which the same as **(2) SNP ID input** (Fig. 2-13) (page 11). The following operation (not shown) is similar as described above.

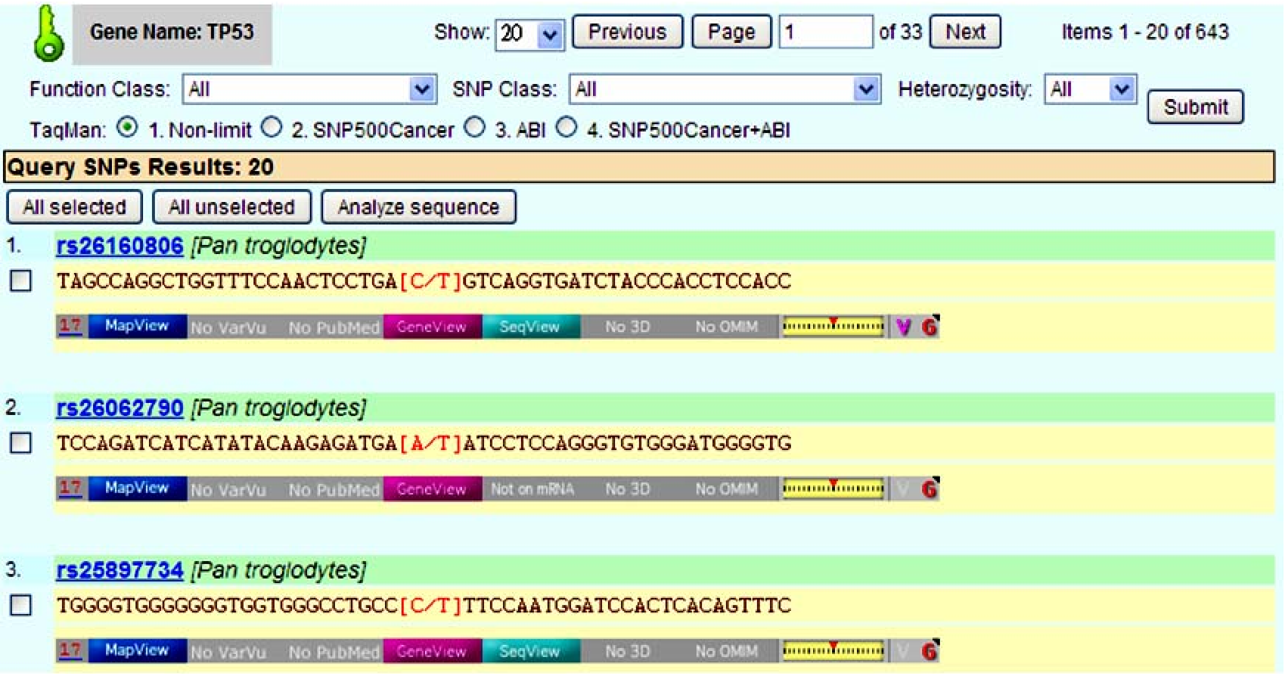

Fig. 5-3.The results for SNPs information query using HUGO gene name TP53 (here limit only show 20 SNPs and three SNPs is picked in the figure)

6. Function: TagSNP from HapMap

6.1 Overview

To reduce the necessary amount of SNPs for genotyping, it is believed that a subset of the SNP in a region (tagSNPs) can be chosen to represent most of the remaining SNP variants. The International HapMap Project is very helpful in providing the tagSNPs in the human genome for several races including YRI (Yoruba in Ibadan, Nigeria), JPT (Japanese in Tokyo, Japan), CHB (Han Chinese in Beijing, China), and CEU (CEPH; Utah residents with ancestry from northern and western Europe).

As shown in Fig. 6-1 and Fig. 6-2, the HapMap database versions, population, pairwise methods (tagger pairwise or tagger multimarkers), R square cut off, and MAF (minor allele frequency) cut off are adjustable. Position between chromosome, accession number, gene name, cytoband position, and ENCODE (ENCyclopedia Of DNA Elements) region can be queried. The tagSNPs information from HapMap is retrieved online and the mining function of RFLP restriction enzymes for the tagSNP is implemented. The current online linked tagSNP database is the HapMap Data Rel 23a/phaseII Mar08, on the NCBI B36 assembly, dbSNP b126. When users input query term (for example, Gene Name: 'BRCA2') and click the button "Query", this system will query tagSNP from HapMap and sent a waiting page (Fig. 2-12). Then, the results for HapMap database versions, population, pairwise methods, R square cut off, and MAF cut off, and tagSNP sequence information will be shown (Fig. 6-3). The following operation is similar as described above.

6.2 Example: Use HUGO gene name 'BRCA2' to analyze

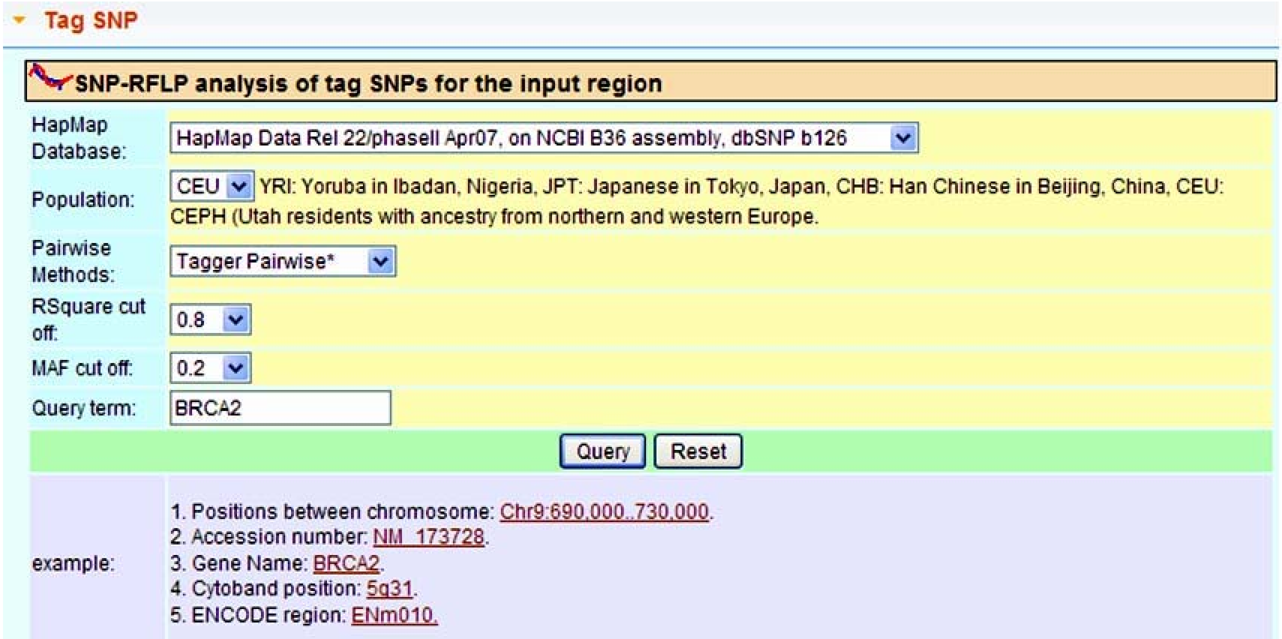

Fig. 6-1. The results for SNPs information query using HUGO gene name 'BRCA2' in the setting of the HapMap database of "HamMap Data Rel 22/phaseII Apr07, on NCBI B36 assembly, dbSNP b126 and other parameters as shown in this figure. The result is shown in Fig. 6-3.

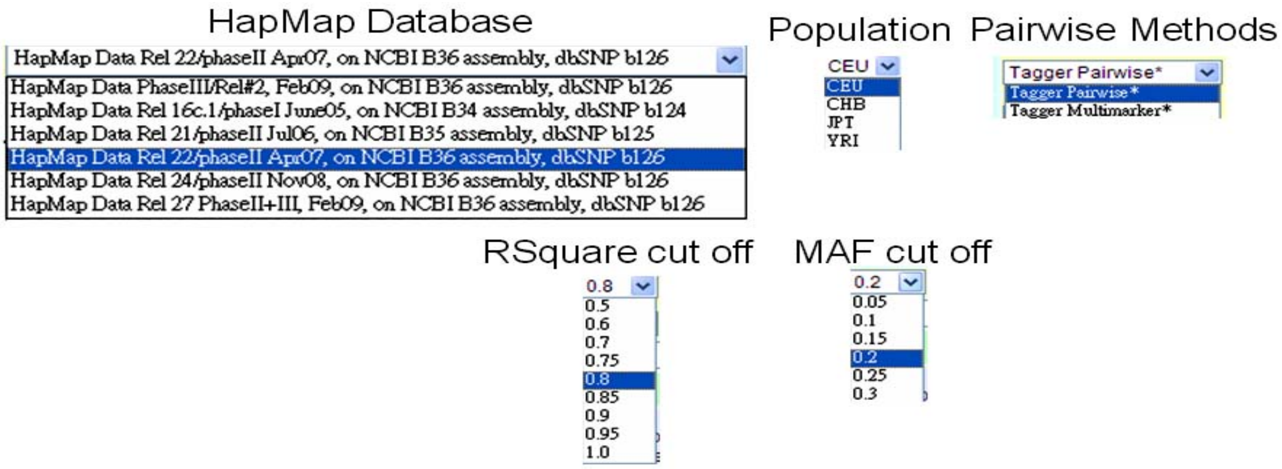

Fig. 6-2. The results for SNPs information query using HUGO gene name 'BRCA2' (here limit only show 20 SNPs and three SNPs is picked in the figure).

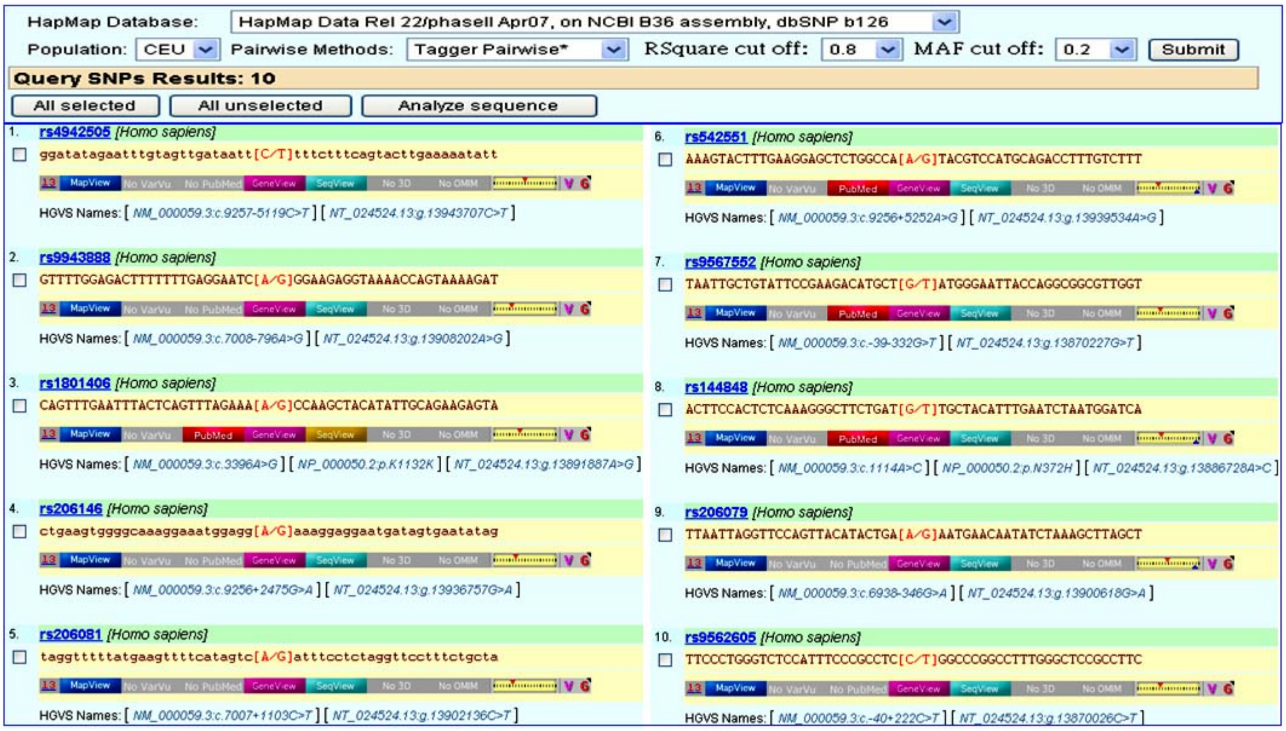

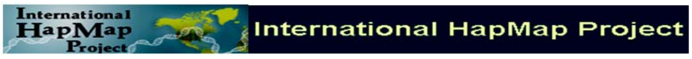

#Tue Aug 18 08:35:32 2009: HapMap tag SNPs:10 tag SNPs picked out for population CEU chr13:31787616..31871808 using the algorithm-Tagger-pairwiseTagging

#tag SNPs	Chromosome	Pos	maf
rs4942505	chr13	31861707	0.442
rs9943888	chr13	31826202	0.208
rs1801406	chr13	31809888	0.317
rs206146	chr13	31854757	0.292
rs206081	chr13	31820136	0.242
rs542551	chr13	31857534	0.263
rs9567552	chr13	31788227	0.263
rs144848	chr13	31804729	0.292
rs206079	chr13	31818618	0.467
rs9562605	chr13	31788026	0.254

## The tagSNPs provided by the HapMap are selected from different blocks. In the same block, the picked tagSNP may be different from time to time at some intervals, such as:

Interval-1 Animation *(same as top view);*

Interval-2 (partly different to Interval-1) Animation *##*

##Since the SNP-RFLPing 2 is retrieved online to the HapMap, therefore, it has the same condition as shown in the HapMap.##

Fig. 6-3. The results for tagSNPs information query using HUGO gene name 'BRCA2' as the setting for Fig. 6-1. The ten tagSNPs retrieved in SNP-RFLPing 2 (top view; Animation) is the same as shown in HapMap (bottom view; Animation). (Please click the "Animation" box to demonstrate)

7. Function: Transcript ID/miRNA

7.1 Overview

MicroRNAs (miRNAs) are a family of small RNAs that complement to the transcripts of protein coding genes, repress translation, or decrease mRNA stability. The dysfunction of miRNA can impact many targets and, thus, affect cell physiology and cancer progression. Polymorphisms in the miRNA pathway may affect gene expression, lead to change complex phenotypes, and have the potential to be disease markers for personalized medicine. In this function, the polymorphism in microRNA Target Site (PolymiRTS) database is applied to provide transcript ID and miRNA number of human and mouse to query SNPs (Fig. 7-1).

7.2 Example: Use transcript ID and miRNA number to analyze

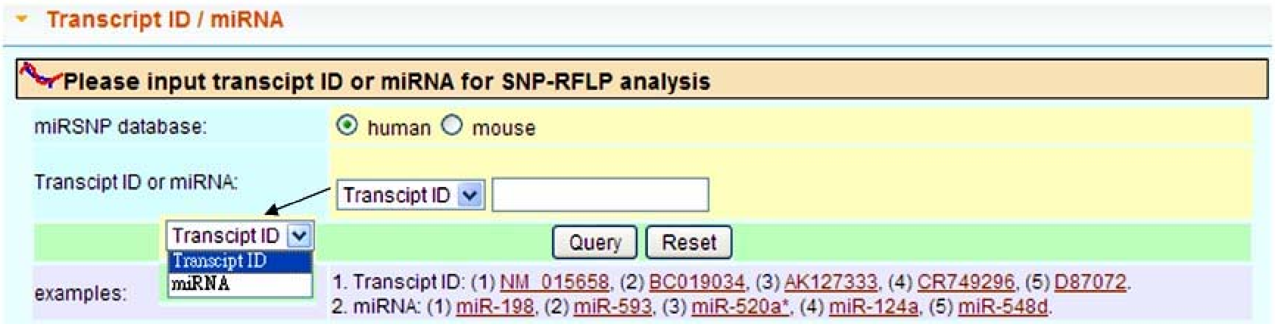

Fig. 7-1. The interface for Transcript ID/miRNA.

When users click the button "Query", this miRSNP information will be queried, and the waiting page will be shown (Fig. 7-2). After the query finished, the results for miRSNP will be shown. Fig. 7-3 and Fig. 7-4 show the result for query transcript ID "NM_015658" of human and the results for query transcript ID "miR-198" of human, respectively. The provided information contains transcript ID, SNP ID (rs#), Location (SNP position in transcript ID), Chr Pos (chromosome position), Ancestral Allele, Allele, miR ID (miRNA ID), Support, miR Site (miRNA site), and FuncClass (function class).

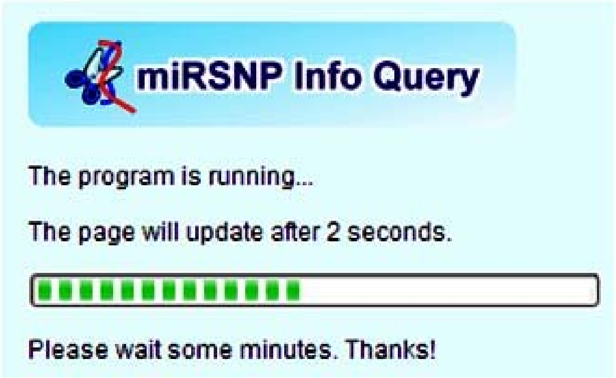

Fig. 7-2. Wait for miRSNP information query.

All selected | All unselected | Search SNP sequence

ID	Transcript ID	SNP ID	Location	Chr Pos	Ancestral Allele	Allele	miR ID	Support	miRSite	FuncClass
1 ☑	NM_015658	rs2839	2651	869549	-	G	miR-607			
						A		0	tcTTTGAAAattt	O

Fig. 7-3. The result for query transcript ID “NM_015658” of human.

All selected | All unselected | Search SNP sequence

ID	Transcript ID	SNP ID	Location	Chr Pos	Ancestral Allele	Allele	miR ID	Support	miRSite	FuncClass
1 ☑	NM_198576	rs2799073	6632	980668	G	A	miR-198<br>miR-520a*<br>miR-525	0<br>0	cTCTGGACcctgc<br>CTCTGGAccctgc	C<br>C
						G				
2 ☐	NM_022114	rs12735779	5760	3342087	G	C	miR-198	0	tcacgtCTGGACA	C
						G				
3 ☑	NM_199454	rs12735779	5703	3342087	G	C	miR-198	0	tcacgtCTGGACA	C
						G				
4 ☐	NM_000778	rs11211405	2312	47167933	A	G	miR-198	0	tgTCTGGACttgc	C
						A				
5 ☐	NM_000566	rs16865014	1163	148029723	C	C	miR-488<br>miR-198	2<br>3	atcgATCTGGAcc<br>atcgaTCTGGACc	D<br>D
						T				
6 ☐	NM_021133	rs641265	4093	180809448	T	T	miR-198			
						C		0	cCTGGACAtgtca	C
7 ☐	NM_001002295	rs12782127	2197	8156297	T	G	miR-198<br>miR-101<br>miR-199a*<br>miR-144<br>miR-493-5p	0	ataCTGGACAatg	C
						T		10<br>9	aTACTGTAcaatg<br>atacTGTACAAtg	D<br>D
8 ☐	NM_002051	rs12782127	2194	8156297	T	G	miR-198<br>miR-101<br>miR-199a*<br>miR-144<br>miR-493-5p	0	ataCTGGACAatg	C
						T		10<br>9	aTACTGTAcaatg<br>atacTGTACAAtg	D<br>D

Fig. 7-4. The results for query miRNA number “miR-198” of human. (This figure show 8 records of 10 records)

In order to query the SNP sequence information to SNP-RFLP analysis, users can check the box in the front of transcript ID and click the button “Search SNP Sequence”. A waiting page for SNPs information will be displayed. Then, users can reselect SNP once again for SNP-RFLP analysis (Fig. 7-5). The following operation is similar as described above (not shown here).

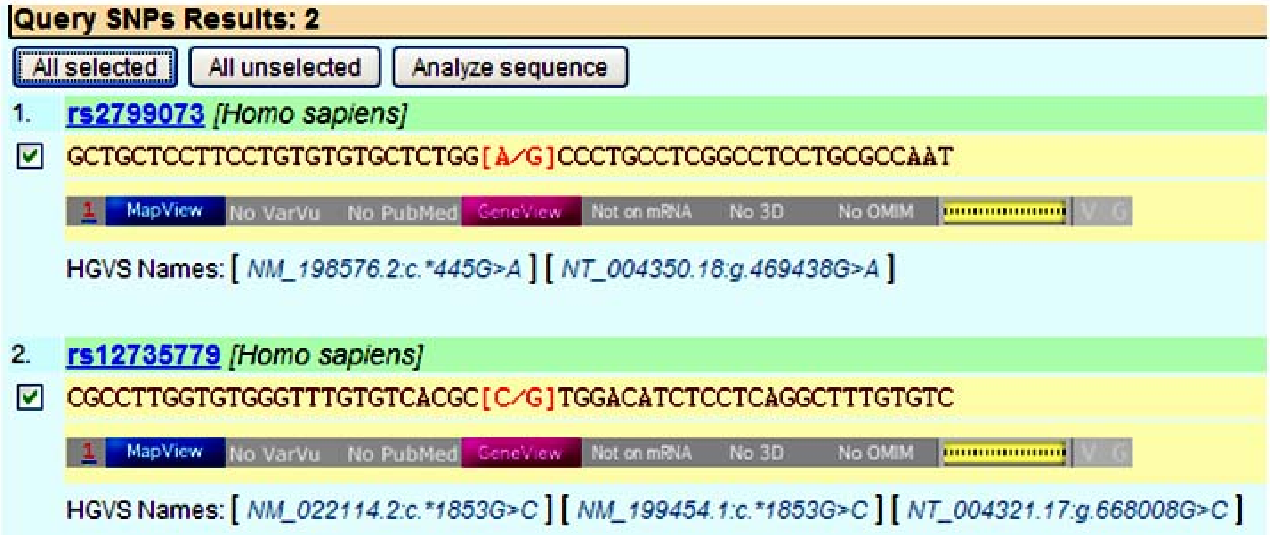

Fig. 7-5. The SNP information results for query two transcript IDs “NM_198576” and “NM_199454” of miRNA number “miR-198” of human.

8. Function: Gene Ontology-based annotation for SNPs

8.1 Overview

The Gene Ontology Browser (GO Browser; http://cgap.nci.nih.gov/Genes/GOBrowser) providing the annotation for human and mouse genes by molecular function, biological process, and cellular component. GO IDs and vocabulary terms may be input to find the genes with an interested function as well as their corresponding SNPs. In this function, CGAP database (human and mouse), Chromosomes, and GO term are available for input (Fig. 8-1). In order to get more GO terms, users can link to CGAP GO Browser (http://cgap.nci.nih.gov/Genes/GOBrowser) (Fig. 8-2).

8.2 Example: Query Gene Ontology vocabulary term

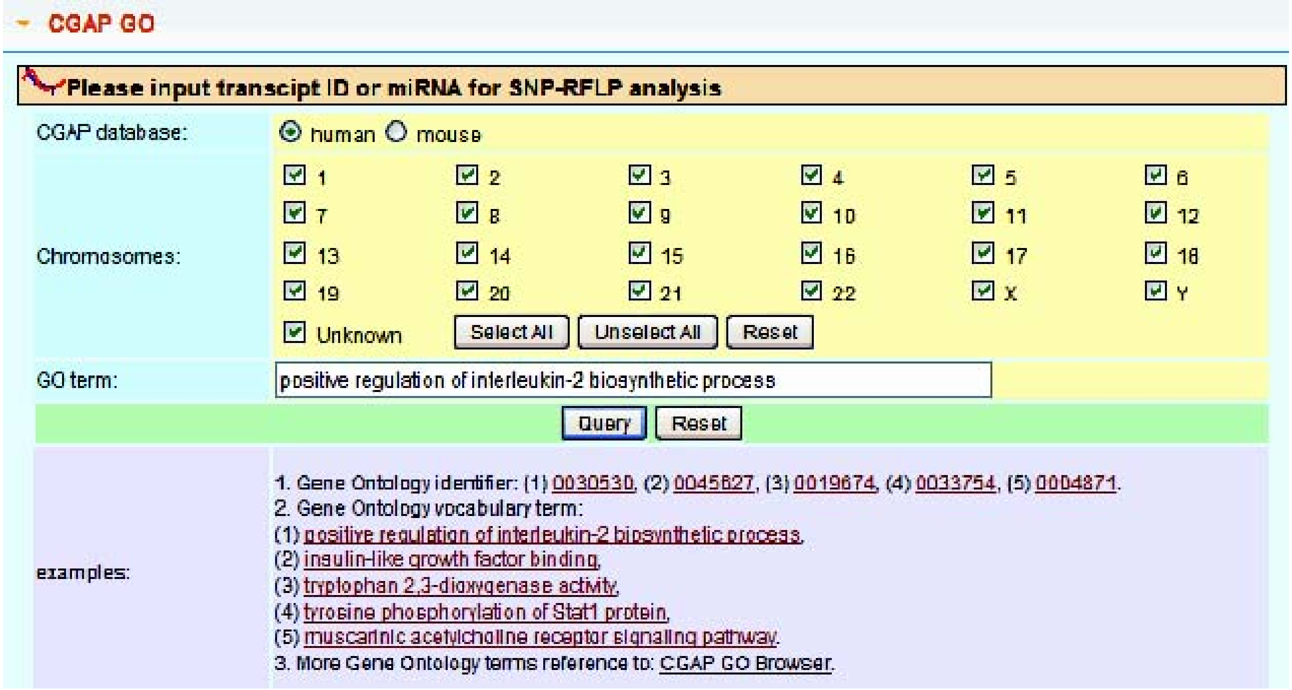

Fig. 8-1. The interface for the input of "Gene Ontology-based annotation for SNPs".

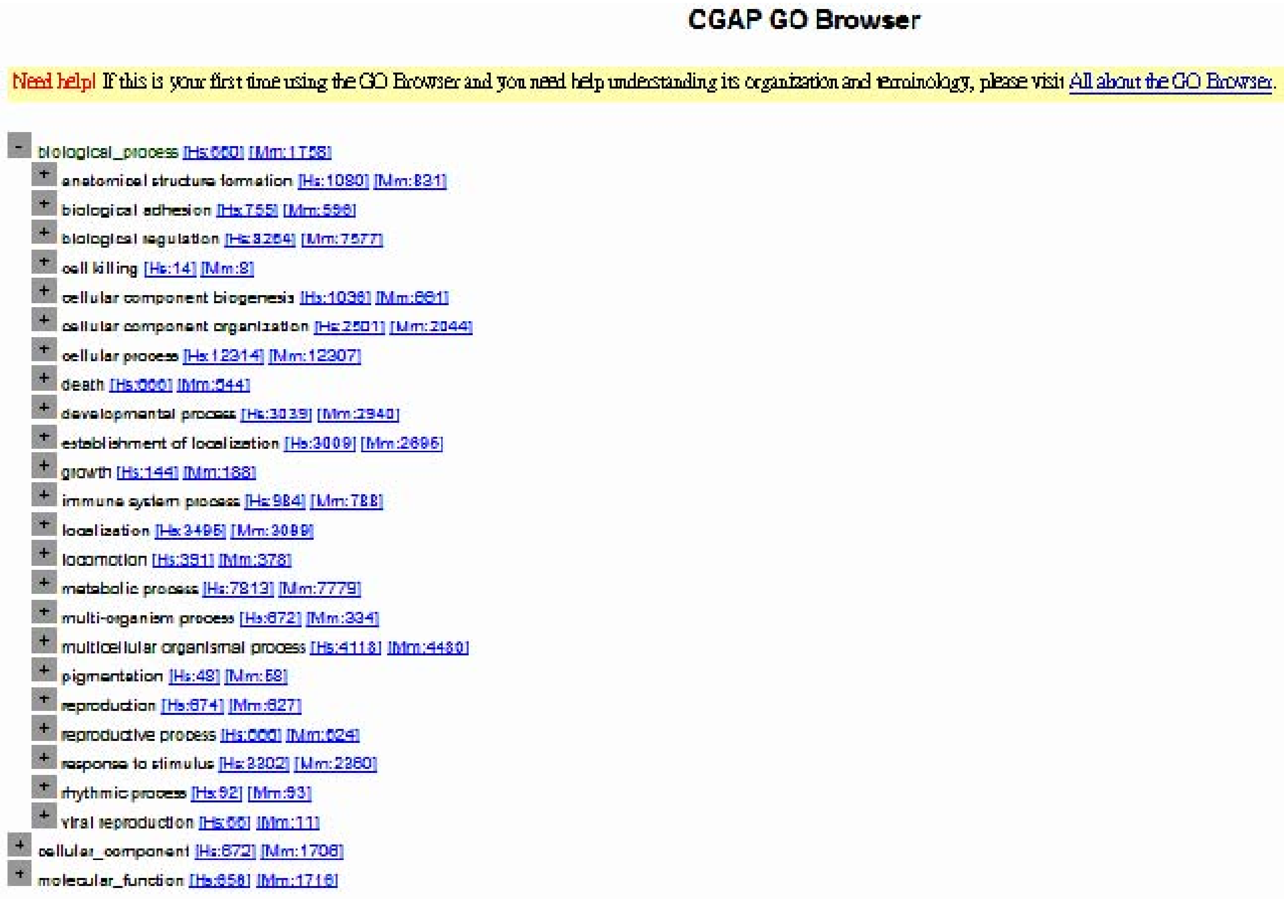

Fig. 8-2. More GO terms can be found in CGAP GO Browser. Users can copy-and-paste the interested GO term to the window of GO term to search the SNP information for specific GO term. Animation (Please click the “Animation” box to demonstrate)

When users input GO term (for example, positive regulation of interleukin-2 biosynthetic process), the waiting page for querying CGAP GO information will be shown (Fig. 8-3). Waiting for a moment, the results which contain UniGene, Gene Symbol, Locus ID, Cytoband, OMIM, sequence, and SNP Information for the query GO term will be presented (Fig. 8-4). In SNPs information, two types for SNP query are available: one is NCBI SNPs, and the other is HapMap tagSNP. Through clicking the SNPs and tagSNPs images, the SNPs information will be inquired. The SNP query function is similar as described above.

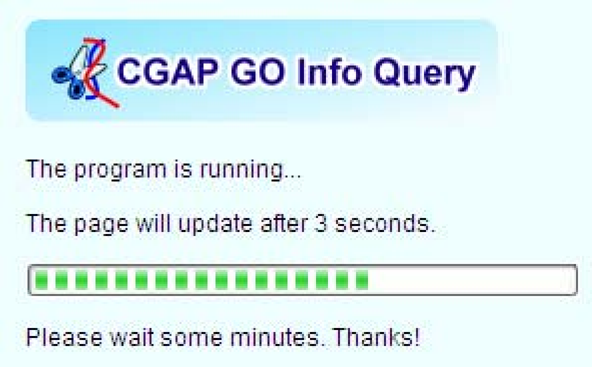

Fig. 8-3. Wait for CGAP GO information query.

ID	UniGene	Gene Symbol	Locus ID	Cytoband	OMIM	sequence	SNPs Information
1	Hs.838	CD80	941	3q13.3-q21	112203	NM_005191	SNPs
	Costimulatory molecule variant IgV-CD80 (CD80) mRNA, complete cds, alternatively spliced						tagSNPs
2	Hs.1722	IL1A	3552	2q14	147760	NM_000575	SNPs
	Interleukin 1, alpha, mRNA (cDNA clone IMAGE:5265773)						tagSNPs
3	Hs.49105	GLMN	11146	1p22.1	601749	NM_053274	SNPs
	CDNA FLJ60587 complete cds, highly similar to Glomulin						tagSNPs
4	Hs.126256	IL1B	3553	2q14	147720	NM_000576	SNPs
	Interleukin 1 beta (IL1b)						tagSNPs
5	Hs.171182	CD86	942	3q21	601020	NM_175862	SNPs
	CD86 molecule, mRNA (cDNA clone MGC:34413 IMAGE:5173789)						tagSNPs
6	Hs.401013	IRF4	3662	6p25-p23	601900	NM_002460	SNPs
	Multiple myeloma oncogene 1 (MUM1)						tagSNPs

Fig. 8-4. The results for query Gene Ontology vocabulary term "positive regulation of interleukin-2 biosynthetic process".

9. Function: File upload for input

9.1 Overview

This function is similar to **(2) SNP ID input**, and **(3) SNP in fasta sequence input** functions. SNP ID (rs# and ss#) and SNP in fasta sequence format file (*.txt) are acceptable to query the SNP-RFLP information. User can select either "SNP IDs" or "SNP fasta sequences" option, and then through the button "browse…" to get an available input file in local machine (Fig. 9-1). Finally, click the button "Put In" to perform this function. For more detail formats and steps for operating, please reference to **(2) SNP ID input**, and **(3) SNP in fasta sequence input** functions.

9.2 Example: Upload interface for SNP IDs and SNP fasta sequences

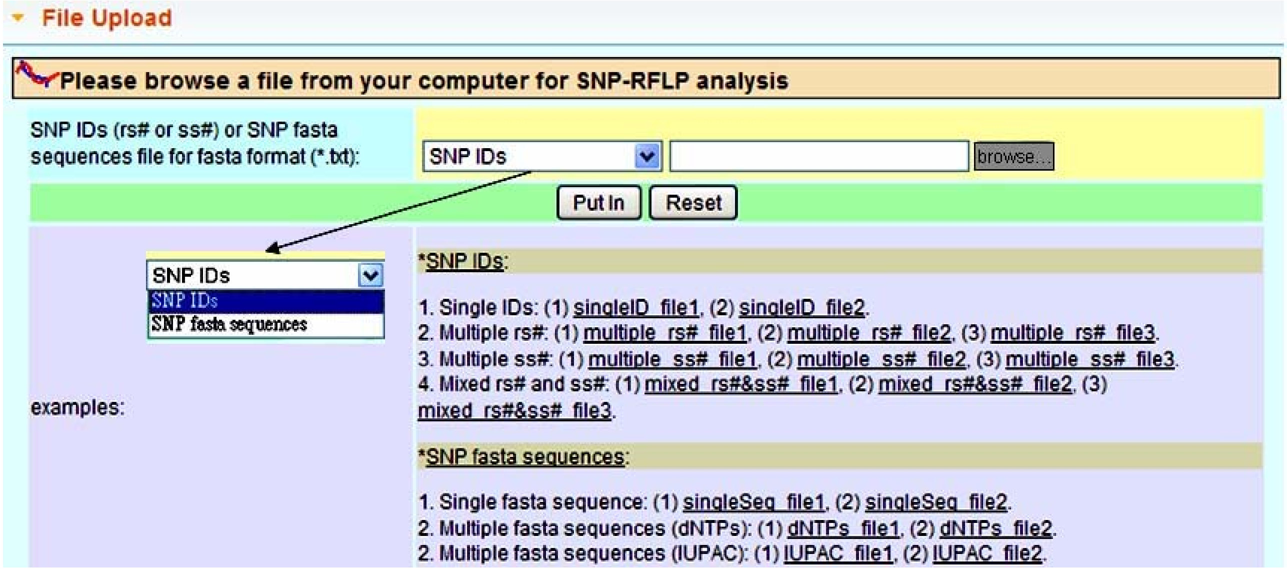

Fig. 9-1. The interface for "File upload" function.

10. Appendix

10.1 Abbreviations

- **SNP:** Single nucleotide polymorphism
- **RFLP:** Restriction Fragment Length Polymorphism
- **CGAP:** Cancer Genome Anatomy Project
- **PolymiRTS:** Polymorphism in microRNA Target Site database
- **HUGO:** Human Genome Organisation
- **IUPAC:** The International Union of Pure and Applied Chemistry
- **YRI:** Yoruba in Ibadan, Nigeria
- **JPT:** Japanese in Tokyo, Japan
- **CHB:** Han Chinese in Beijing, China
- **CEU:** CEPH (Utah residents with ancestry from northern and western Europe
- **MAF:** Major Allele Frequency
- **miRNA:**
- **GO:** Gene Ontology
- **ENCODE:** ENCyclopedia Of DNA Elements

10.2 Related links

- NCBI: http://www.ncbi.nlm.nih.gov/
- REBASE: http://rebase.neb.com/rebase/rebase.html
- dbSNP: http://www.ncbi.nlm.nih.gov/SNP/
- GenBank: http://www.ncbi.nlm.nih.gov/Genbank/
- HapMap: http://www.hapmap.org/
- SNP500Cancer: http://snp500cancer.nci.nih.gov/home_1.cfm?CFID=2676524&CFTOKEN=98627459
- PolymiRTS: http://compbio.utmem.edu/miRSNP/
- CGAP GO browser: http://cgap.nci.nih.gov/Genes/GOBrowser
- ENCODE: http://genome.ucsc.edu/ENCODE/
- Prim-SNPing: http://bio.kuas.edu.tw/prim-snping/
- Seq-SNPing: http://bio.kuas.edu.tw/Seq-SNPing/
- SNP-Flankplus: http://bio.kuas.edu.tw/snp-flankplus/snpin.jsp
- LD_2SNPing: http://bio.kuas.edu.tw/LD2SNPing/
- SNP ID-info: http://bio.kuas.edu.tw/snpid-info
